# Enantioenriched α-substituted glutamates/pyroglutamates via enantioselective cyclopropenimine-catalyzed Michael addition of amino ester imines

**DOI:** 10.3762/bjoc.17.134

**Published:** 2021-08-17

**Authors:** Zara M Seibel, Jeffrey S Bandar, Tristan H Lambert

**Affiliations:** 1 Department of Chemistry, Columbia University, New York, New York 10027, USA; 2 Department of Chemistry and Chemical Biology, Cornell University, Ithaca, New York 14853, USA

**Keywords:** Brønsted base, cyclopropenimine, enantioselective catalysis, Michael addition, pyroglutamate

## Abstract

A procedure for the enantioselective synthesis of α-substituted glutamates and pyroglutamates via a cyclopropenimine-catalyzed Michael addition of amino ester imines is described. Enantioselectivities of up to 94% have been achieved, and a variety of functional groups were found to be compatible. The impact of the catalyst structure and imine substitution is discussed. Compared to other methods, this protocol allows for a broader and more enantioselective access to pyroglutamate derivatives.

## Introduction

**Figure 1:**
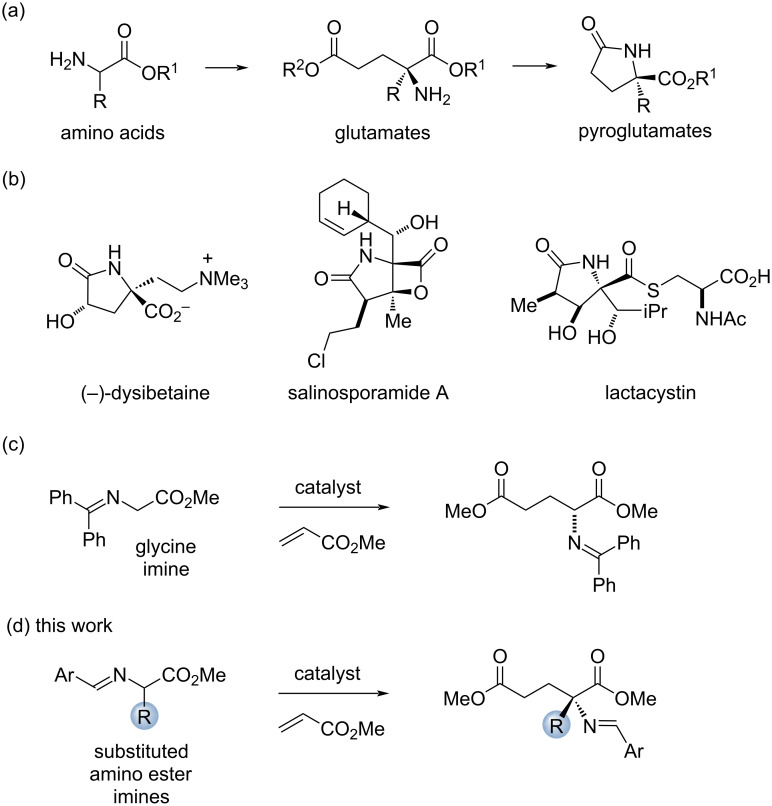
Strategy for the synthesis of glutamate and pyroglutamate derivatives and several natural products with pyroglutamate substructures.

α-Substituted glutamates have value as synthetic building blocks and as a common substructure in a number of biologically active molecules [1–5]. In addition, the lactamized derivatives of these compounds, pyroglutamates, occur in a number of well-known biologically active natural products including dysibetaine [6–12], salinosporamide A [13–18], and lactacystin [19–22] (Figure 1). Accordingly, efficient procedures to access α-substituted glutamates and pyroglutamates in enantioenriched form have been the target of numerous reports [23–27].

One of the most straightforward approaches to α-substituted glutamate derivatives is via the Michael addition of α-amino ester enolates to acrylate acceptors. These products can also be easily converted to pyroglutamates by lactamization [28–30]. Although the use of substituted amino ester derivatives for the enantioselective α-alkylation has been achieved [31], Michael reactions with these nucleophiles have met with limited success [32–39]. In terms of enantioselective catalytic strategies, Kobayashi has reported the conjugate addition of azlactones to acrylates using a calcium pybox complex, but with enantioselectivities only up to 84% ee [36]. Phase-transfer catalysis has been employed for the enantioselective addition of an alanine imine derivative, although the selectivity achieved in this case was only 64% ee [37]. In a related work, enantioselectivities of up to 90% ee were realized, but the procedure required an unusual di-*tert*-butylmethyl ester moiety and was limited solely to the alanine derivative [38]. Finally, a Baylis–Hillman-type approach has been employed to realize enantioselective reactions, albeit with a limited scope of substituents at the quaternary carbon [39]. Thus, the development of a general strategy for the enanantioselective conjugate addition of amino acid derivatives for this reaction remains an unmet goal.

Our group previously described a chiral cyclopropenimine catalyst that displayed outstanding reactivity for addition reactions of glycine imines [40–41]. We hypothesized that this reactivity might be sufficient to overcome the reactivity limitations of pronucleophiles derived from other α-amino esters [42]. In this paper, we describe the use of cyclopropenimine catalysis for the enantioselective catalytic Michael reaction of α-substituted amino ester imines.

## Results and Discussion

To optimize this process, we selected the addition of alanine imine **1** to methyl acrylate as our test reaction (Table 1). We found that the previously reported cyclopropenimine **4** catalyzed this transformation with 90% conversion and 84% ee in 24 hours at ambient temperature (Table 1, entry 1). The desired Michael adduct **2** was generated in a 4:1 ratio along with the cycloadduct **3** [43], which we had not observed in our previous study of glycinate imine substrates. The aminoindanol-derived catalyst **5** was more reactive and resulted in improved enantioselectivity (89% ee), but afforded the same 4:1 ratio of the Michael adduct to cycloaddition product (Table 1, entry 2). Interestingly, the larger ring-containing catalyst **6** improved this ratio somewhat to 6:1 while retaining the enantiomeric ratio, albeit at the expense of reactivity (Table 1, entry 3). Incorporation of additional unsaturation (catalyst **7**) improved the reactivity somewhat but was detrimental to enantioselectivity (Table 1, entry 4), while changing the relative stereochemistry of the hydroxy substituent resulted in an inactive catalyst (**8**, entry 5 in Table 1). Likewise, catalysts such as **9** lacking a hydrogen-bonding substituent were not active (Table 1, entry 6).

**Table 1:** Optimization of the cyclopropenimine-catalyzed addition of alanine imine **1** to methyl acrylate.

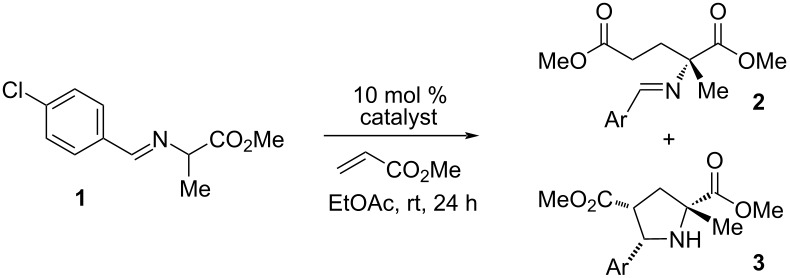						

entry	catalyst	solvent	conc. (M)	conv (%)^a^	% ee^b^	**2**:**3**^c^

1	**4**	EtOAc	0.25	90	84	4:1
2	**5**	EtOAc	0.25	>95	89	4:1
3	**6**	EtOAc	0.25	62	89	6:1
4	**7**	EtOAc	0.25	75	70	6:1
5	**8**	EtOAc	0.25	<5	–	–
6	**9**	EtOAc	0.25	<5	–	–
7	**5**	PhMe	0.25	>95	92	3:1
8	**5**	TBME	0.25	>95	91	3:1
9	**5**	dioxane	0.25	34	86	3:1
10	**5**	toluene	0.25	>95	92	2.5:1
11	**5**	ether	0.25	>95	93	4:1
12	**5**	ether	0.35	>95^d^	91	4:1

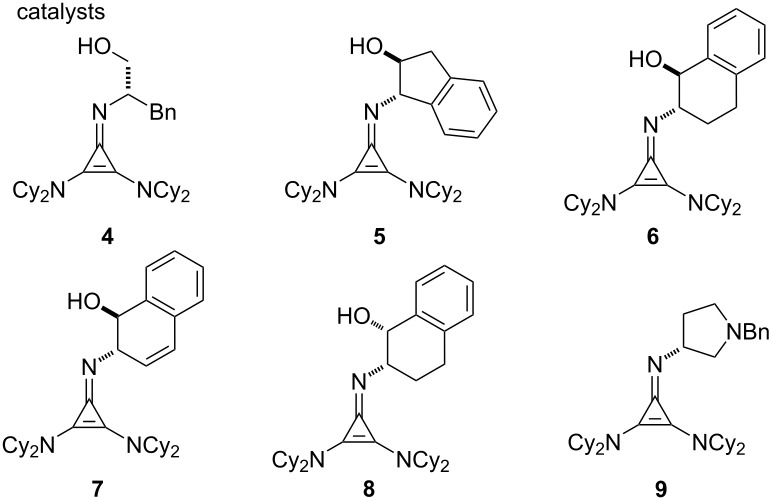
^a^Determined by ^1^H NMR versus Bn_2_O as an internal standard. ^b^Determined by HPLC. ^c^Determined by ^1^H NMR on crude reaction mixtures. The minor products **3** were isolated as single diastereomers, but the % ee was not determined. ^d^Reaction time 7 h.

With the identification of cyclopropenimine **5** as our optimal catalyst [44], we examined the effect of the reaction medium. Solvents such as benzene (Table 1, entry 7), TBME (Table 1, entry 8), and toluene (Table 1, entry 10) produced reactivities on par with ethyl acetate and approximately equal enantioselectivities but resulted in slightly worse ratios of **2** and **3**. 1,4-Dioxane was notably detrimental to the reactivity and selectivity (Table 1, entry 9). On the other hand, the use of diethyl ether as solvent resulted in a high reactivity, enantioselectivity of 93%, and no erosion of the Michael product to cycloadduct ratio (Table 1, entry 11). Finally, increasing the concentration of the reaction shortened the reaction time without significant detriment to selectivity (Table 1, entry 12).

**Figure 2:**
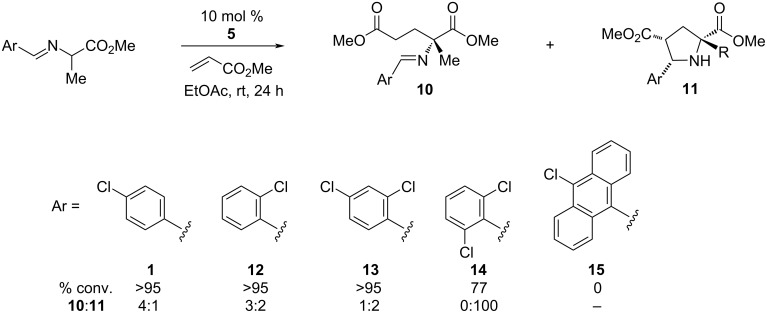
Effect of the aryl substituent on reaction efficiency and selectivity.

We also examined the impact of the imine aryl substituent on the reaction efficiency, stereoselectivity, and selectivity for the Michael addition versus cycloaddition (Figure 2). The optimal substituent in this regard proved to be *p*-chlorophenyl **1**, which resulted in the yield and selectivities using catalyst **5** as already discussed in Table 1. The *o-*chlorophenyl imine **12** was equally reactive, but led to a greater production of the cycloadduct. Interestingly, the 2,4-dichlorophenyl imine **13** resulted in a 2:1 ratio in favor of the cycloadduct, which suggests that this selectivity has a significant electronic sensitivity. On the other hand, the 2,6-dichlorophenyl imine **14** led to exclusive formation of the cycloadduct. Other, more elaborate aryl imines such as chloroanthracenyl **15** proved to be unproductive.

With the optimized conditions in hand, we proceeded to examine the substrate scope of this protocol (Table 2). Remarkably, changing the substituent on the amino ester imine substrate from methyl (Table 2, entry 1) to ethyl (Table 2, entry 2) resulted in a significant increase of reaction time and reduction of yield and enantioselectivity. Indeed, 20 mol % of the catalyst were required to realize a 48 h reaction time in the latter case. This hindrance of reaction efficiency was exacerbated by further extension of the alkyl substituent to *n-*propyl, *n*-butyl, or *n*-hexyl (Table 2, entries 3–5). Underscoring the sensitivity of this reaction to steric encumbrance with this substituent, we found that isobutyl or benzyl (Table 2, entries 6 and 7) further reduced the enantioselectivity and an isopropyl completely suppressed reactivity (Table 2, entry 8). On the other hand, allyl (Table 2, entry 9) and propargyl (Table 2, entry 10) groups proved to viable substituents, leading to the products in good yield and high enantioselectivities. Of course, these two functional groups provide convenient handles for derivatization and so represent important achievements for this method. In terms of additional functionality, we found that a thioether substrate could be engaged with reasonably good efficiency and enantioselectivity (Table 2, entry 11). On the other hand, while a nitrile was compatible with the reaction (Table 2, entry 12), the incorporation of this substituent led to a nearly total loss in selectivity.

**Table 2:** Substrate scope of amino ester imine additions to methyl acrylate.

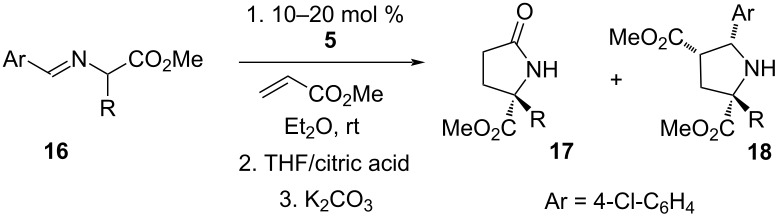						

entry	product	**5** (mol %)	time (h)	**17** yield (%)^a^	**17** % ee^b^	**18** yield (%)^a^

1	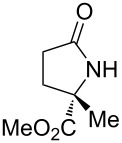 **17a**	10	16	73	93	19
2	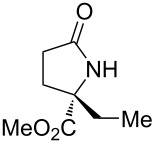 **17b**	20	48	70	87	11
3	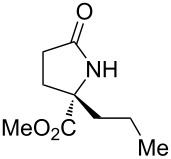 **17c**	20	48	67	82	13
4	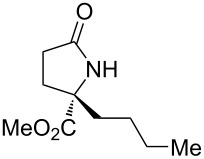 **17d**	20	48	54	82	11
5	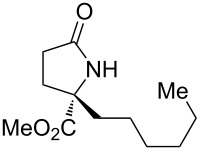 **17e**	20	48	46	80	9
6	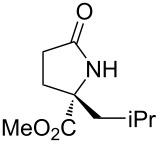 **17f**	20	48	62	77	10
7	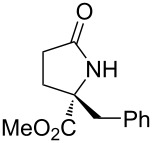 **17g**	15	48	77	75	21
8	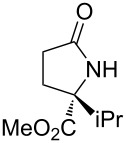 **17h**	20	48	0	–	0
9	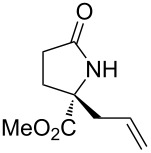 **17i**	10	48	69	94	23
10	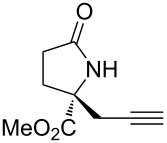 **17j**	10	16	64	88	20
11	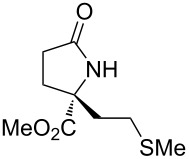 **17k**	10	16	76	84	19
12	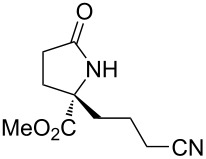 **17l**	10	16	75	16	16

^a^Calculated based on isolated and purified material. The minor products **18** were isolated as single diastereomers, but the % ee was not determined. ^b^Determined by HPLC or by ^1^H NMR using Eu(hfc)_3_ as a chiral shift reagent.

**Figure 3:**
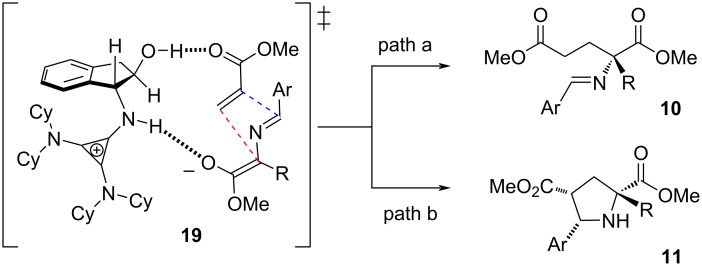
Proposed transition state model.

Although we have not examined this specific reaction computationally, it is reasonable to expect that it shares many similarities to the corresponding glycine imine addition we previously reported [45], for which a detailed transition state model was developed. In that study, it was determined that the reaction proceeds via several competing low-energy transition states involving both O–H and N–H enolate binding modes, *E* and *Z* enolate isomers, and a range of H-bonding and other noncovalent organizational interactions. This complexity makes the detailed prediction of the transition state organization for the current process very challenging. However, we propose that the general structure **19** shown in Figure 3 is a reasonable representation of one of the likely pathways (the major ambiguities being enolate geometry and N–H vs O–H binding). From this transition state, addition of the enolate to the acrylate followed by rapid proton transfer would lead to the glutamate derivative **10** (path a, red dashed line). A competing pathway involving bond formation between the acrylate α-carbon and the imine carbon, either in a concerted fashion or via subsequent addition of a putative acrylate enolate intermediate, would lead to the cycloaddition byproduct **11** (path b, red and blue dashed lines). It should be noted that cyclopropenimine catalysts do not promote the cyclization of **10** to **11**. From this model, it is understandable that increasing the electron deficiency of the aryl (Ar) substituent would increase the level of cycloadduct, while greater steric encumbrance from this substituent would bolster the Michael addition pathway, as illustrated by the data from Figure 2.

## Conclusion

In conclusion, we have developed an improved method for the synthesis of enantioenriched α-substituted glutamates and pyroglutamates using cyclopropenimine catalysis. This protocol offers significantly faster reaction rates, increased enantioselectivities, and broader substrate scope than previous efforts. However, this chemistry remains quite sensitive to structural modifications, and thus there remains significant room for further development. Nevertheless, this work provides a convenient means to access a variety of these important structural motifs.

## Supporting Information

File 1 Experimental details, characterization data, spectra, and HPLC traces.
