# Supplementary material for: Enantioenriched α-substituted glutamates/pyroglutamates via enantioselective cyclopropenimine-catalyzed Michael addition of amino ester imines
Source: Beilstein J Org Chem. 2021 Aug 17;17:2077–84. doi: 10.3762/bjoc.17.134 (PMC8381810; doi:10.3762/bjoc.17.134)
Supplement: File 1 — Experimental details, characterization data, spectra, and HPLC traces. [file Beilstein_J_Org_Chem-17-2077-s001.pdf]

BEILSTEIN JOURNAL OF ORGANIC CHEMISTRY

Supporting Information

for

Enantioenriched α-substituted glutamates/pyroglutamates via enantioselective cyclopropenimine-catalyzed Michael addition of amino ester imines

Zara M. Seibel, Jeffrey S. Bandar and Tristan H. Lambert

Beilstein J. Org. Chem. **2021,** *17,* 2077–2084. doi:10.3762/bjoc.17.134

Experimental details, characterization data, spectra, and HPLC traces

License and Terms: This is a supporting information file under the terms of the Creative Commons Attribution License (https://creativecommons.org/licenses/by/4.0). Please note that the reuse, redistribution and reproduction in particular requires that the author(s) and source are credited and that individual graphics may be subject to special legal provisions.
The license is subject to the *Beilstein Journal of Organic Chemistry* terms and conditions: (https://www.beilstein-journals.org/bjoc/terms)

General information: Organic solutions were concentrated using a Büchi rotary evaporator. Commercial reagents were used as provided. Flash column chromatography was performed employing 32–63 μm silica gel (Dynamic Adsorbents Inc). Thin-layer chromatography (TLC) was performed on silica gel 60 F254 plates (EMD).

^{1}H and ^{13}C NMR were recorded in $CDCl_3$ on Bruker DRX-400 spectrometers. Data for ^{1}H NMR are reported as follows: chemical shift (δ ppm), multiplicity (s = singlet, brs = broad singlet, d = doublet, t = triplet, q = quartet, m = multiplet), coupling constant (Hz), integration, and assignment. Data for ^{13}C NMR are reported in terms of chemical shift. Low- resolution mass spectra (LRMS) were acquired on a JEOL JMS-LCmate liquid chromatography mass spectrometer system using CI^{+} ionization technique.

Synthesis of cycloadducts and lactams:

A vial was charged with cyclopropenimine catalyst (0.1–0.2 equiv) and then imine (0.2 mmol, 1 equiv), ether (0.8 mL, 0.25 M), and methyl acrylate (0.054 mL, 0.6 mmol, 3 equiv) were added. The vial was capped and sealed with parafilm and then stirred for 16–48 hours at room temperature. At that time the mixture was concentrated and the residue was dissolved in a mixture of 2 mL THF and 2 mL 0.5 M aqueous citric acid. After stirring for one hour the mixture was diluted with water (20 mL) and extracted twice with ether (2 × 20 mL). The ether layers were combined, dried, and concentrated to furnish the crude cycloadduct, which was purified by silica gel chromatography. Meanwhile, potassium carbonate (414 mg, 3 mmol, 30 equiv) was added to the aqueous layer noted above and the mixture was stirred for 3 hours before being extracted with DCM (3 × 20 mL). The combined organic layers were dried and concentrated. After purification by silica gel chromatography the pure lactams were isolated. Enantiomeric excess was determined

by HPLC analysis of either the imine or lactam products, or by ^{1}H NMR analysis of the lactam in the presence of europium(III) tris[3-(heptafluoropropylhydroxymethylene)-*d*-camphorate] as a chiral shift reagent.

Procedure for determination of enantiomeric excess via use of chiral shift reagent Eu(hfc)$_3$: Prior to use, $CDCl_3$ was dried with sodium sulfate and filtered through a silica plug. 0.5–2 mg of the lactam was dissolved in 0.6 mL $CDCl_3$ under Ar. Approximately 5 mg of Eu(hfc)$_3$ was added as a solution in $CDCl_3$ (this solution was around 50 mg/mL and was filtered through a syringe filter to remove insoluble paramagnetic impurities). A ^{1}H NMR spectrum was obtained. If the peaks were not sufficiently separated, more Eu(hfc)$_3$ was added until separation was sufficient. Enantiomeric excess was determined by comparison of the methyl ester peaks, which were shifted downfield and cleanly separated. NMR spectra are provided of both the racemic mixture and chiral substrate; however, the peaks are not always at the exact same chemical shift due to slightly differing ratios of substrate to Eu(hfc)$_3$.

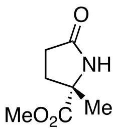

17a

Methyl 2-methyl-5-oxopyrrolidine-2-carboxylate (17a): Prepared as described in the general procedure and isolated in 73% yield as a clear oil. ^{1}H NMR (400 MHz, $CDCl_3$): 5.9 (br s, 1H, N*H*), 3.76 (s, 3H, CO_2CH_3), 2.50-2.57 (m, 1H, alkyl*H*), 2.41 (t, *J*= 7.6 Hz, 2H, C(O)CH_2), 1.99-2.07 (m, 1H, alkyl*H*), 1.52 (s, 3H, CCH_3). ^{13}C NMR (101 MHz, $CDCl_3$) δ 176.62, 174.33, 62.05, 52.77, 32.20, 29.83, 25.89. LRMS (APCI+): exact mass calc'd for $C_7H_{12}NO_3$ $[M+1]^+$ requires *m/z* 158.07,

found *m/z* 157.91. HPLC analysis (performed on the imine intermediate): Chiralpak OJ-H (Hex/iPrOH = 90/10, 1 mL/min, 254 nm), 11.5 min (major), 19.6 min (minor), 93% ee.

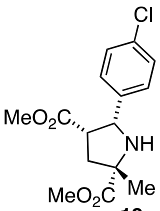

18a

Dimethyl 2-methyl-5-(4-chlorophenyl)pyrrolidine-2,4-dicarboxylate (18a): This compound was synthesized similar to above; however, it remained in the aqueous layer after the ether extraction of the citric acid mixture and so was isolated following lactamization as a white solid in 19% yield. ^{1}H NMR (400 MHz, $CDCl_3$): 7.30-7.22 (m, 4H, Ar*H*), 4.68 (d, *J* =7.6 Hz, 1H, ArC*H*), 3.83 (s, 3H, CO_2CH_3), 3.40-3.33 (m, 1H, $MeCO_2CH$), 3.26 (s, 3H, CO_2CH_3), 2.73 (dd, *J* = 13.6 Hz, 5.2 Hz, 1H, CH_2), 2.07 (dd, *J* = 13.6 Hz, 7.6 Hz, 1H, CH_2), 1.517 (s, 3H, CH_3). ^{13}C NMR (101 MHz, $CDCl_3$) 176.36, 172.75, 137.63, 133.40, 128.39, 128.19, 65.82, 64.15, 52.65, 51.37, 50.15, 40.07, 27.35. LRMS (APCI+): exact mass calc'd for $C_{15}H_{18}ClNO_4$ $[M+1]^+$ requires *m/z* 312.09, found *m/z* 312.17.

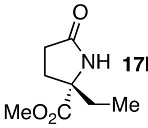

17b

Methyl 2-ethyl-5-oxopyrrolidine-2-carboxylate (17b): Prepared as described in the general procedure with a 20% catalyst loading and 48 h reaction time. The title compound was isolated as a white solid in 70% yield. ^{1}H NMR (400 MHz, $CDCl_3$): ^{1}H NMR (400 MHz, $CDCl_3$): 6.15 (br s, 1H, N*H*), 3.76 (s, 3H, CO_2CH_3), 2.54-2.44 (m, 1H, alkyl*H*), 2.38 (t, *J* = 8.0 Hz, 2H, alkyl*H*), 2.12-2.02 (m, 1H, alkyl*H*), 1.95-1.85 (m, 1H, alkyl*H*), 1.85-1.76 (m, 1H, alkyl*H*), 0.90 (t, *J* = 7.5 Hz, 3H, CH_3). ^{13}C NMR (101 MHz, $CDCl_3$) δ 176.75, 173.92, 66.10, 52.60, 32.27, 30.11, 29.72, 8.38.

LRMS (APCI+): exact mass calc'd for $C_8H_{13}NO_3$ $[M+1]^+$ requires *m/z* 172.09, found *m/z* 172.22. Enantiometric excess (87% ee) was determined by the addition of chiral shift reagent.

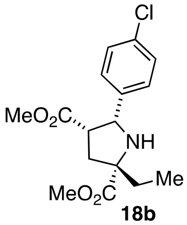

18b

Dimethyl 2-ethyl-5-(4-chlorophenyl)pyrrolidine-2,4-dicarboxylate (18b): Prepared as described in the general procedure with a 20% catalyst loading and 48 h reaction time. The title compound was isolated as a white solid in 11% yield. ^{1}H NMR (400 MHz, $CDCl_3$): 7.25 (q, *J* = 8.7 Hz, 4H, Ar*H*), 4.55 (d, *J* =7.2 Hz, 1H, ArC*H*), 3.82 (s, 3H, CO_2CH_3), 3.25 (s, 3H, CO_2CH_3), 3.30-3.23 (m, 1H, $MeCO_2CH$), 2.70 (dd, *J* = 13.7 Hz, 4.6 Hz, 1H, CH_2), 2.06 (dd, *J* = 13.7 Hz, 7.5 Hz, 1H, CH_2), 1.94-1.79 (m, 1H, CH_2CH_3), 1.75-1.62 (m, 1H, CH_2CH_3), 0.91 (t, *J*= 7.4Hz, 3H, CH_2CH_3). ^{13}C NMR (101 MHz, $CDCl_3$) δ 176.09, 172.99, 137.90, 133.29, 128.37, 128.12, 70.04, 64.69, 52.39, 51.30, 50.25, 39.27, 33.74, 9.39. LRMS (APCI+): exact mass calc'd for $C_{16}H_{20}ClNO_4$ $[M+1]^+$ requires *m/z* 326.11, found *m/z* 326.20.

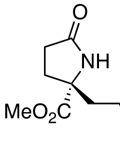

17c

Methyl 2-propyl-5-oxopyrrolidine-2-carboxylate (17c): Prepared as described in the general procedure with a 20% catalyst loading and 48 h reaction time. The title compound was isolated as a white solid in 67% yield. ^{1}H NMR (400 MHz, $CDCl_3$): 6.04 (br s, 1H, N*H*), 3.76 (s, 3H, CO_2CH_3), 2.53-2.44 (m, 1H, alkyl*H*), 2.40-2.33 (m, 2H, alkyl*H*), 2.13-2.04 (m, 1H, alkyl*H*), 1.88-1.78 (m, 1H, alkyl*H*), 1.76-1.66 (m, 1H, alkyl*H*), 1.35-1.20 (m, 1H, CH_2CH_3) 0.93 (t, *J* = 7.6 Hz, 3H, CH_3). ^{13}C NMR (101 MHz, $CDCl_3$) δ 176.64, 174.00, 65.58, 52.61, 41.61, 30.72, 29.62, 17.57,

14.02. LRMS (APCI+): exact mass calc'd for $C_9H_{15}NO_3$ $[M+1]^+$ requires *m/z* 186.11, found *m/z* 186.18. Enantiometric excess (82% ee) was determined by the addition of chiral shift reagent.

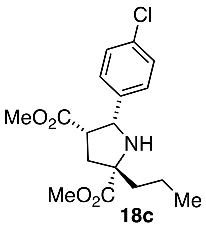

18c

Dimethyl 2-propyl-5-(4-chlorophenyl)pyrrolidine-2,4-dicarboxylate (18c): Prepared as described in the general procedure with a 20% catalyst loading and 48 h reaction time. The title compound was isolated as a white solid in 13% yield. ^1H NMR (400 MHz, $CDCl_3$): 7.29-7.21 (m, 4H, Ar*H*), 4.56 (d, *J* =7.2 Hz, 1H, ArC*H*), 3.81 (s, 3H, CO_2CH_3), 3.25 (s, 3H, CO_2CH_3), 3.30-3.23 (m, 1H, $MeCO_2CH$), 3.01 (br s, 1H, N*H*), 2.70 (dd, *J* = 13.6 Hz, 4.8 Hz, 1H, CH_2), 2.06 (dd, *J* = 13.6 Hz, 7.6 Hz, 1H, CH_2), 1.87-1.76 (m, 1H, $CH_2CH_2CH_3$), 1.66-1.55 (m, 1H, $CH_2CH_2CH_3$), 1.52-1.41 (m, 1H, CH_2CH_3), 1.22-1.10 (m, 1H, CH_2CH_3), 0.91 (t, *J*= 7.2Hz, 3H, CH_2CH_3). ^{13}C NMR (101 MHz, $CDCl_3$) δ 176.25, 172.99, 137.88, 133.29, 128.36, 128.13, 69.52, 64.76, 52.39, 51.29, 50.21, 43.28, 39.66, 18.43, 14.20. LRMS (APCI+): exact mass calc'd for $C_{17}H_{22}ClNO_4$ $[M+1]^+$ requires *m/z* 340.12, found *m/z* 340.25.

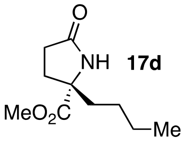

17d

Methyl 2-butyl-5-oxopyrrolidine-2-carboxylate (17d): Prepared as described in the general procedure with a 20% catalyst loading and 48 h reaction time. The title compound was isolated as a clear oil in 54% yield. ^1H NMR (400 MHz, $CDCl_3$): 6.12 (br s, 1H, N*H*), 3.78 (s, 3H, CO_2CH_3), 2.55-2.45 (m, 1H, alkyl*H*), 2.42-2.36 (m, 2H, alkyl*H*), 2.15-2.05 (m, 1H, alkyl*H*), 1.91-1.83 (m, 1H, alkyl*H*), 1.79-1.69 (m, 1H, alkyl*H*), 1.40-1.15 (m, 4H, alkyl*H*) 0.92 (t, *J* = 7.6 Hz, 3H, CH_3).

^{13}C NMR (101 MHz, $CDCl_3$) δ 176.68, 174.03, 65.60, 52.60, 39.14, 30.65, 29.65, 26.23, 22.62, 13.80. LRMS (APCI+): exact mass calc'd for $C_{10}H_{17}NO_3$ $[M+1]^+$ requires *m/z* 200.12, found *m/z* 200.40. Enantiometric excess (82% ee) was determined by the addition of chiral shift reagent.

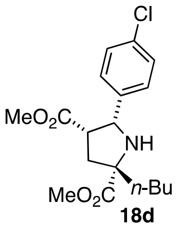

18d

Dimethyl 2-butyl-5-(4-chlorophenyl)pyrrolidine-2,4-dicarboxylate (18d): Prepared as described in the general procedure with a 20% catalyst loading and 48 h reaction time. The title compound was isolated as a white solid in 11% yield. ^{1}H NMR (400 MHz, $CDCl_3$): 7.25 (q, J = 8.7 Hz, 4H, Ar*H*), 4.56 (d, J =7.2 Hz, 1H, ArC*H*), 3.82 (s, 3H, CO_2CH_3), 3.25 (s, 3H, CO_2CH_3), 3.30-3.23 (m, 1H, $MeCO_2CH$), 3.03 (br s, 1H, N*H*), 2.70 (dd, J = 13.7 Hz, 4.5 Hz, 1H, CH_2), 2.06 (dd, J = 13.8 Hz, 7.5 Hz, 1H, CH_2), 1.89-1.80 (m, 1H, alkyl*H*), 1.67-1.56 (m, 1H, alkyl*H*), 1.50-1.38 (m, 1H, alkyl*H*), 1.35-1.25 (m, 2H, alkyl*H*), 1.15-1.03 (m, 1H, alkyl*H*), 0.89 (t, J= 7.4Hz, 3H, CH_2CH_3). ^{13}C NMR (101 MHz, $CDCl_3$) δ 176.27, 173.00, 137.87, 133.29, 128.36, 128.13, 69.52, 64.75, 52.41, 51.30, 50.23, 40.81, 39.65, 27.29, 22.89, 13.98. LRMS (APCI+): exact mass calc'd for $C_{18}H_{24}ClNO_4$ $[M+1]^+$ requires *m/z* 354.13, found *m/z* 354.19.

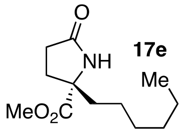

17e

Methyl 2-hexyl-5-oxopyrrolidine-2-carboxylate (17e): Prepared as described in the general procedure with a 20% catalyst loading and 48 h reaction time. The title compound was isolated as a clear oil in 46% yield. ^{1}H NMR (400 MHz, $CDCl_3$): 6.01 (br s, 1H, N*H*), 3.76 (s, 3H, CO_2CH_3), 2.53-2.43 (m, 1H, alkyl*H*), 2.40-2.35 (m, 2H, alkyl*H*), 2.12-2.02 (m, 1H, alkyl*H*), 1.89-1.80 (m, 1H, alkyl*H*), 1.76-1.68 (m, 1H, alkyl*H*), 1.35-1.16 (m, 8H, alkyl*H*) 0.88 (t, J = 7.6 Hz, 3H, CH_3).

^{13}C NMR (101 MHz, $CDCl_3$) δ 176.62, 174.02, 65.60, 52.60, 39.42, 31.48, 30.66, 29.63, 29.14, 24.06, 22.46, 13.96. LRMS (APCI+): exact mass calc'd for $C_{12}H_{21}NO_3$ $[M+1]^+$ requires *m/z* 228.15, found *m/z* 228.44. Enantiometric excess (80% ee) was determined by the addition of chiral shift reagent.

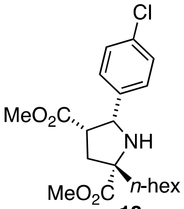

18e

Dimethyl 2-hexyl-5-(4-chlorophenyl)pyrrolidine-2,4-dicarboxylate (18e): Prepared as described in the general procedure with a 20% catalyst loading and 48 h reaction time. The title compound was isolated as a white solid in 9% yield. ^1H NMR (400 MHz, $CDCl_3$): 7.25 (q, *J* = 8.7 Hz, 4H, Ar*H*), 4.56 (d, *J* =7.2 Hz, 1H, ArC*H*), 3.82 (s, 3H, CO_2CH_3), 3.25 (s, 3H, CO_2CH_3), 3.30-3.23 (m, 1H, $MeCO_2CH$), 3.00 (br s, 1H, N*H*), 2.70 (dd, *J* = 13.7 Hz, 4.5 Hz, 1H, CH_2), 2.06 (dd, *J* = 13.8 Hz, 7.5 Hz, 1H, CH_2), 1.94-1.79 (m, 1H, alkyl*H*), 1.67-1.57 (m, 1H, alkyl*H*), 1.50-1.35 (m, 1H, alkyl*H*), 1.35-1.15 (m, 6H, alkyl*H*), 1.15-1.03 (m, 1H, alkyl*H*), 0.87 (t, *J*= 7.4Hz, 3H, CH_2CH_3). ^{13}C NMR (101 MHz, $CDCl_3$) δ 176.24, 172.96, 137.95, 133.27, 128.35, 128.14, 69.53, 64.72, 52.37, 51.27, 50.21, 41.05, 39.60, 31.69, 29.44, 25.07, 22.57, 14.02. LRMS (APCI+): exact mass calc'd for $C_{20}H_{28}ClNO_4$ $[M+1]^+$ requires *m/z* 382.17, found *m/z* 382.34.

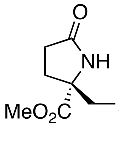

17f

Methyl 2-isobutyl-5-oxopyrrolidine-2-carboxylate (17f): Prepared as described in the general procedure with a 20% catalyst loading and 48 h reaction time. The title compound was isolated as a white solid in 62% yield. ^1H NMR (400 MHz, $CDCl_3$): 6.15 (br s, 1H, N*H*), 3.78 (s, 3H, CO_2CH_3), 2.56-2.46 (m, 1H, alkyl*H*), 2.40-2.34 (m, 2H, alkyl*H*), 2.13-2.04 (m, 1H, alkyl*H*), 1.91-

1.84 (m, 1H, alkyl*H*), 1.73-1.61 (m, 2H, alkyl*H*), 0.95 (d, *J* = 6.4 Hz, 3H, CH_3), 0.90 (d, *J* = 6.4Hz, 3H, CH_3). ^{13}C NMR (101 MHz, $CDCl_3$) δ 176.74, 174.33, 65.25, 52.57, 48.07, 32.24, 29.40, 24.90, 23.82, 23.00. LRMS (APCI+): exact mass calc'd for $C_{10}H_{17}NO_3$ $[M+1]^+$ requires *m/z* 200.12, found *m/z* 200.18. HPLC analysis: Chiralpak OD-H (Hex/iPrOH = 97/3, 1 mL/min, 210 nm), 25.5 min (major), 29.8 min (minor), 77% ee.

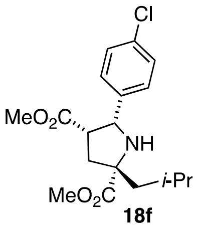

18f

Dimethyl 2-isobutyl-5-(4-chlorophenyl)pyrrolidine-2,4-dicarboxylate (18f): Prepared as described in the general procedure with a 20% catalyst loading and 48 h reaction time. The title compound was isolated as a clear oil in 10% yield. ^{1}H NMR (400 MHz, $CDCl_3$): 7.29-7.22 (m, 4H, Ar*H*), 4.56 (d, *J* = 7.6 Hz, 1H, ArC*H*), 3.81 (s, 3H, CO_2CH_3), 3.34 – 3.26 (m, 1H, $MeCO_2CH$), 3.24 (s, 3H, CO_2CH_3), 2.97 (br s, 1H, N*H*), 2.64 (dd, *J* = 13.7, 5.2 Hz, 1H, alkyl*H*), 2.04 (dd, *J* = 13.6, 7.6 Hz, 1H, alkyl*H*), 1.85 – 1.69 (m, 2H, alkyl*H*), 1.59 (dd, *J* = 13.2, 4.9 Hz, 1H, alkyl*H*), 0.95 (d, *J* = 6.5 Hz, 3H, CH_3), 0.84 (d, *J* = 6.4 Hz, 3H, CH_3). ^{13}C NMR (101 MHz, $CDCl_3$) δ 176.54, 172.98, 138.13, 133.24, 128.32, 128.23, 68.99, 64.53, 52.27, 51.30, 49.94, 48.94, 40.90, 25.28, 24.29, 22.85. LRMS (APCI+): exact mass calc'd for $C_{18}H_{24}ClNO_4$ $[M+1]^+$ requires *m/z* 354.14, found *m/z* 354.19.

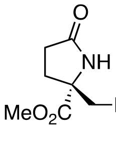

17g

Methyl 2-benzyl-5-oxopyrrolidine-2-carboxylate (17g): Prepared as described in the general procedure with a 15% catalyst loading and 48 h reaction time. The title compound was isolated as a white solid in a 77% yield. ^{1}H NMR (400 MHz, $CDCl_3$): 7.36-7.28 (m, 3H, Ar*H*), 7.17-7.13 (m,

2H, Ar*H*), 5.89 (br s, 1H, N*H*), 3.74 (s, 3H, CO_2CH_3), 3.30 (d, *J* = 13.6 Hz, 1H, $PhCH_2$), 2.93 (d, *J* = 13.6 Hz, 1H, $PhCH_2$), 2.54-2.45 (m, 1H, alkyl*H*), 2.39-2.15 (m, 3H, alkyl*H*). ^{13}C NMR (101 MHz, $CDCl_3$) δ 176.40, 173.56, 134.78, 129.66, 128.77, 127.57, 66.37, 52.60, 45.21, 30.91, 29.60. LRMS (APCI+): exact mass calc'd for $C_{13}H_{15}NO_3$ $[M+1]^+$ requires *m/z* 234.11, found *m/z* 233.97. HPLC analysis: Chiralpak AD-H (Hex/iPrOH = 90/10, 1 mL/min, 210 nm), 11.3 min (major), 21.5 min (minor), 75% ee.

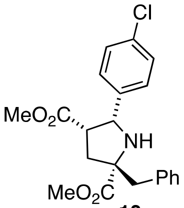

18g

Dimethyl 2-benzyl-5-(4-chlorophenyl)pyrrolidine-2,4-dicarboxylate (18g): Prepared as described in the general procedure with a 15% catalyst loading and 48 h reaction time. The title compound was isolated as a white solid in 21% yield. ^1H NMR (400 MHz, $CDCl_3$): 7.30-7.18 (m, 9H, Ar*H*), 4.49 (d, *J* =7.6 Hz, 1H, ArC*H*), 3.75 (s, 3H, CO_2CH_3), 3.23 (s, 3H, CO_2CH_3), 3.19-3.15 (m, 1H, $MeCO_2CH$), 3.12 (d, *J* = 13.2 Hz, 1H, $PhCH_2$), 2.93 (d, *J* = 13.2 Hz, 1H, $PhCH_2$), 2.84 (br s, 1H, N*H*), 2.74 (dd, *J* = 14.0 Hz, 5.6 Hz, 1H, CH_2), 2.20 (dd, *J* = 14.0 Hz, 7.6 Hz, 1H, CH_2). ^{13}C NMR (101 MHz, $CDCl_3$) δ 175.60, 172.69, 138.43, 136.75, 133.25, 130.09, 128.27, 128.16, 126.89, 70.28, 64.26, 52.29, 51.30, 49.80, 45.82, 38.28. LRMS (APCI+): exact mass calc'd for $C_{21}H_{22}ClNO_4$ $[M+1]^+$ requires *m/z* 388.12, found *m/z* 388.33.

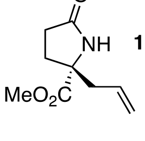

17i

Methyl 2-allyl-5-oxopyrrolidine-2-carboxylate (17i): Prepared as described in the general procedure with a 10% catalyst loading and 48 h reaction time. The title compound was isolated as

a white solid in 69% yield. ^{1}H NMR (400 MHz, $CDCl_3$): ^{1}H NMR (400 MHz, $CDCl_3$): 5.97 (br s, 1H, N*H*), 5.73-5.61 (m, 1H, C=C*H*), 5.22-5.13 (m, 2H, C=C*H*), 3.76 (s, 3H, CO_2CH_3), 2.70-2.62 (m, 2H, alkyl*H*), 2.50-2.35 (m, 4H, alkyl*H*) 2.16-2.08 (m, 1H, alkyl*H*). ^{13}C NMR (101 MHz, $CDCl_3$) δ 176.57, 173.56, 130.96, 120.68, 65.13, 52.70, 43.53, 30.26, 29.66. LRMS (APCI+): exact mass calc'd for $C_9H_{13}NO_3$ $[M+1]^+$ requires *m/z* 184.09, found *m/z* 184.15. Enantiometric excess (94% ee) was determined by the addition of chiral shift reagent.

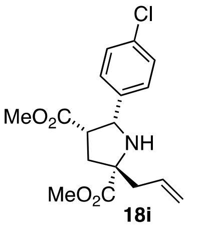

18i

Dimethyl 2-allyl-5-(4-chlorophenyl)pyrrolidine-2,4-dicarboxylate (18i): Prepared as described in the general procedure with a 10% catalyst loading and 48 h reaction time. The title compound was isolated as a white solid in 23% yield. ^{1}H NMR (400 MHz, $CDCl_3$): 7.32-7.25 (m, 4H, Ar*H*), 5.92-5.79 (m, 1H, C*H*=CH_2), 5.17 (s, 1H, CH=CH_2), 5.15 (d, *J* = 3.6 Hz, 1H, CH=CH_2), 4.62 (d, *J* =7.6 Hz, 1H, ArC*H*), 3.84 (s, 3H, CO_2CH_3), 3.36-3.28 (m, 1H, MeCO_2C*H*), 3.28 (s, 3H, CO_2CH_3), 3.00 (br s, 1H, N*H*), 2.74 (dd, *J* = 13.6 Hz, 4.8 Hz, 1H, CH_2), 2.60 (dd, *J* = 13.6, 7.6 Hz, 1H, CH_2CH=CH_2), 2.45 (dd, *J* = 13.6, 7.6 Hz, 1H, CH_2CH=CH_2), 2.14 (dd, *J* = 13.6 Hz, 7.6 Hz, 1H, CH_2). ^{13}C NMR (101 MHz, $CDCl_3$) δ 175.68, 172.76, 138.02, 133.50, 133.30, 128.34, 128.18, 118.70, 69.31, 64.52, 52.44, 51.32, 50.09, 44.89, 38.34. LRMS (APCI+): exact mass calc'd for $C_{17}H_{20}ClNO_4$ $[M+1]^+$ requires *m/z* 338.11, found *m/z* 338.27.

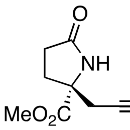

17j

Methyl 5-oxo-2-(prop-2-yn-1-yl)pyrrolidine-2-carboxylate (17j): Prepared as described in the general procedure with a 10% catalyst loading and 16 h reaction time. The title compound was

isolated as a white solid in 64% yield. ^{1}H NMR (400 MHz, $CDCl_3$): 5.99 (br s, 1H, N*H*), 3.80 (s, 3H, CO_2CH_3), 2.82 (dd, *J* = 16.6, 2.6 Hz, 1H, C*H*$_2$≡CH), 2.60 (dd, *J* = 16.6, 2.6 Hz, 1H, C*H*$_2$≡CH), 2.55 – 2.36 (m, 3H, alkyl*H*), 2.29 – 2.13 (m, 1H, alkyl*H*), 2.08 (t, *J* = 2.6 Hz, 1H, CH_2≡C*H*). ^{13}C NMR (101 MHz, $CDCl_3$) δ 176.41, 172.67, 77.84, 72.20, 64.48, 53.07, 30.22, 29.68, 29.43. LRMS (APCI+): exact mass calc'd for $C_9H_{11}NO_3$ $[M+1]^+$ requires *m/z* 182.07, found *m/z* 182.39. Enantiometric excess (88% ee) was determined by the addition of chiral shift reagent.

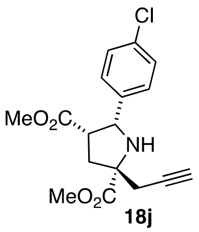

18j

Dimethyl 5-(4-chlorophenyl)-2-(prop-2-yn-1-yl)pyrrolidine-2,4-dicarboxylate (18j): Prepared as described in the general procedure with a 10% catalyst loading and 16 h reaction time. The title compound was isolated as a white solid in 20% yield. ^{1}H NMR (400 MHz, $CDCl_3$) 7.37 – 7.19 (m, 4H, Ar*H*), 4.74 (d, *J* = 7.5 Hz, 1H, ArC*H*), 3.86 (s, 3H, CO_2CH_3), 3.42-3.33 (m, 1H, $MeCO_2CH$), 3.25 (s, 3H, CO_2CH_3), 3.11 (br s, 1H, N*H*), 2.84 – 2.68 (m, 2H, C*H*$_2$≡CH and C*H*$_2$), 2.63 (dd, *J* = 16.4, 2.6 Hz, 1H, C*H*$_2$≡CH), 2.24 (dd, *J* = 13.8, 7.5 Hz, 1H, C*H*$_2$), 2.06 (t, *J* = 2.6 Hz, 1H, CH_2≡C*H*). ^{13}C NMR (101 MHz, $CDCl_3$) δ 174.73, 172.48, 138.03, 133.40, 128.35, 128.24, 79.95, 71.00, 68.64, 64.77, 52.85, 51.39, 50.13, 37.80, 30.71. LRMS (APCI+): exact mass calc'd for $C_{20}H_{28}ClNO_4$ $[M+1]^+$ requires *m/z* 336.09, found *m/z* 336.01.

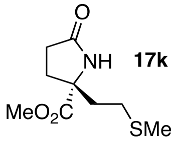

17k

Methyl 2-methylthioethyl-5-oxopyrrolidine-2-carboxylate (17k): Prepared as described in the general procedure with a 10% catalyst loading and 16 h reaction time. The title compound was

isolated as a white solid in 76% yield. ^1H NMR (400 MHz, $CDCl_3$): ^1H NMR (400 MHz, $CDCl_3$): 6.51 (br s, 1H, N*H*), 3.78 (s, 3H, CO_2CH_3), 2.53-2.42 (m, 3H, alkyl*H*), 2.40-2.34 (m, 2H, alkyl*H*), 2.27-2.17 (m, 1H, alkyl*H*), 2.14-2.06 (m, 1H, alkyl*H*), 2.10 (s, 3H, SCH_3) 2.06-1.97 (m, 1H, alkyl*H*). ^{13}C NMR (101 MHz, $CDCl_3$) δ 176.84, 173.58, 65.24, 52.85, 38.26, 31.27, 29.36, 28.95, 15.58. LRMS (APCI+): exact mass calc'd for $C_9H_{15}NO_3S$ $[M+1]^+$ requires *m/z* 218.08, found *m/z* 218.18. Enantiometric excess (84% ee) was determined by the addition of chiral shift reagent.

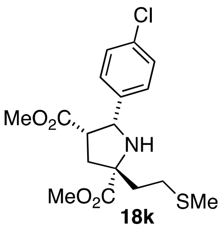

18k

Dimethyl 2-methylthioethyl-5-(4-chlorophenyl)pyrrolidine-2,4-dicarboxylate (18k): Prepared as described in the general procedure with a 10% catalyst loading and 16 h reaction time. The title compound was isolated as a white solid in 19% yield. ^1H NMR (400 MHz, $CDCl_3$): 7.31-7.19 (m, 4H, Ar*H*), 4.54 (d, *J* =7.2 Hz, 1H, ArC*H*), 3.83 (s, 3H, CO_2CH_3), 3.26 (s, 3H, CO_2CH_3), 3.35-3.25 (m, 1H, $MeCO_2CH$), 3.09 (br s, 1H, N*H*), 2.75-2.60 (m, 2H, CH_2), 2.40-2.30 (m, 1H, CH_2), 2.21-2.03 (m, 3H, CH_2), 2.10 (s, 3H, SCH_3), 1.93-1.84 (m, 2H, CH_2). ^{13}C NMR (101 MHz, $CDCl_3$) δ 175.56, 172.90, 137.54, 133.42, 128.44, 128.08, 69.17, 64.84, 52.64, 51.36, 50.19, 40.12, 29.71, 15.69. LRMS (APCI+): exact mass calc'd for $C_{17}H_{22}ClNO_4S$ $[M+1]^+$ requires *m/z* 372.10, found *m/z* 372.23.

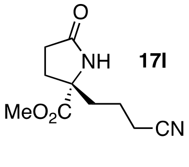

17l

Methyl 2-(3-cyanopropyl)-5-oxopyrrolidine-2-carboxylate (17l): Prepared as described in the general procedure with a 10% catalyst loading and a 16 h reaction time. The title compound was

isolated as a white solid in 75% yield. ^{1}H NMR (400 MHz, $CDCl_3$): ^{1}H NMR (400 MHz, $CDCl_3$): 5.96 (br s, 1H, N*H*), 3.79 (s, 3H, CO_2CH_3), 2.57-2.47 (m, 1H, alkyl*H*), 2.43-2.37 (m, 4H, alkyl*H*), 2.13-1.87 (m, 3H, alkyl*H*), 1.72-1.58 (m, 2H, alkyl*H*). ^{13}C NMR (101 MHz, $CDCl_3$) δ 176.61, 173.28, 118.60, 64.79, 53.00, 38.03, 30.52, 29.47, 20.37, 17.22. LRMS (APCI+): exact mass calc'd for $C_{18}H_{21}ClN_2O_4$ $[M+1]^+$ requires *m/z* 211.10, found *m/z* 211.14. Enantiometric excess (16% ee) was determined by the addition of chiral shift reagent.

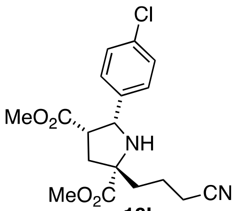

18l

Dimethyl 5-(4-chlorophenyl)-2-(3-cyanopropyl)pyrrolidine-2,4-dicarboxylate (18l): Prepared as described in the general procedure with a 10% catalyst loading and a 16 h reaction time. The title compound was isolated as a white solid in 16% yield (9:1 mixture of 2 isomers). ^{1}H NMR (400 MHz, $CDCl_3$) (major isomer): 7.32-7.25 (m, 4H, Ar*H*), 4.62 (d, *J* = 8.8 Hz, 1H, ArC*H*), 3.81 (s, 3H, CO_2CH_3), 3.25 (q, 1H, *J* = 8.0 Hz, $MeCO_2CH$), 3.19 (s, 3H, CO_2CH_3), 2.85 (br s, 1H, N*H*), 2.55-2.32 (m, 4H, alkyl), 2.15-2.02 (m, 2H, alkyl), 1.90-1.80 (m, 1H, alkyl), 1.79-1.65 (m, 1H,alkyl). ^{13}C NMR (101 MHz, $CDCl_3$) δ 176.79, 172.47, 138.92, 133.22, 128.68, 128.04, 119.27, 68.37, 62.83, 52.70, 51.34, 48.48, 37.88, 36.21, 21.23, 17.47. LRMS (APCI+): exact mass calc'd for $C_{18}H_{21}ClN_2O_4$ $[M+1]^+$ requires *m/z* 365.12, found *m/z* 365.42.

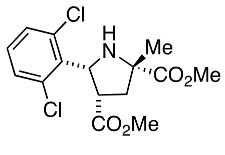

Dimethyl 2-methyl-5-(2,6-dichlorophenyl)pyrrolidine-2,4-dicarboxylate: Prepared as described in the general procedure with a 10% catalyst loading and 48 h reaction time. The title

compound was isolated as a white solid in a 69% yield. ^{1}H NMR (400 MHz, $CDCl_3$): 7.30 (d, *J* = 8.0 Hz, 2H, Ar*H*), 7.13 (t, *J* = 8.0 Hz, 1H, Ar*H*), 5.22 (d, *J* = 8.0 Hz, 1H, ArC*H*), 4.53 (br s, 1H, N*H*), 3.84 (s, 3H, CO_2CH_3), 3.50-3.41 (m, 1H, $MeCO_2CH$), 3.38 (s, 3H, CO_2CH_3), 2.82 (dd, *J* = 13.6 Hz, 5.2 Hz, 1H, C*H*2), 2.05 (dd, *J* = 13.6 Hz, 7.6 Hz, 1 H, C*H*2). ^{13}C NMR (101 MHz, $CDCl_3$) δ 175.90, 172.64, 135.55, 132.49, 129.40, 128.91, 65.92, 62.09, 52.58, 51.80, 48.24, 40.47, 26.13. LRMS (APCI+): exact mass calc'd for $C_{15}H_{17}Cl_2NO_4$ $[M+1]^+$ requires *m/z* 346.05, found *m/z* 346.18. HPLC analysis: Chiralpak AD-H (Hex/iPrOH = 90/10, 1 mL/min, 254 nm), 6.7 min, 7.4 min, 0% ee.

Synthesis of imines

Method A

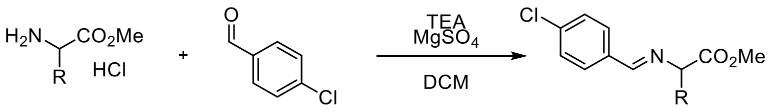

According to the method of Wang et. al.,[1] the racemic amino acid methyl ester hydrogen chloride (6 mmol, 1.2 equiv) and magnesium sulfate (960 mg, 8 mmol, 1.6 equiv) were dissolved in DCM (10 mL) and triethylamine (1 mL). After stirring for one hour at room temperature, the aldehyde (5 mmol, 1 equiv) was added and the mixture was stirred overnight. The mixture was filtered and washed twice with water and with brine. The organic layer was dried with sodium sulfate and concentrated to furnish the product which was used without further purification.

Method B

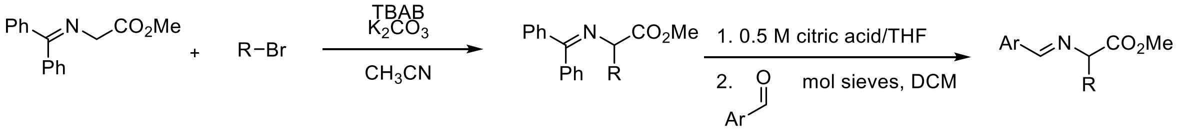

[1] Wang, C. J.; Liang, G.; Xue, Z. Y.; Gao, F. *J. Am. Chem. Soc.* **2008**, *130*, 17250.

Glycine benzophenone imine (2.02 g, 8 mmol, 1 equiv), tetrabutylammonium bromide (258 mg, 0.8 mmol, 0.1 equiv), and potassium carbonate (1.1 g, 8 mmol, 1 equiv) were dissolved in acetonitrile (20 mL). The mixture was stirred for 15 minutes at room temperature at which point the alkyl bromide (9.6 mmol, 1.2 equiv) was added. The mixture was heated to reflux and stirred overnight at reflux. Upon cooling it was filtered, concentrated and purified by silica gel chromatography.

The benzophenone imine (3 mmol) was dissolved in THF (30 mL) and 0.5 M citric acid (30 mL). After stirring for three hours the THF was removed by rotary evaporation and the remaining aqueous layer was extracted five times with ether. The aqueous layer was basified with saturated sodium bicarbonate and extracted three times with DCM. The combined organic layers were dried and concentrated to furnish the free amine, which was dissolved in DCM (0.25 M) with molecular sieves. The aldehyde (1 equiv, based on the mass of the free amine) was added and the mixture was stirred at room temperature overnight. The mixture was filtered, dried with sodium sulfate, and concentrated to furnish the imine, which was used without further purification.

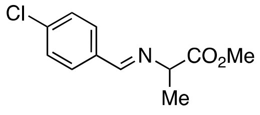

1

Methyl (*E*)-2-[(4-chloro-benzylidene)amino]propanoate (1): Synthesized according to method A to furnish the title compound as a pale yellow oil in 91% yield. Spectral data were consistent with those previously reported.[2]

[2] Achard, T.; Belokon, Y.N.; Fuentes, J. A.; North, M.; Parsons, T. *Tetrahedron* **2004,** *60,* 5919.

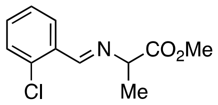

12

Methyl (*E*)-2-[(2-chlorobenzylidene)amino]propanoate (12): Synthesized according to method A to furnish the title compound as a pale yellow oil in 71% yield. Spectral data were consistent with those previously reported.[2]

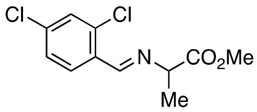

13

Methyl (*E*)-2-[(2,4-dichlorobenzylidene)amino]propanoate (13): Synthesized according to method A to furnish the title compound as a pale yellow oil in 70% yield. ^{1}H NMR (400 MHz, $CDCl_3$): 8.71 (s, 1H, N=C*H*), 8.09 (d, *J* = 8.8 Hz, 1H, Ar*H*), 7.5-7.25 (m, 2H, Ar*H*), 4.25 (q, *J* = 6.8 Hz, 1H, C*H*CH_3), 3.79 (s, 3H, CO_2CH_3), 1.56 (d, *J* = 6.8 Hz, 3 H, $CHCH_3$). ^{13}C NMR (101 MHz, $CDCl_3$) δ 172.42, 158.39, 137.30, 135.77, 131.33, 129.54, 129.38, 127.45, 67.83, 52.15, 19.36. LRMS (APCI+): exact mass calc'd for $C_{11}H_{11}Cl_2NO_2$ $[M+1]^+$ requires *m/z* 260.02, found *m/z* 260.25.

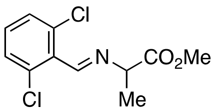

14

Methyl (*E*)-2-[(2,6-dichlorobenzylidene)amino]propanoate (14): Synthesized according to method A to furnish the title compound as a white solid in 61% yield. ^{1}H NMR (400 MHz, $CDCl_3$): 8.47 (s, 1H, N=C*H*), 7.34 (d, *J* = 7.6 Hz, 2H, Ar*H*), 7.23 (t, *J* = 7.6 Hz, 1H, Ar*H*), 4.27 (q, *J* = 6.8 Hz, 1H, C*H*CH_3), 3.78 (s, 3H, CO_2CH_3), 1.59 (d, *J* = 6.8 Hz, 3 H, $CHCH_3$). ^{13}C NMR (101 MHz, $CDCl_3$) δ 172.29, 158.85, 134.66, 132.84, 130.52, 128.54, 68.51, 52.30, 19.34. LRMS (APCI+): exact mass calc'd for $C_{11}H_{11}Cl_2NO_2$ $[M+1]^+$ requires *m/z* 260.02, found *m/z* 260.10.

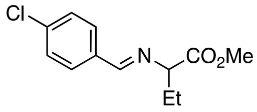

16b

Methyl (*E*)-2-[(4-chlorobenzylidene)amino]butanoate (16b): Synthesized according to method A to furnish the title compound as a pale yellow oil in 90% yield. Spectral data were consistent with those previously reported.[3]

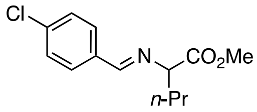

16c

Methyl (*E*)-2-[(4-chlorobenzylidene)amino]pentanoate (17c): Synthesized according to method A to furnish the title compound as a yellow oil in 88% yield. ^{1}H NMR (400 MHz, $CDCl_3$): 8.23 (s, 1H, N=C*H*), 7.72 (d, *J* = 8.4 Hz, 2H, Ar*H*), 7.39 (d, *J* = 8.4 Hz, 2H, Ar*H*), 3.99 (dd, *J* = 8.0 Hz, 4.8 Hz, 1H, C*H*Pr), 3.74 (s, 3H, CO_2CH_3), 2.04-1.81 (m, 1H, $CH_2CH_2CH_3$), 1.31-1.17 (m, 1H, CH_2CH_3), 1.93 (t, *J* = 7.2 Hz, CH_2CH_3). ^{13}C NMR (101 MHz, $CDCl_3$) δ 172.31, 161.61, 136.88, 134.17, 129.63, 128.72, 72.93, 51.90, 35.34, 19.04, 13.60. LRMS (APCI+): exact mass calc'd for $C_{13}H_{16}ClNO_2$ $[M+1]^+$ requires *m/z* 254.09, found *m/z* 254.18.

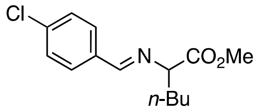

16d

Methyl (*E*)-2-[(4-chlorobenzylidene)amino]hexanoate (16d): Synthesized according to method A to furnish the title compound as a brown oil in 85% yield. ^{1}H NMR (400 MHz, $CDCl_3$): 8.25 (s, 1H, N=C*H*), 7.74 (d, *J* = 8.4 Hz, 2H, Ar*H*), 7.42 (d, *J* = 8.4 Hz, 2H, Ar*H*), 3.99 (dd, *J* = 8.3, 5.4 Hz, 1H), 3.77 (s, 3H, CO_2CH_3), 2.13 – 1.78 (m, 2H, alkyl*H*), 1.45 – 1.12 (m, 4H, alkyl*H*), 0.92 (t, *J* = 7.1 Hz, 3H, CH_2CH_3). ^{13}C NMR (101 MHz, $CDCl_3$) δ 172.58, 161.72, 137.09, 134.17, 129.72,

[3] Belokon, Y. N.; Bhave, D.; D'addario, D.; Groaz, E.; North, M.; Tagliazucca, V. *Tetrahedron* **2004**, *60*, 1849.

128.86, 73.46, 52.12, 33.12, 28.07, 22.33, 13.91. LRMS (APCI+): exact mass calc'd for $C_{14}H_{18}ClNO_2$ $[M+1]^+$ requires *m/z* 268.10, found *m/z* 268.11.

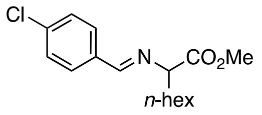

16e

Methyl (*E*)-2-[(4-chlorobenzylidene)amino]octanoate (16e): Synthesized according to method A to furnish the title compound as a brown oil in 82% yield. ^{1}H NMR (400 MHz, $CDCl_3$): . ^{1}H NMR (400 MHz, $CDCl_3$): 8.23 (s, 1H, N=C*H*), 7.72 (d, *J* = 8.4 Hz, 2H, Ar*H*), 7.39 (d, *J* = 8.4 Hz, 2H, Ar*H*), 3.97 (dd, *J* = 8.3, 5.4 Hz, 1H), 3.74 (s, 3H, CO_2CH_3), 2.08 – 1.81 (m, 2H, alkyl*H*), 1.35 – 1.18 (m, 8H, alkyl*H*), 0.86 (t, *J* = 6.7 Hz, 3H, CH_2CH_3). ^{13}C NMR (101 MHz, $CDCl_3$) δ 172.56, 161.70, 137.07, 134.17, 129.70, 128.85, 73.44, 52.09, 33.39, 31.59, 28.87, 25.84, 22.53, 14.01. LRMS (APCI+): exact mass calc'd for $C_{16}H_{22}ClNO_2$ $[M+1]^+$ requires *m/z* 296.13, found *m/z* 296.18.

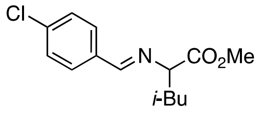

16f

Methyl (*E*)-2-[(4-chlorobenzylidene)amino]-4-methylpentanoate (16f): Synthesized according to method A to furnish the title compound as a pale yellow oil in 84% yield. Spectral data were consistent with those previously reported.[3]

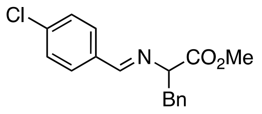

16g

Methyl (*E*)-2-[(4-chlorobenzylidene)amino]-3-phenylpropanoate (16g): Synthesized according to method A to furnish the title compound as a white solid in 86% yield. Spectral data were consistent with those previously reported.[3]

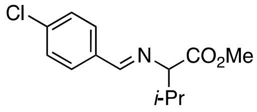

16h

Methyl (*E*)-2-[(4-chlorobenzylidene)amino]-3-methylbutanoate (16h): Synthesized according to method A to furnish the title compound as a pale yellow oil in 75% yield. Spectral data were consistent with those previously reported.[3]

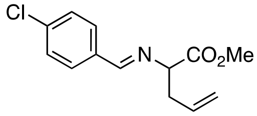

16i

Methyl (*E*)-2-[(4-chlorobenzylidene)amino]but-3-enoate (16i): Synthesized according to method B to furnish the title compound as a pale yellow oil in 53% yield from the allyl benzophenone glycine imine. Spectral data were consistent with those previously reported.[9]

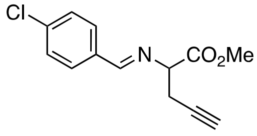

16j

Methyl (*E)*-2-((4-chlorobenzylidene)amino)pent-4-ynoate (16j): Synthesized according to method B to furnish the title compound as a pale yellow oil in 23% yield from the propargyl benzophenone glycine imine. ^{1}H NMR (400 MHz, $CDCl_3$): 8.31 (s, 1H, N=C*H*), 7.73 (d, *J* = 8.5 Hz, 2H, Ar*H*), 7.40 (d, *J* = 8.5 Hz, 2H, Ar*H*), 4.12 (dd, *J* = 8.5, 5.2 Hz, 1H, C*H*CO_2CH_3), 3.77 (s, 3H, CO_2CH_3), 2.94 (ddd, *J* = 16.8, 5.2, 2.6 Hz, 1H, CH_2≡CH), 2.75 (ddd, *J* = 16.8, 8.5, 2.6 Hz, 1H, CH_2≡CH), 2.00 (t, *J* = 2.6 Hz, 1H, CH_2≡C*H*). ^{13}C NMR (101 MHz, $CDCl_3$) δ 170.73, 163.36, 137.43, 133.87, 129.91, 128.92, 80.09, 71.57, 71.02, 52.54, 23.30. LRMS (APCI+): exact mass calc'd for $C_{13}H_{12}ClNO_2$ $[M+1]^+$ requires *m/z* 250.06, found *m/z* 250.37.

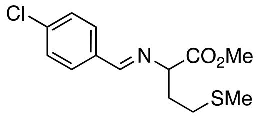

16k

Methyl (*E*)-2-[(4-chlorobenzylidene)amino]-4-(methylthio)butanoate (16k): Synthesized according to method A to furnish the title compound as an orange oil in 90% yield. ^{1}H NMR (400 MHz, $CDCl_3$): 8.28 (s, 1H, N=C*H*), 7.72 (d, *J* = 8.4 Hz, 2H, Ar*H*), 7.40 (d, *J* = 8.4 Hz, 2H, Ar*H*), 4.21 (dd, *J* = 8.4 Hz, 5.2 Hz, 1H, C*H*CO_2CH_3), 3.75 (s, 3H, CO_2C*H_3*), 2.64-2.54 (m, 1H, CHC*H_2*), 2.50-2.39 (m, 1H, CHC*H_2*), 2.33-2.17 (m, 2H, C*H_2*S), 2.09 (s, 3H, SC*H_3*). ^{13}C NMR (101 MHz, $CDCl_3$) δ 171.82, 162.85, 137.06, 134.01, 129.71, 128.79, 70.93, 52.15, 31.90, 30.14, 15.08. LRMS (APCI+): exact mass calc'd for $C_{13}H_{16}ClNO_2S$ $[M+1]^+$ requires *m/z* 286.06, found *m/z* 286.07.

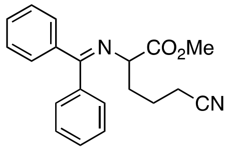

Methyl 5-cyano-2-[(diphenylmethylene)amino]pentanoate: Synthesized according to method B to furnish the title compound as a white solid in 43% yield. ^{1}H NMR (400 MHz, $CDCl_3$): 7.64 (d, *J* = 7.2, 2H, Ar*H*), 7.51-7.76 (m, 4H, Ar*H*), 7.34 (t, *J* = 8.2 Hz, 2H, Ar*H*), 7.21 – 7.13 (m, 2H, Ar*H*), 4.10 (dd, *J* = 7.4, 5.1 Hz, 1H, C*H*CO_2CH_3), 3.72 (s, 3H, CO_2C*H_3*), 2.37 – 2.22 (m, 2H, alkyl*H*), 2.12 – 1.96 (m, 2H, alkyl*H*), 1.67 (m, 2H, alkyl*H*). ^{13}C NMR (101 MHz, $CDCl_3$) δ 172.07, 171.20, 139.14, 136.07, 130.62, 128.88, 128.83, 128.72, 128.15, 127.68, 119.37, 64.25, 52.26, 32.48, 22.10, 17.08. LRMS (APCI+): exact mass calc'd for $C_{20}H_{20}N_2O_2$ $[M+1]^+$ requires *m/z* 321.15, found *m/z* 321.11.

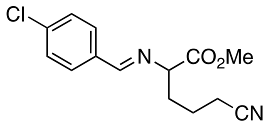

16l

Methyl (*E*)-2-[(4-chlorobenzylidene)amino]-5-cyanopentanoate (16l): Synthesized according to method B to furnish the title compound as a pale yellow oil in 45% yield. ^{1}H NMR (400 MHz, $CDCl_3$): 8.27 (s, 1H, N=C*H*), 7.72 (d, *J* = 8.4 Hz, 2H, Ar*H*), 7.41 (d, *J* = 8.4 Hz, 2H, Ar*H*), 4.03 (dd, *J* = 7.4, 5.6 Hz, 1H, C*H*CO_2Me), 3.76 (s, 3H, CO_2Me), 2.41 (t, *J* = 7.1 Hz, 2H, CH_2CN), 2.18 – 2.01 (m, 2H, alkyl*H*), 1.73 (m, 2H, alkyl*H*). ^{13}C NMR (101 MHz, $CDCl_3$) δ 171.53, 162.85, 137.47, 133.84, 129.78, 128.98, 119.25, 71.98, 52.39, 32.19, 22.01, 17.09. LRMS (APCI+): exact mass calc'd for $C_{14}H_{15}ClN_2O_2$ $[M+1]^+$ requires *m/z* 279.08, found *m/z* 279.34.

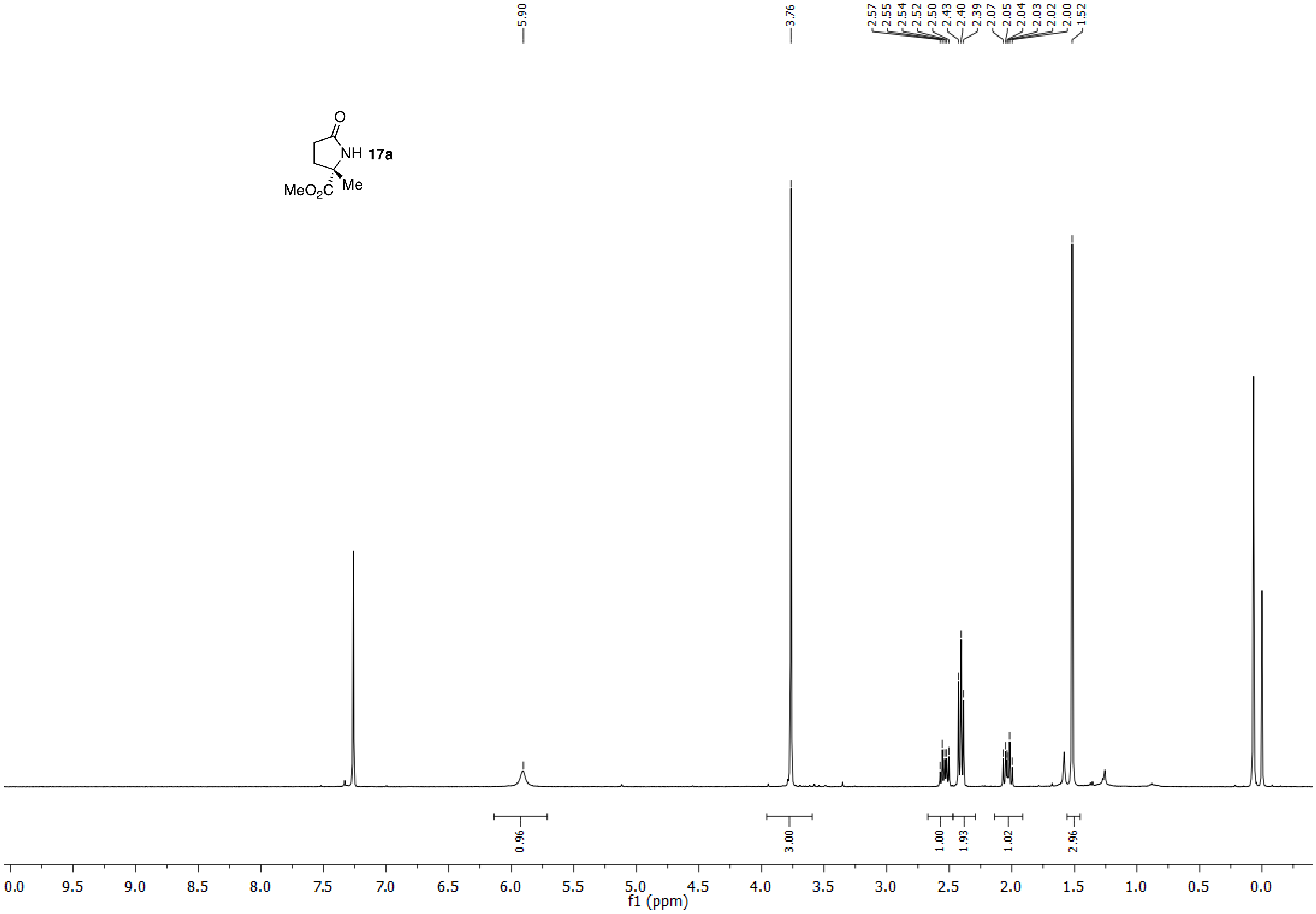
5.90
3.76
2.57
2.55
2.54
2.52
2.50
2.43
2.40
2.39
2.07
2.05
2.04
2.03
2.02
2.00
1.52
O
NH
17a
MeO2C
Me
0.96
3.00
1.00
1.93
1.02
2.96
0.0
9.5
9.0
8.5
8.0
7.5
7.0
6.5
6.0
5.5
5.0
4.5
4.0
3.5
3.0
2.5
2.0
1.5
1.0
0.5
0.0
f1 (ppm)

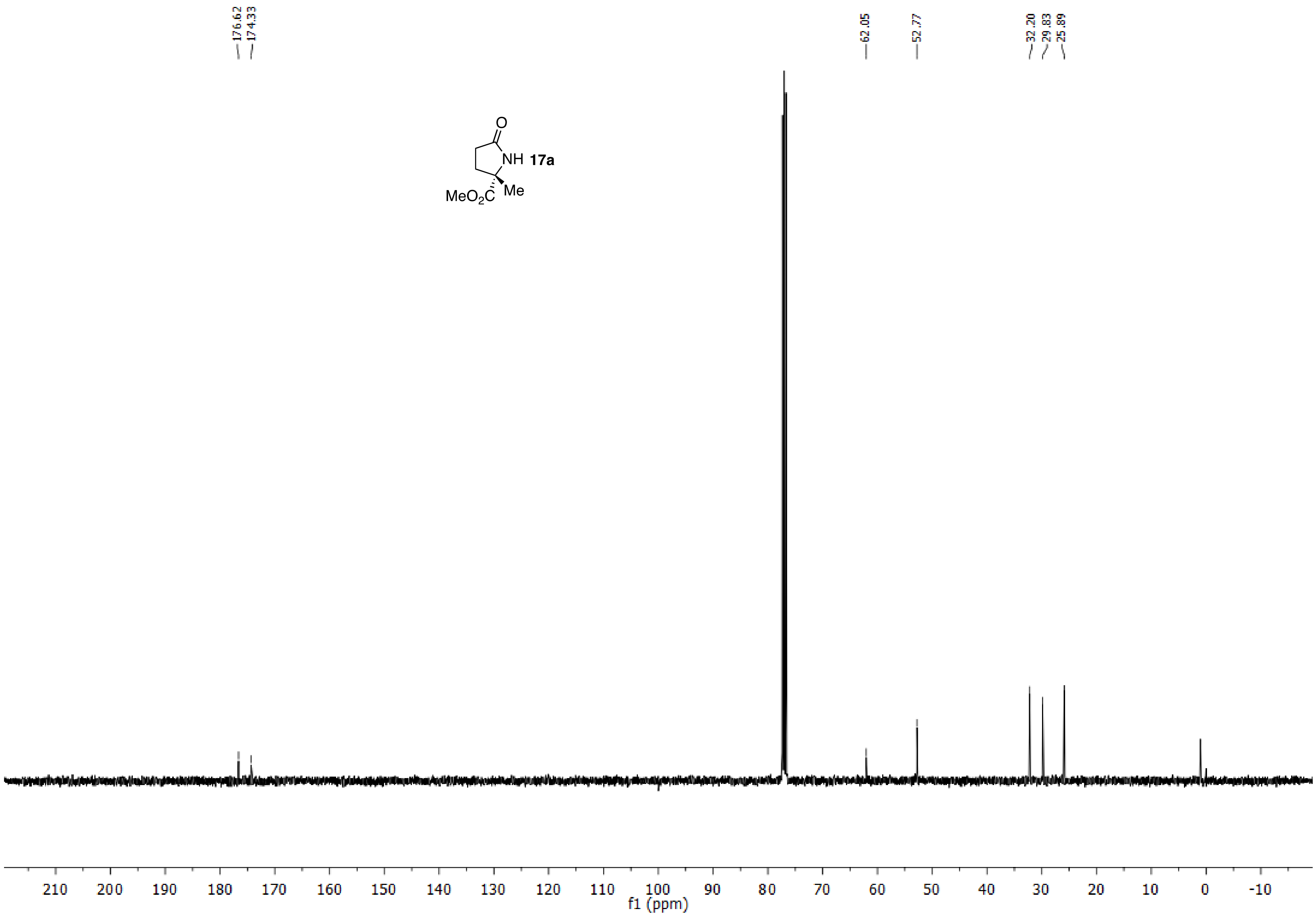
176.62
174.33
62.05
52.77
32.20
29.83
25.89
O
NH 17a
MeO2C
Me
210 200 190 180 170 160 150 140 130 120 110 100 90 80 70 60 50 40 30 20 10 0 -10
f1 (ppm)

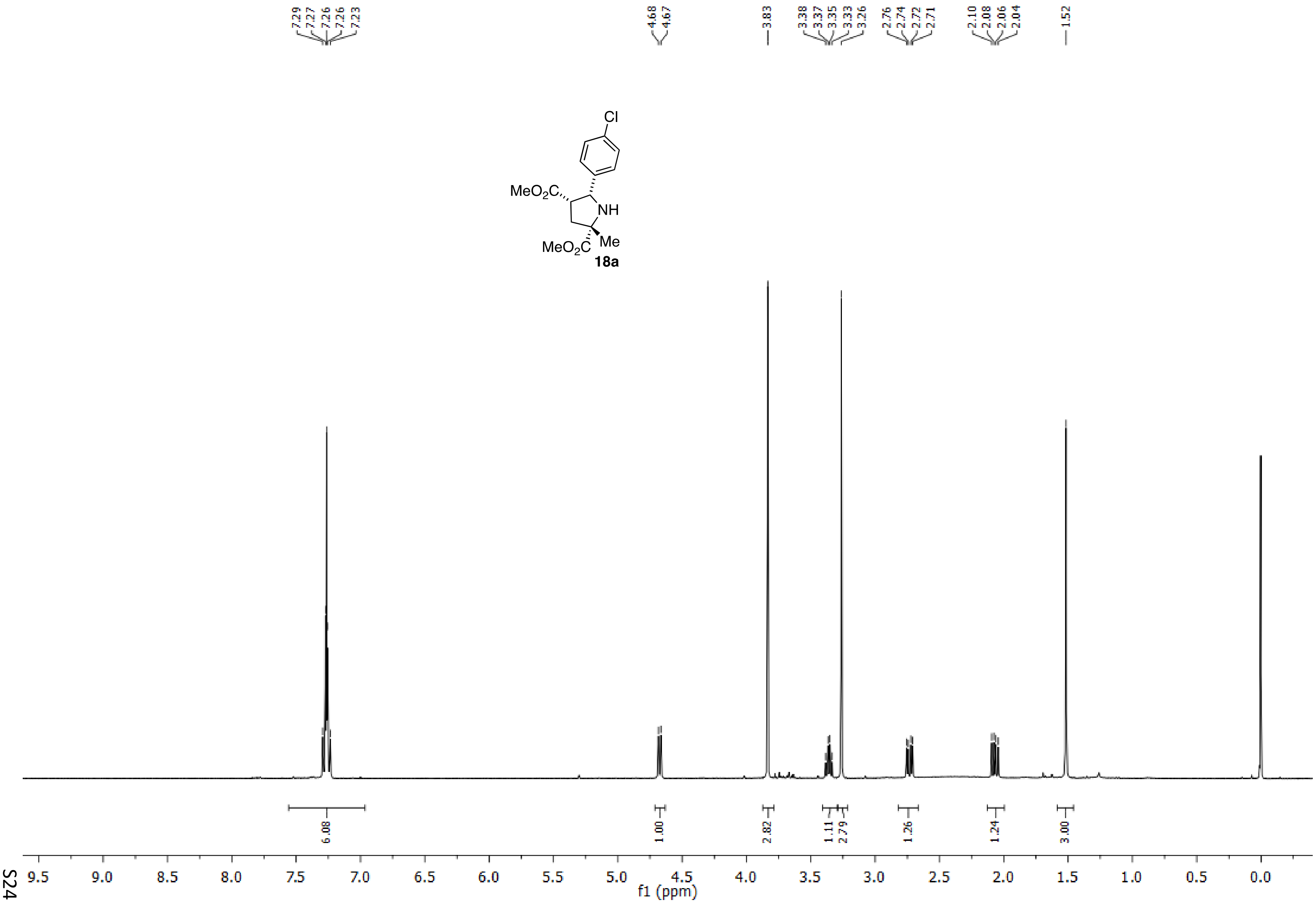
7.29
7.27
7.26
7.26
7.23
4.68
4.67
3.83
3.38
3.37
3.35
3.33
3.26
2.76
2.74
2.72
2.71
2.10
2.08
2.06
2.04
1.52
Cl
MeO2C
NH
MeO2C
Me
18a
6.08
1.00
2.82
1.11
2.79
1.26
1.24
3.00
9.5
9.0
8.5
8.0
7.5
7.0
6.5
6.0
5.5
5.0
4.5
4.0
3.5
3.0
2.5
2.0
1.5
1.0
0.5
0.0
f1 (ppm)

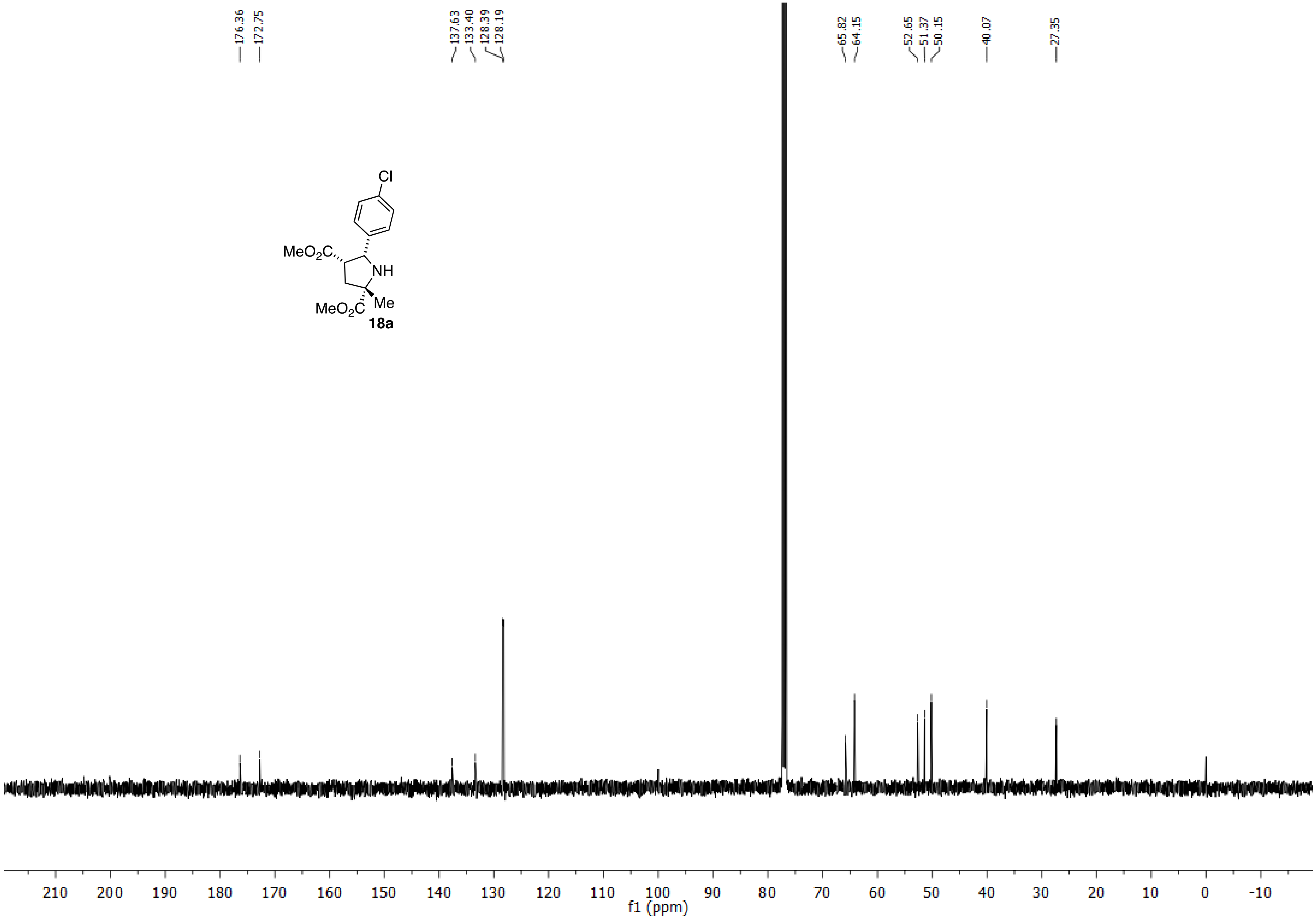

176.36
172.75
137.63
133.40
128.39
128.19
65.82
64.15
52.65
51.37
50.15
40.07
27.35
18a
210
200
190
180
170
160
150
140
130
120
110
100
90
80
70
60
50
40
30
20
10
0
-10
f1 (ppm)

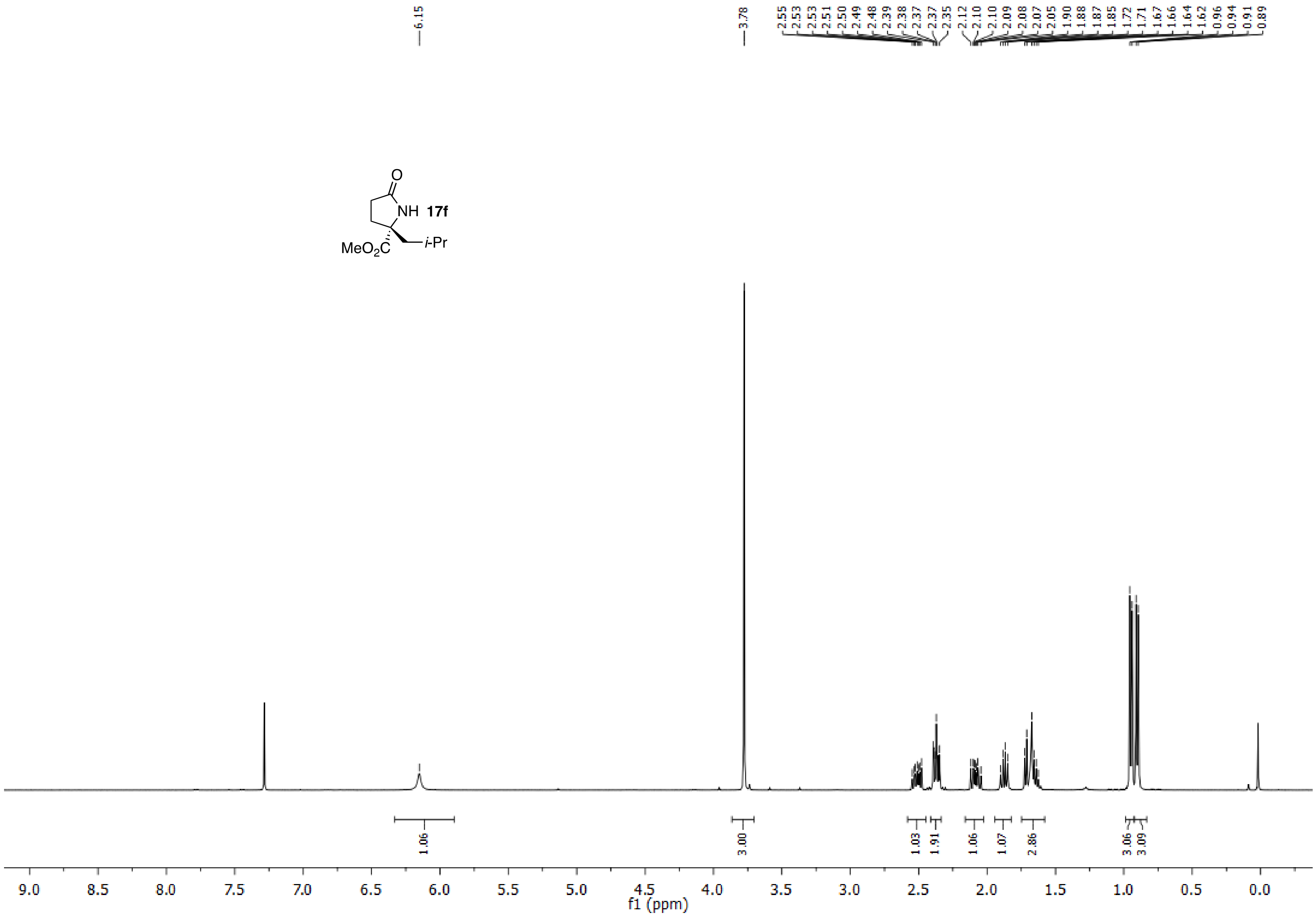

6.15
3.78
2.55 2.53 2.53 2.51 2.50 2.49 2.48 2.39 2.38 2.37 2.37 2.35 2.12 2.10 2.10 2.09 2.08 2.07 2.05 1.90 1.88 1.87 1.85 1.72 1.71 1.67 1.66 1.64 1.62 0.96 0.94 0.91 0.89
NH 17f
MeO2C
i-Pr
1.06
3.00
1.03
1.91
1.06
1.07
2.86
3.06
3.09
9.0 8.5 8.0 7.5 7.0 6.5 6.0 5.5 5.0 4.5 4.0 3.5 3.0 2.5 2.0 1.5 1.0 0.5 0.0
f1 (ppm)

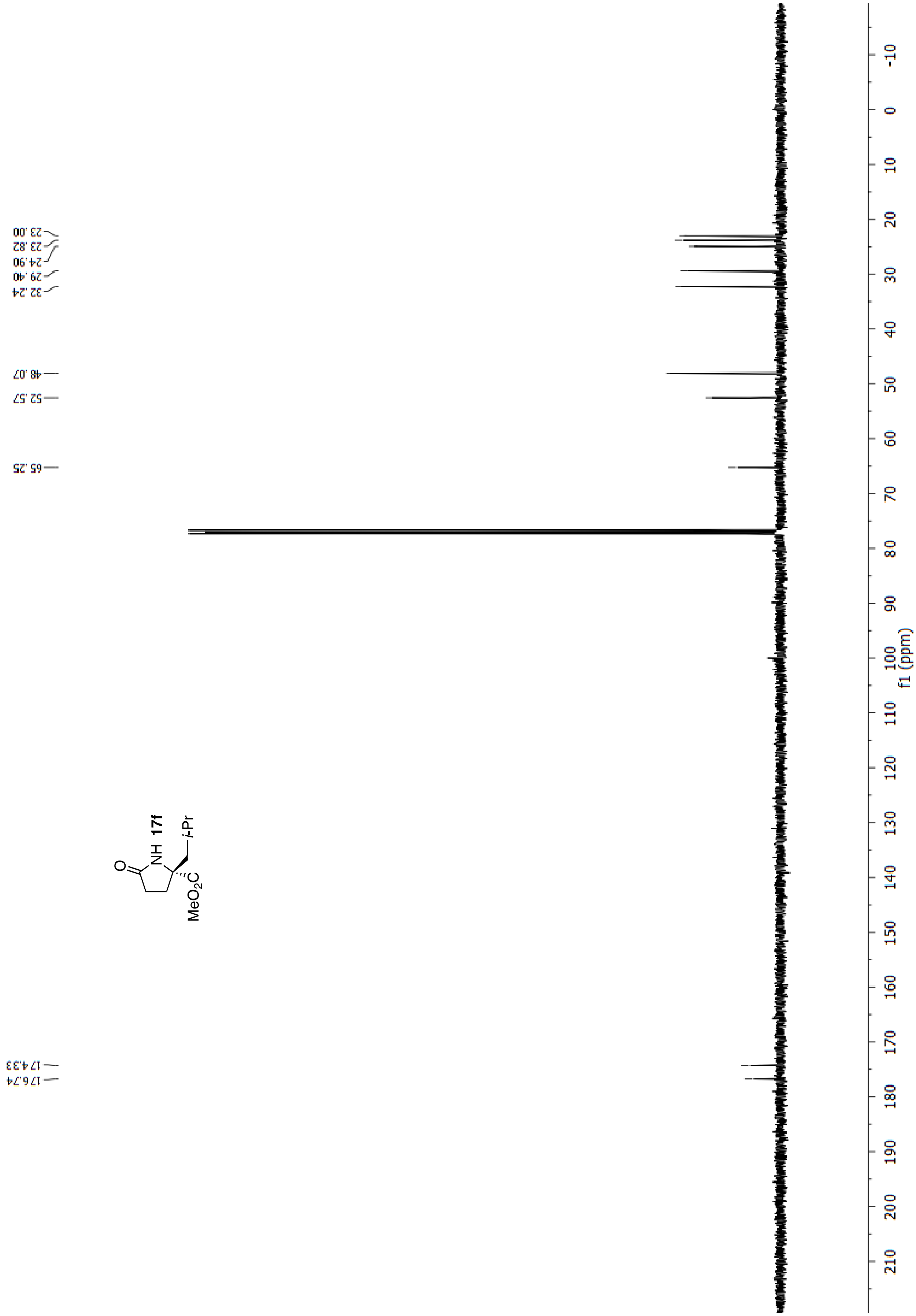
23.00
23.82
24.90
29.40
32.24
48.07
52.57
65.25
174.33
176.74
O
NH 17f
MeO2C
i-Pr
f1 (ppm)

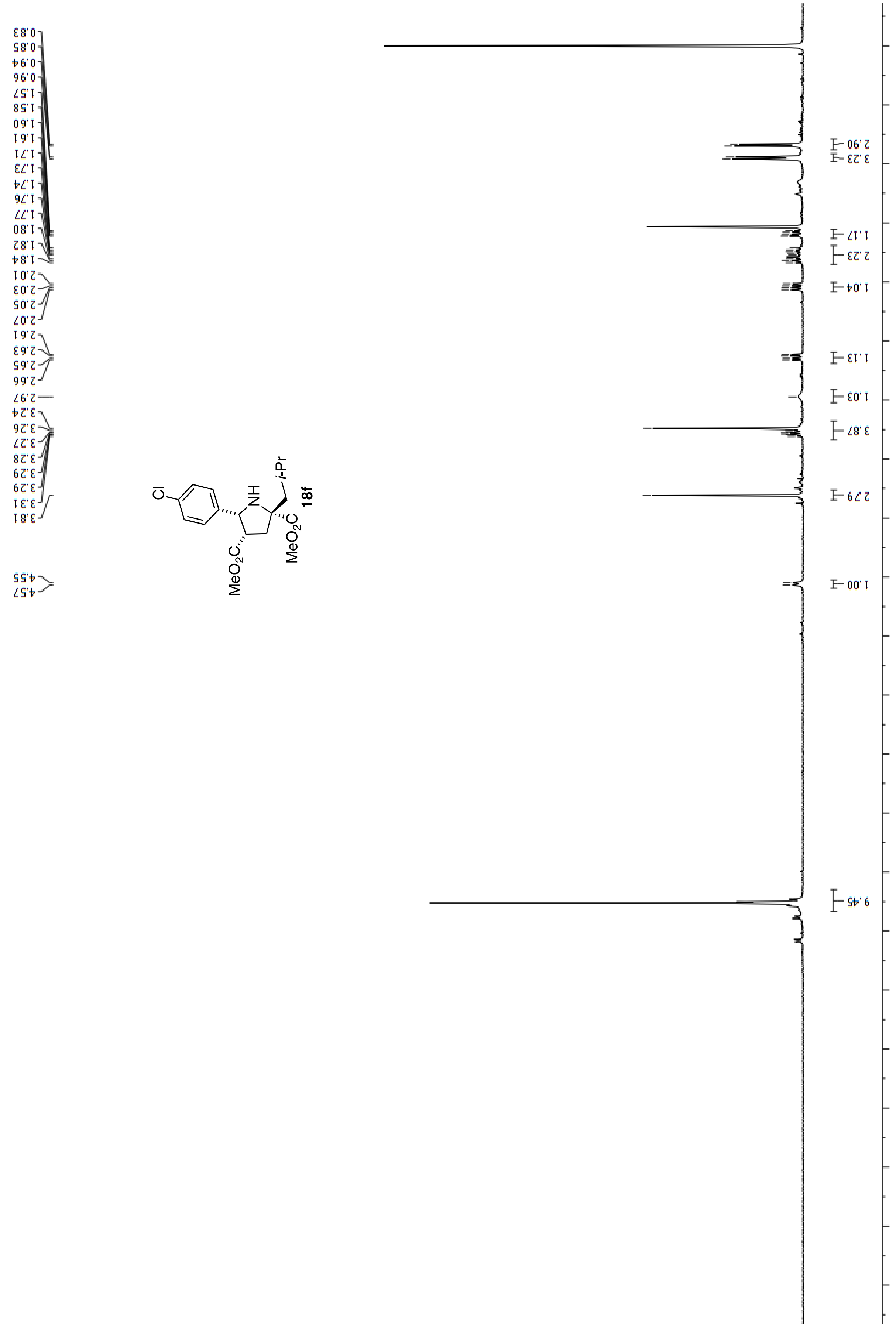

18f
MeO2C
NH
i-Pr
Cl
f1 (ppm)
0.83
0.85
0.94
0.96
1.57
1.58
1.60
1.61
1.71
1.73
1.74
1.76
1.77
1.80
1.82
1.84
2.01
2.03
2.05
2.07
2.61
2.63
2.65
2.66
2.97
3.24
3.26
3.27
3.28
3.29
3.29
3.31
3.81
4.55
4.57
2.90
3.23
1.17
2.23
1.04
1.13
1.03
3.87
2.79
1.00
9.45

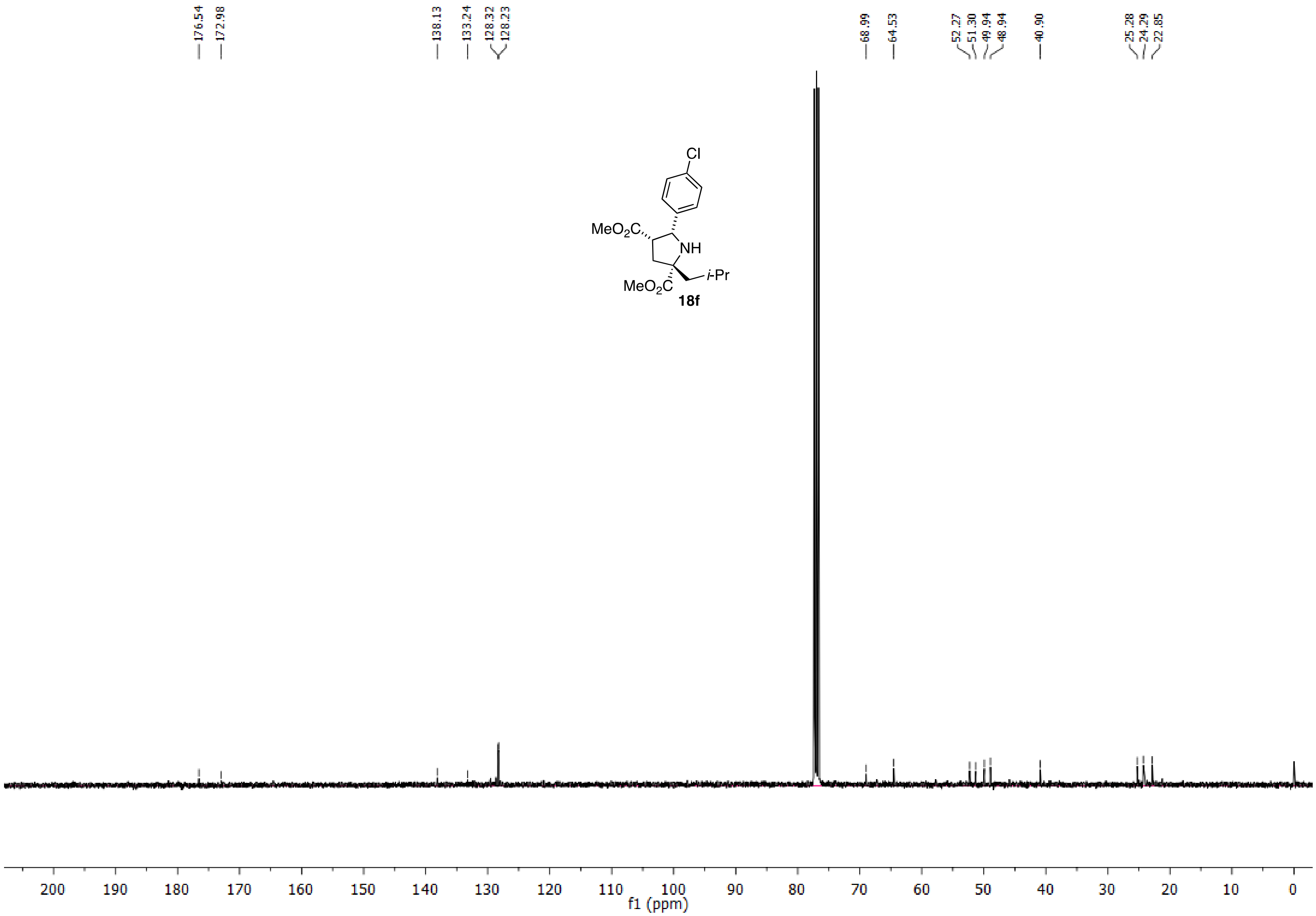
176.54
172.98
138.13
133.24
128.32
128.23
68.99
64.53
52.27
51.30
49.94
48.94
40.90
25.28
24.29
22.85
Cl
MeO2C
NH
MeO2C
i-Pr
18f
200 190 180 170 160 150 140 130 120 110 100 90 80 70 60 50 40 30 20 10 0
f1 (ppm)

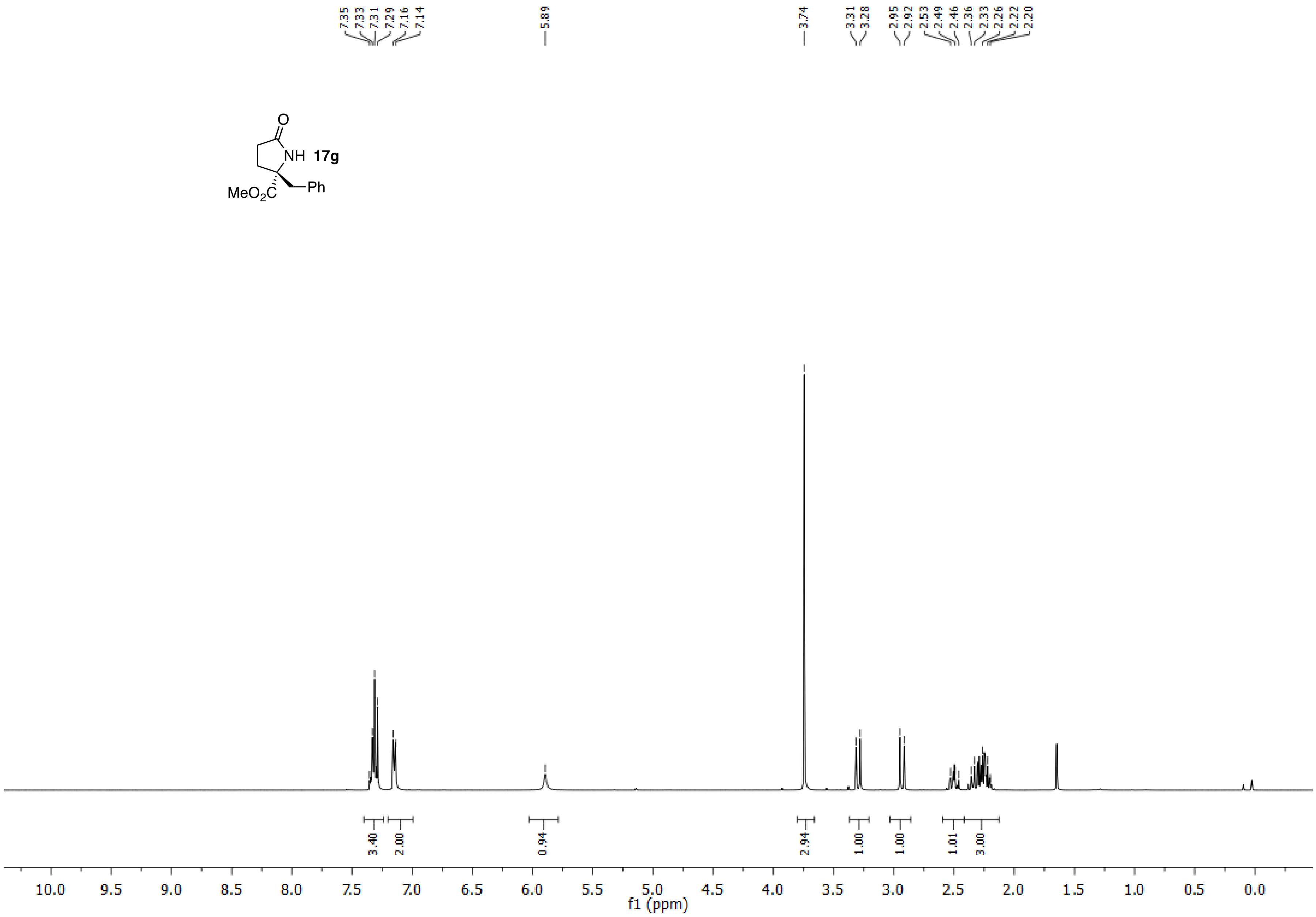
17g
7.35
7.33
7.31
7.29
7.16
7.14
5.89
3.74
3.31
3.28
2.95
2.92
2.53
2.49
2.46
2.36
2.33
2.26
2.22
2.20
3.40
2.00
0.94
2.94
1.00
1.00
1.01
3.00
10.0
9.5
9.0
8.5
8.0
7.5
7.0
6.5
6.0
5.5
5.0
4.5
4.0
3.5
3.0
2.5
2.0
1.5
1.0
0.5
0.0
f1 (ppm)

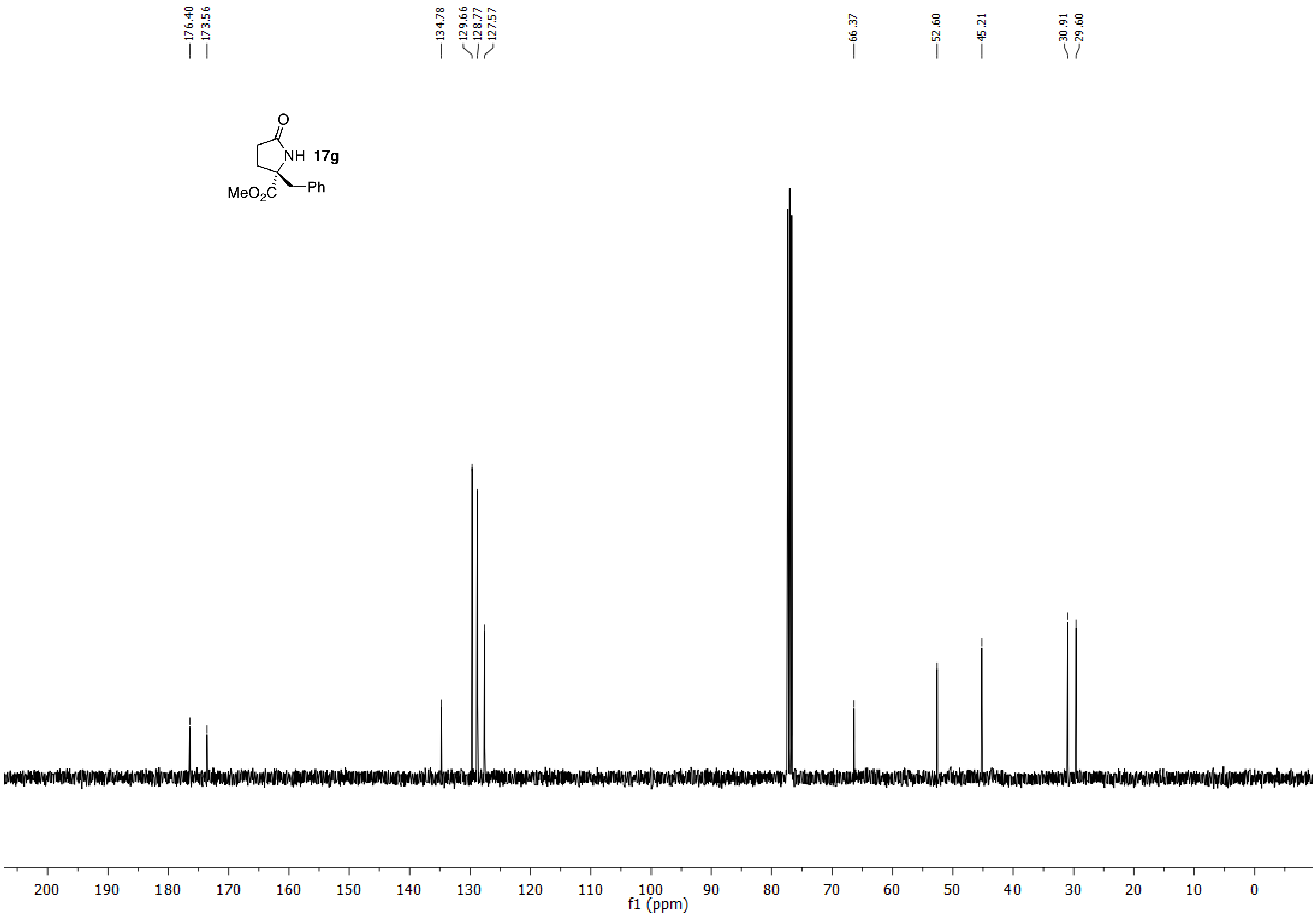
176.40
173.56
134.78
129.66
128.77
127.57
66.37
52.60
45.21
30.91
29.60
NH 17g
MeO2C
Ph
200 190 180 170 160 150 140 130 120 110 100 90 80 70 60 50 40 30 20 10 0
f1 (ppm)

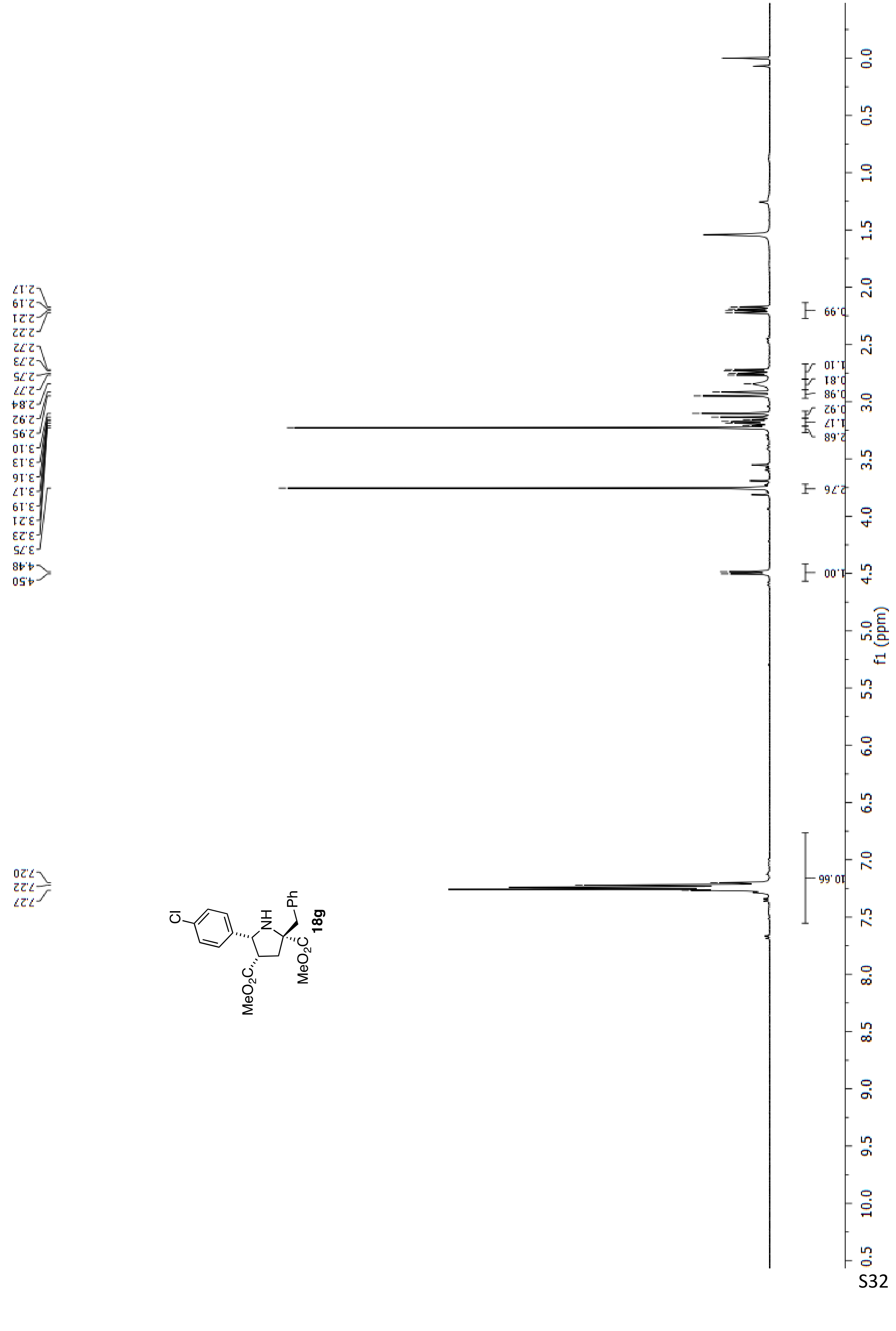

7.27
7.22
7.20
4.50
4.48
3.75
3.23
3.21
3.19
3.17
3.16
3.13
3.10
2.95
2.92
2.84
2.77
2.75
2.73
2.72
2.22
2.21
2.19
2.17
Cl
NH
Ph
MeO2C
MeO2C
18g
10.66
1.00
2.76
2.68
1.17
0.92
0.98
0.81
1.10
0.99
f1 (ppm)

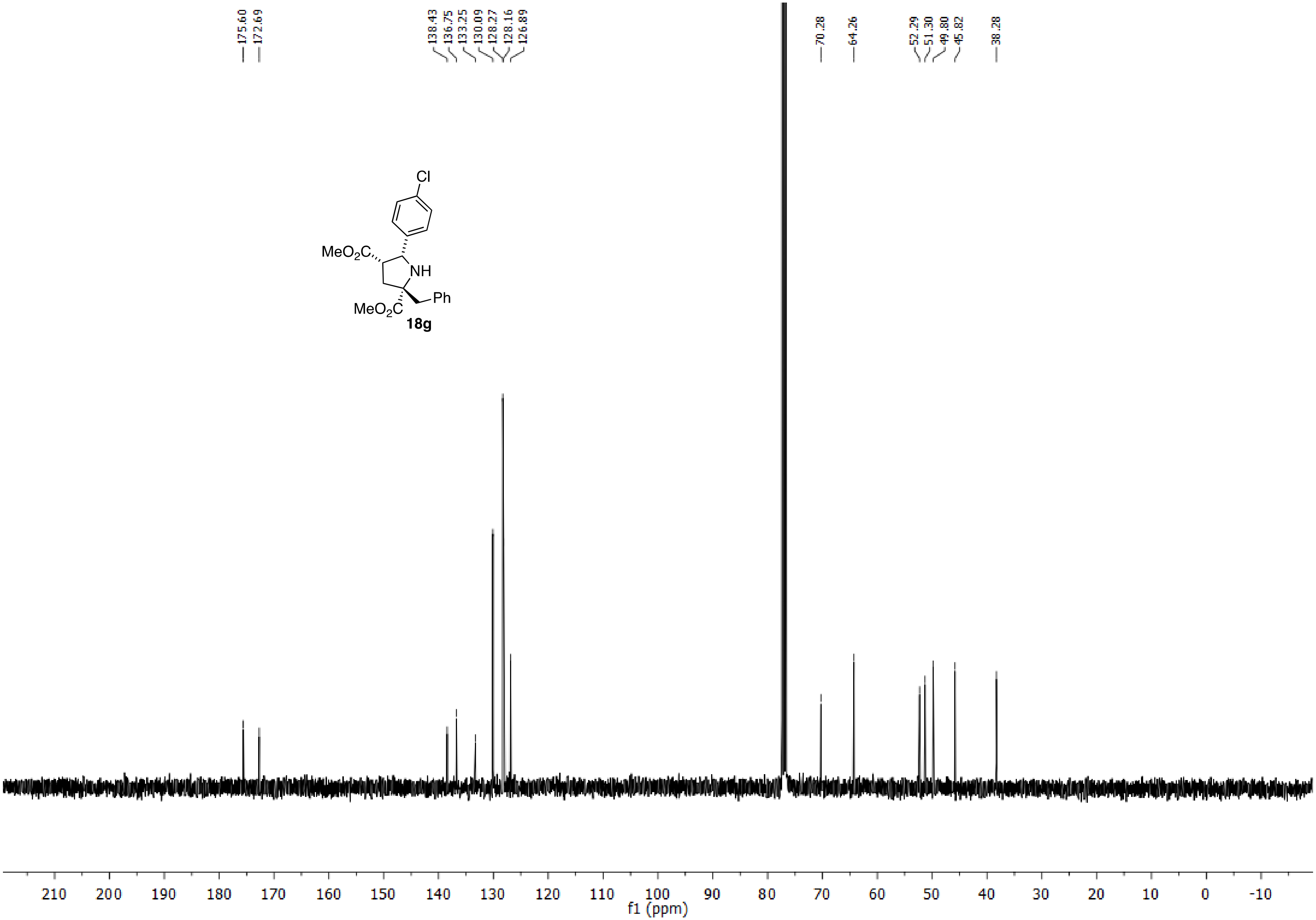

175.60
172.69
138.43
136.75
133.25
130.09
128.27
128.16
126.89
70.28
64.26
52.29
51.30
49.80
45.82
38.28
Cl
MeO2C
NH
Ph
MeO2C
18g
210 200 190 180 170 160 150 140 130 120 110 100 90 80 70 60 50 40 30 20 10 0 -10
f1 (ppm)

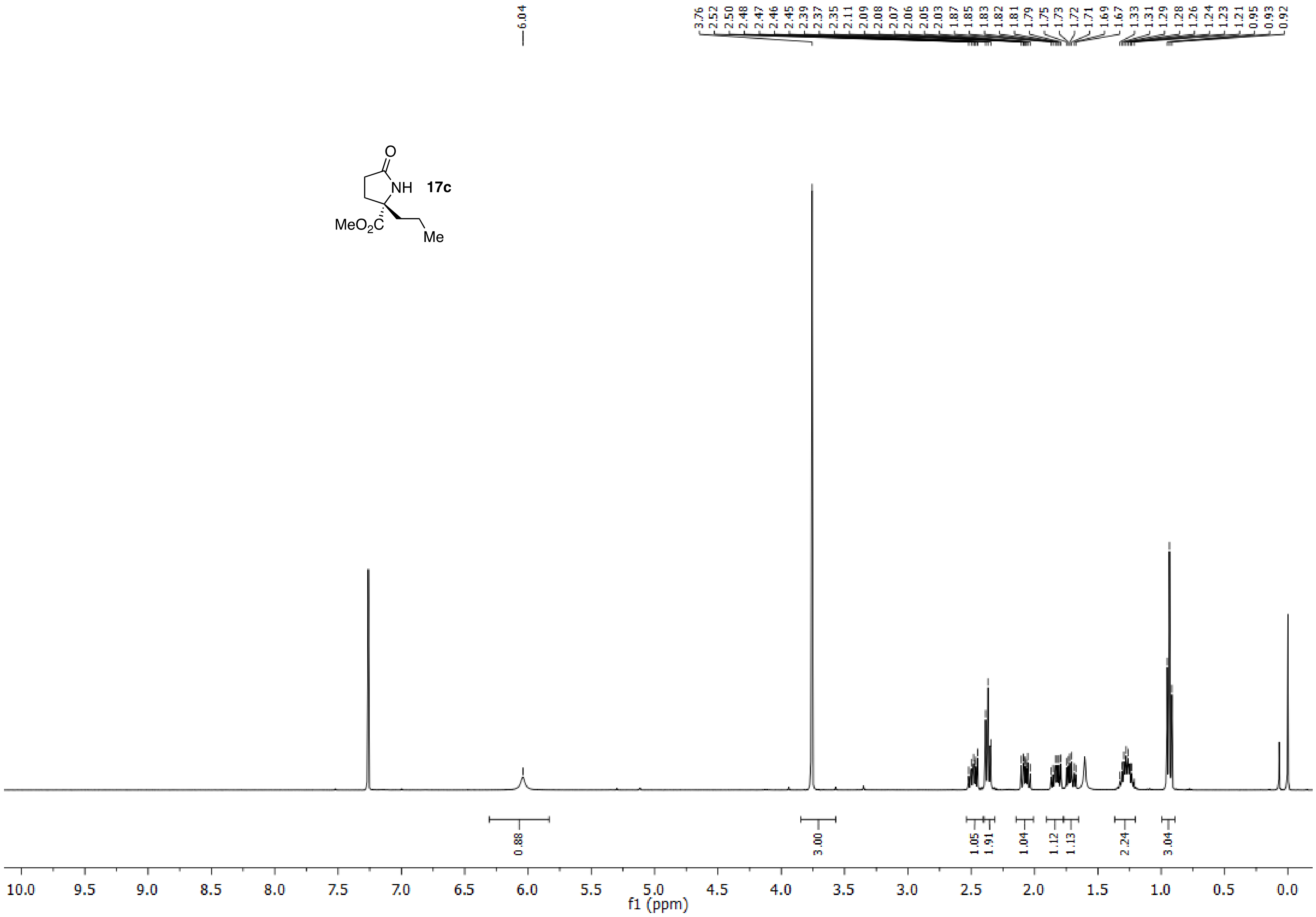
6.04
3.76 2.52 2.50 2.48 2.47 2.46 2.45 2.39 2.37 2.35 2.11 2.09 2.08 2.07 2.06 2.05 2.03 1.87 1.85 1.83 1.82 1.81 1.79 1.75 1.73 1.72 1.71 1.69 1.67 1.33 1.31 1.29 1.28 1.26 1.24 1.23 1.21 0.95 0.93 0.92
O
NH
17c
MeO2C
Me
0.88
3.00
1.05
1.91
1.04
1.12
1.13
2.24
3.04
10.0 9.5 9.0 8.5 8.0 7.5 7.0 6.5 6.0 5.5 5.0 4.5 4.0 3.5 3.0 2.5 2.0 1.5 1.0 0.5 0.0
f1 (ppm)

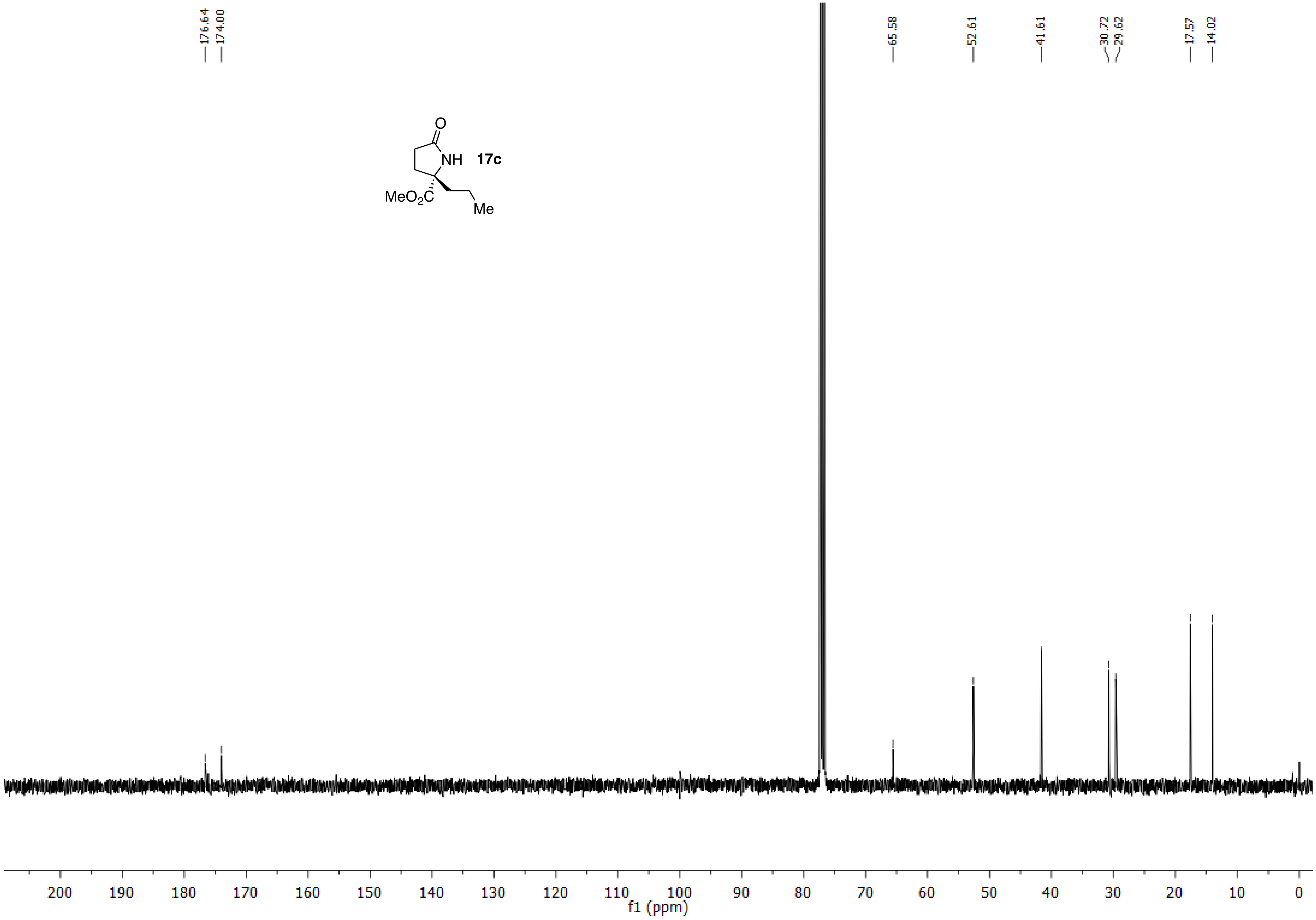
176.64
174.00
65.58
52.61
41.61
30.72
29.62
17.57
14.02
O
NH
17c
MeO$_2$C
Me
200 190 180 170 160 150 140 130 120 110 100 90 80 70 60 50 40 30 20 10 0
f1 (ppm)

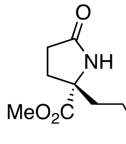

17c + Eu(hfc)$_3$

racemic

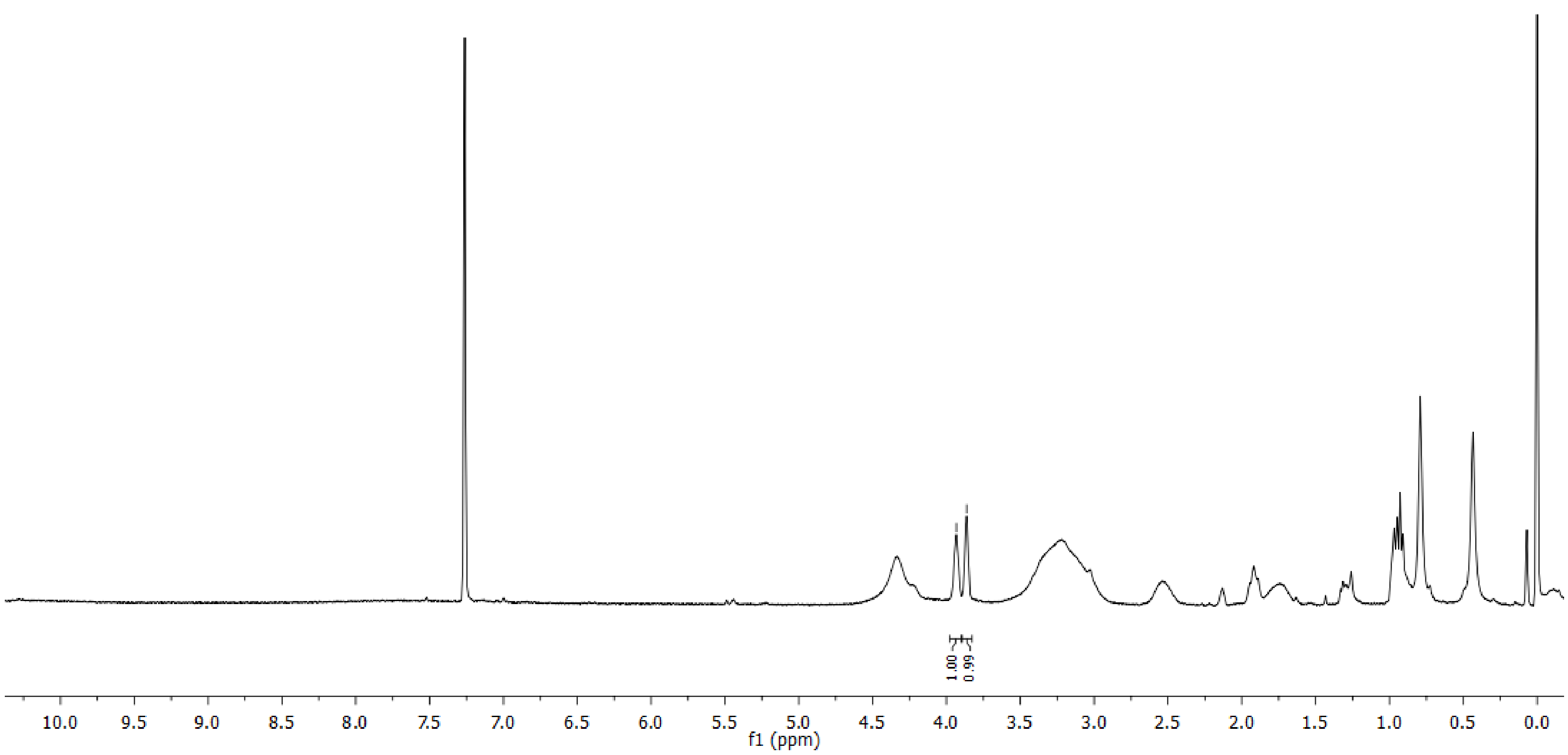

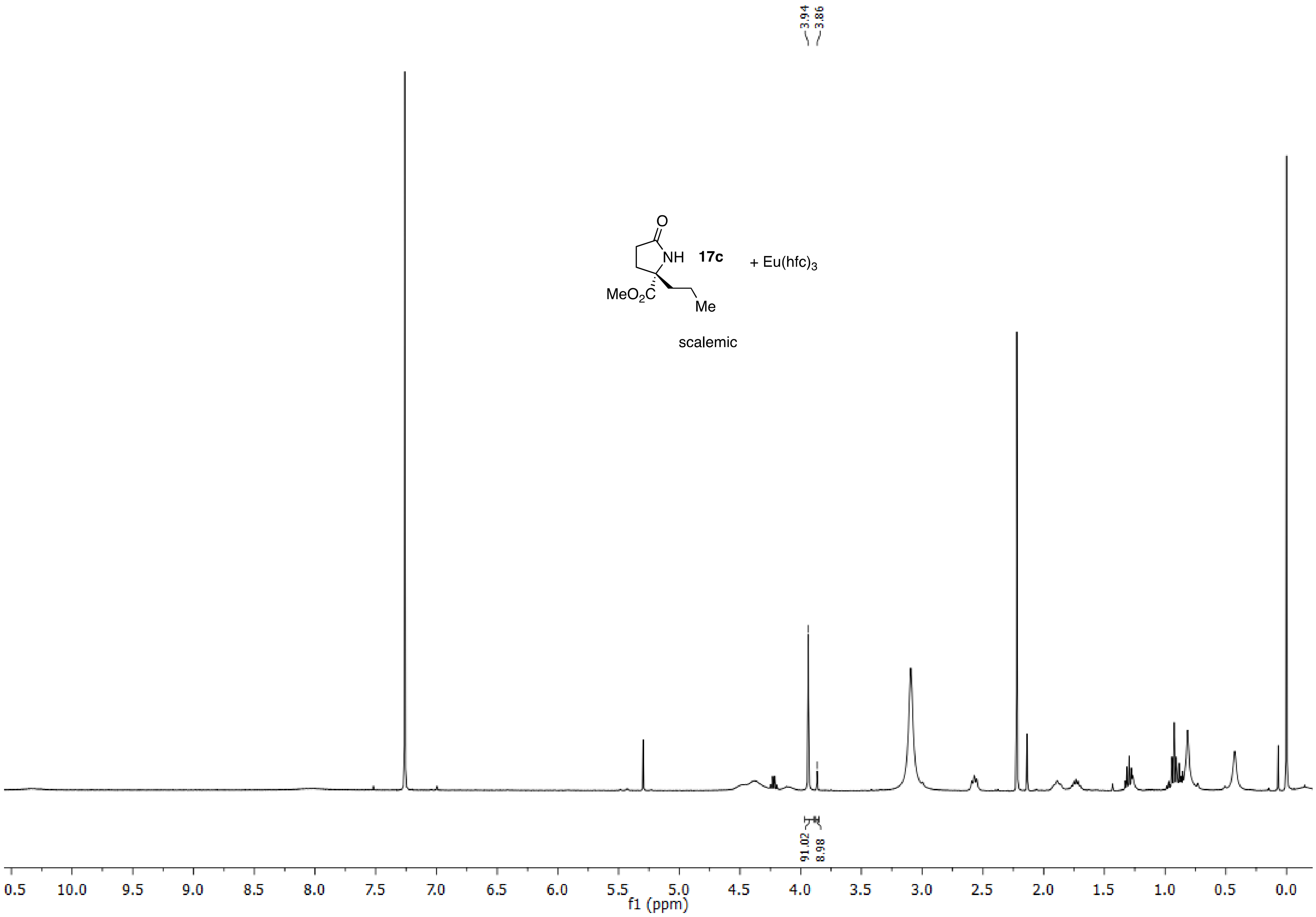
3.94
3.86
O
NH
17c
+ Eu(hfc)$_3$
MeO$_2$C
Me
scalemic
91.02
8.98
10.5 10.0 9.5 9.0 8.5 8.0 7.5 7.0 6.5 6.0 5.5 5.0 4.5 4.0 3.5 3.0 2.5 2.0 1.5 1.0 0.5 0.0
f1 (ppm)

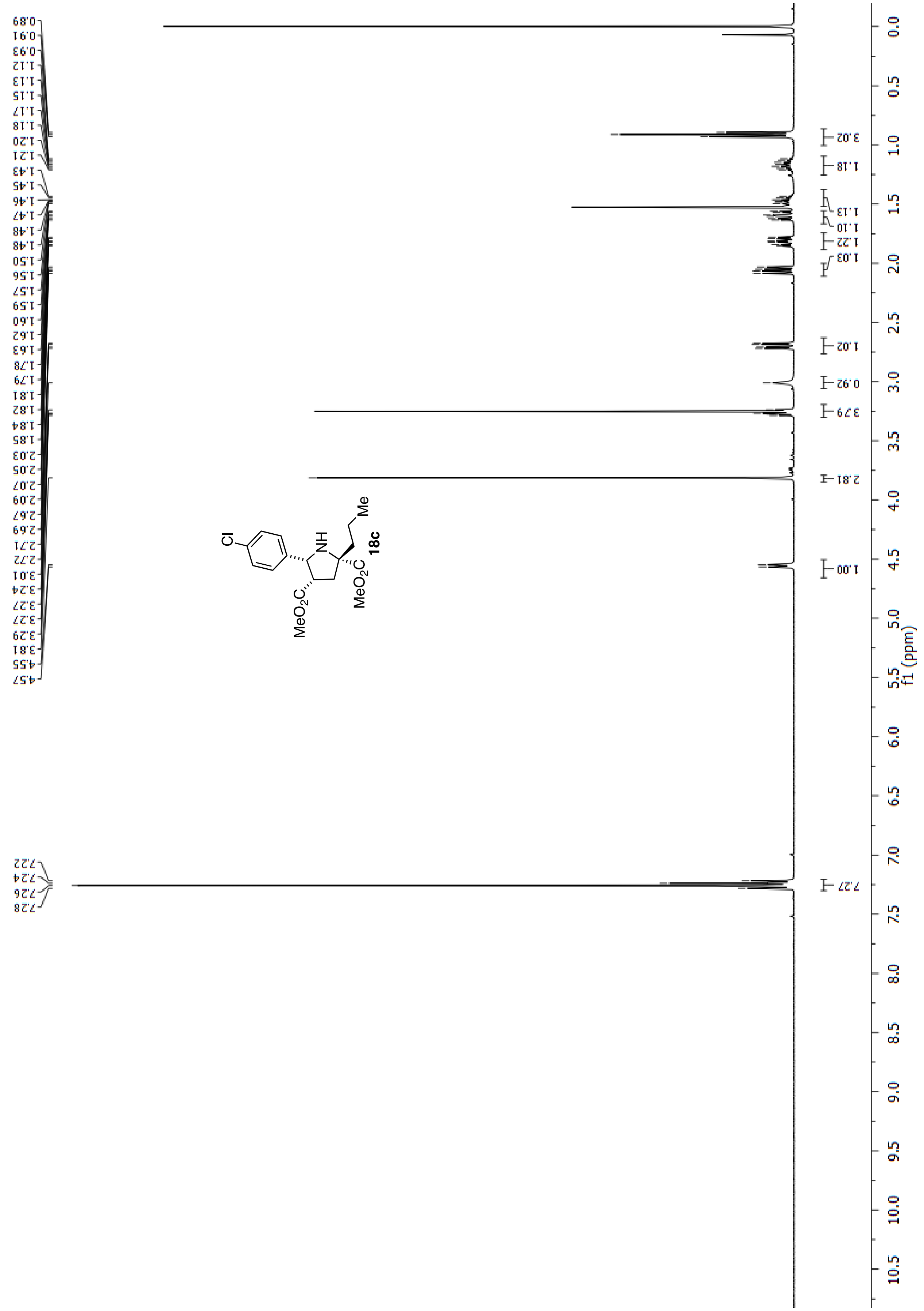
0.89
0.91
0.93
1.12
1.13
1.15
1.17
1.18
1.20
1.21
1.43
1.45
1.46
1.47
1.48
1.48
1.50
1.56
1.57
1.59
1.60
1.62
1.63
1.78
1.79
1.81
1.82
1.84
1.85
2.03
2.05
2.07
2.09
2.67
2.69
2.71
2.72
3.01
3.24
3.27
3.27
3.29
3.81
4.55
4.57
7.22
7.24
7.26
7.28
Cl
NH
Me
MeO2C
MeO2C
18c
3.02
1.18
1.13
1.10
1.22
1.03
1.02
0.92
3.79
2.81
1.00
7.27
f1 (ppm)

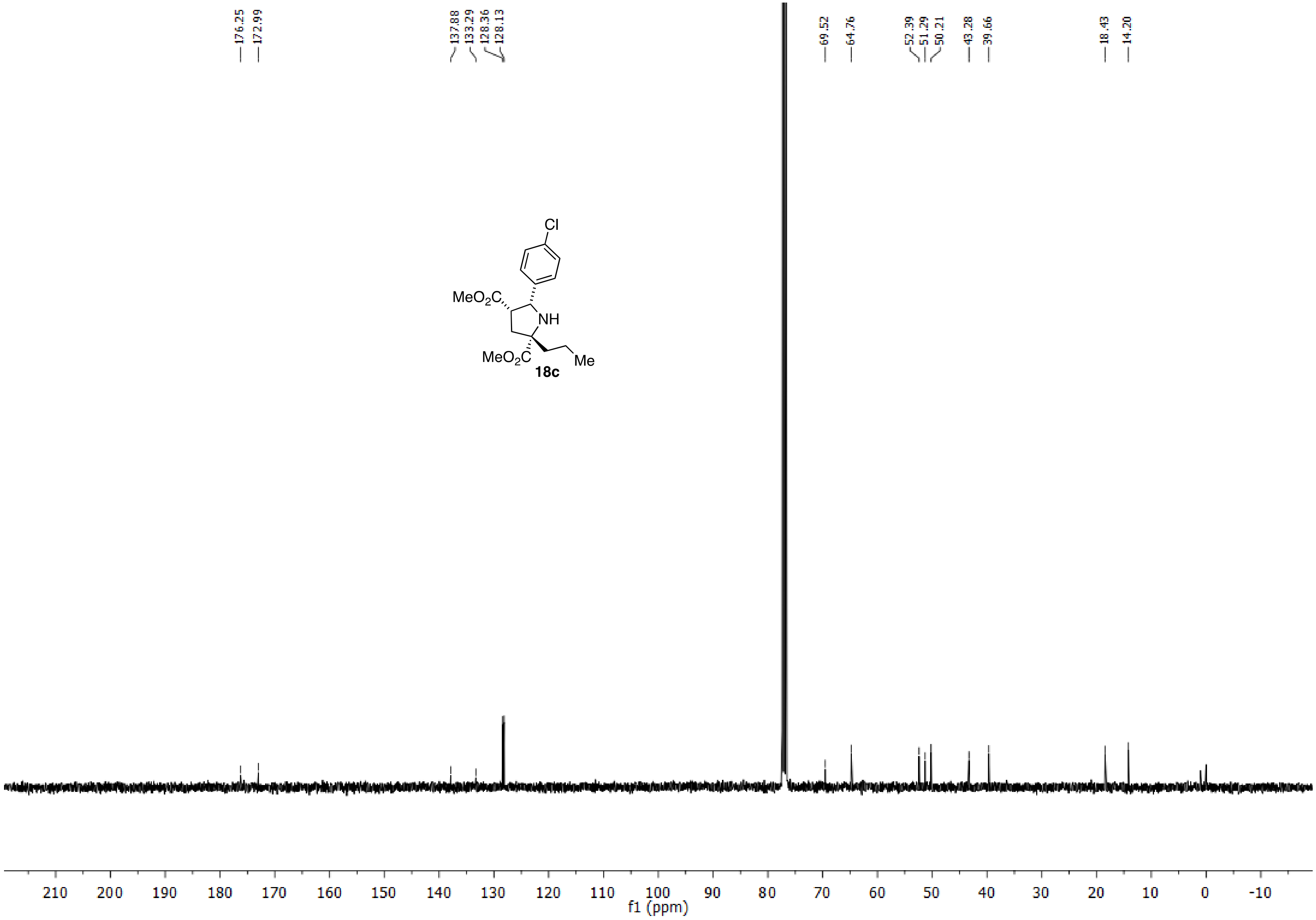
176.25
172.99
137.88
133.29
128.36
128.13
69.52
64.76
52.39
51.29
50.21
43.28
39.66
18.43
14.20
Cl
MeO2C
NH
MeO2C
Me
18c
210 200 190 180 170 160 150 140 130 120 110 100 90 80 70 60 50 40 30 20 10 0 -10
f1 (ppm)

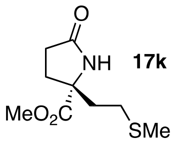

17k

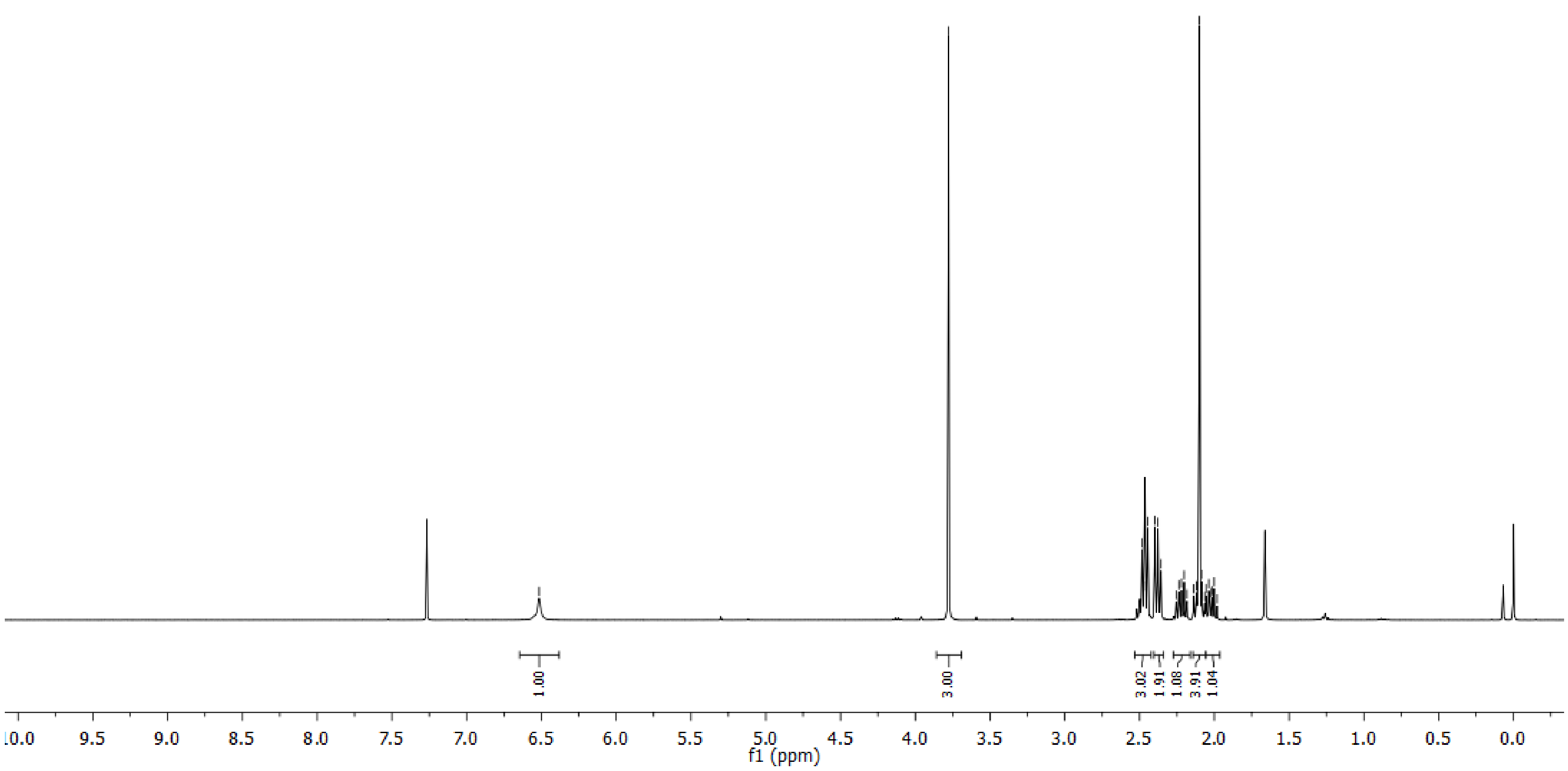
6.51
3.78
2.48
2.48
2.47
2.45
2.40
2.38
2.36
2.26
2.24
2.22
2.20
2.18
2.14
2.12
2.11
2.10
2.09
2.06
2.06
2.04
2.02
2.02
2.00
1.98
1.00
3.00
3.02
1.91
1.08
3.91
1.04
10.0
9.5
9.0
8.5
8.0
7.5
7.0
6.5
6.0
5.5
5.0
4.5
4.0
3.5
3.0
2.5
2.0
1.5
1.0
0.5
0.0
f1 (ppm)

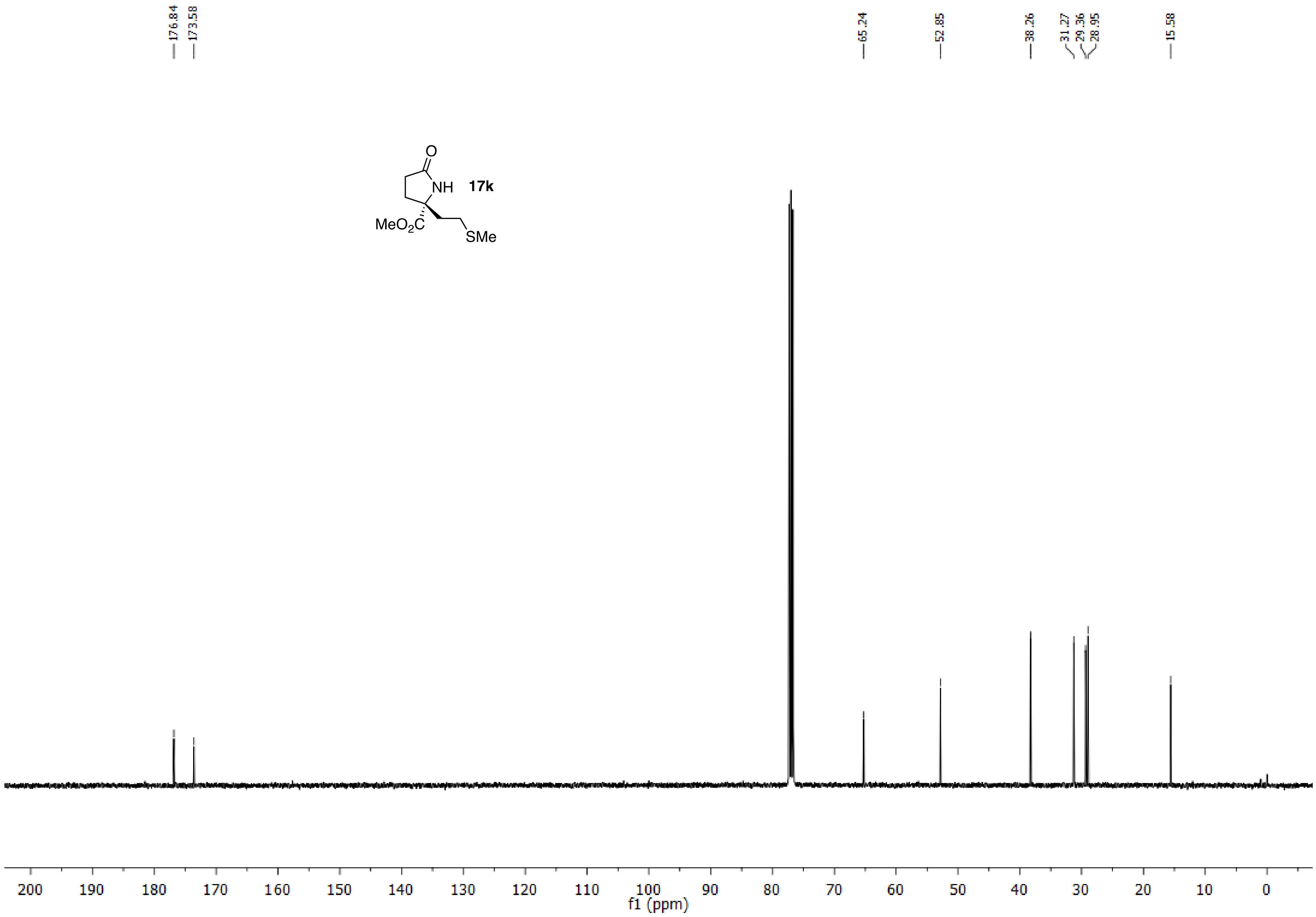
176.84
173.58
65.24
52.85
38.26
31.27
29.36
28.95
15.58
O
NH
17k
MeO2C
SMe
200 190 180 170 160 150 140 130 120 110 100 90 80 70 60 50 40 30 20 10 0
f1 (ppm)

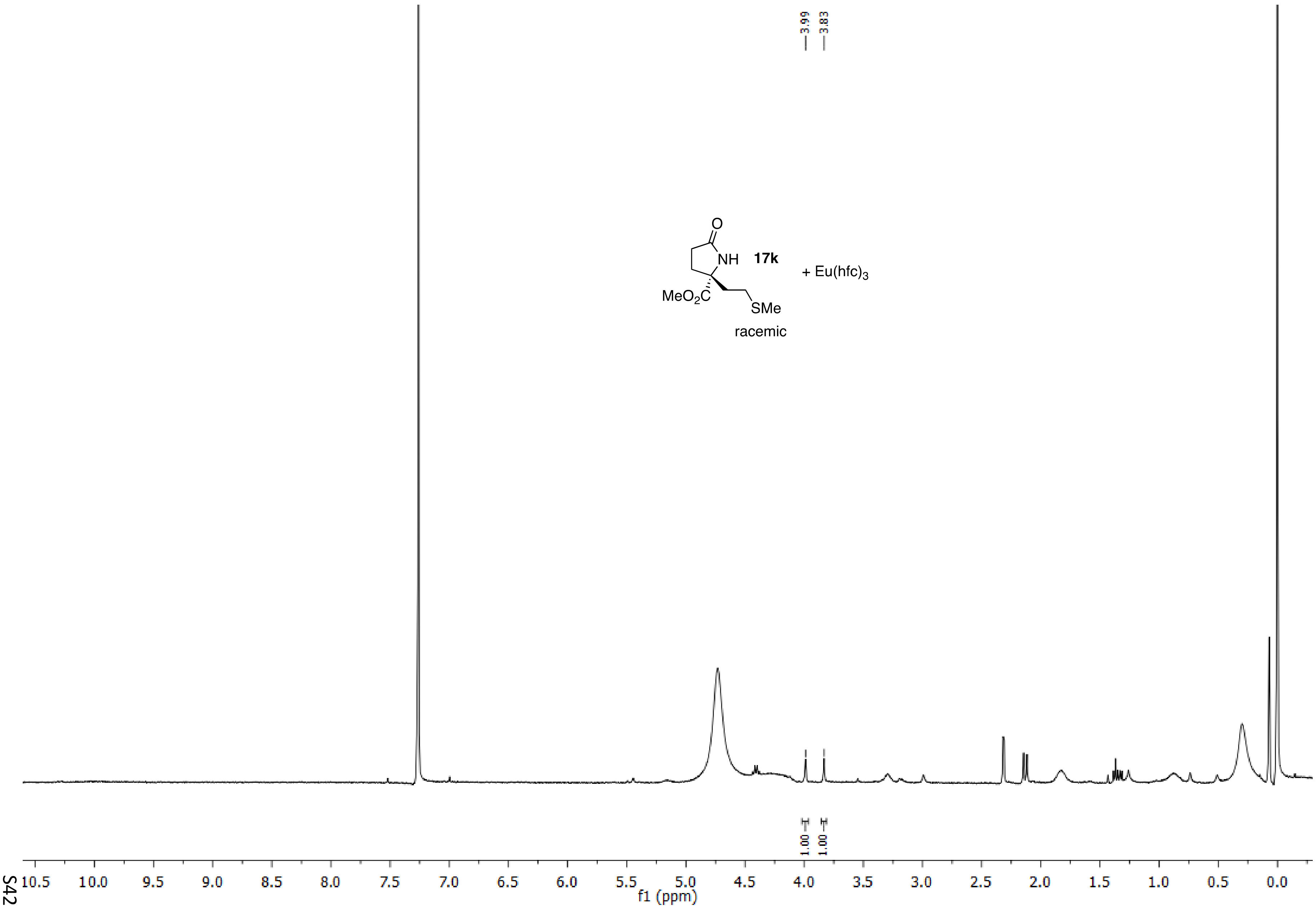
3.99
3.83
NH
O
MeO2C
SMe
17k
+ Eu(hfc)3
racemic
1.00
1.00
10.5 10.0 9.5 9.0 8.5 8.0 7.5 7.0 6.5 6.0 5.5 5.0 4.5 4.0 3.5 3.0 2.5 2.0 1.5 1.0 0.5 0.0
f1 (ppm)

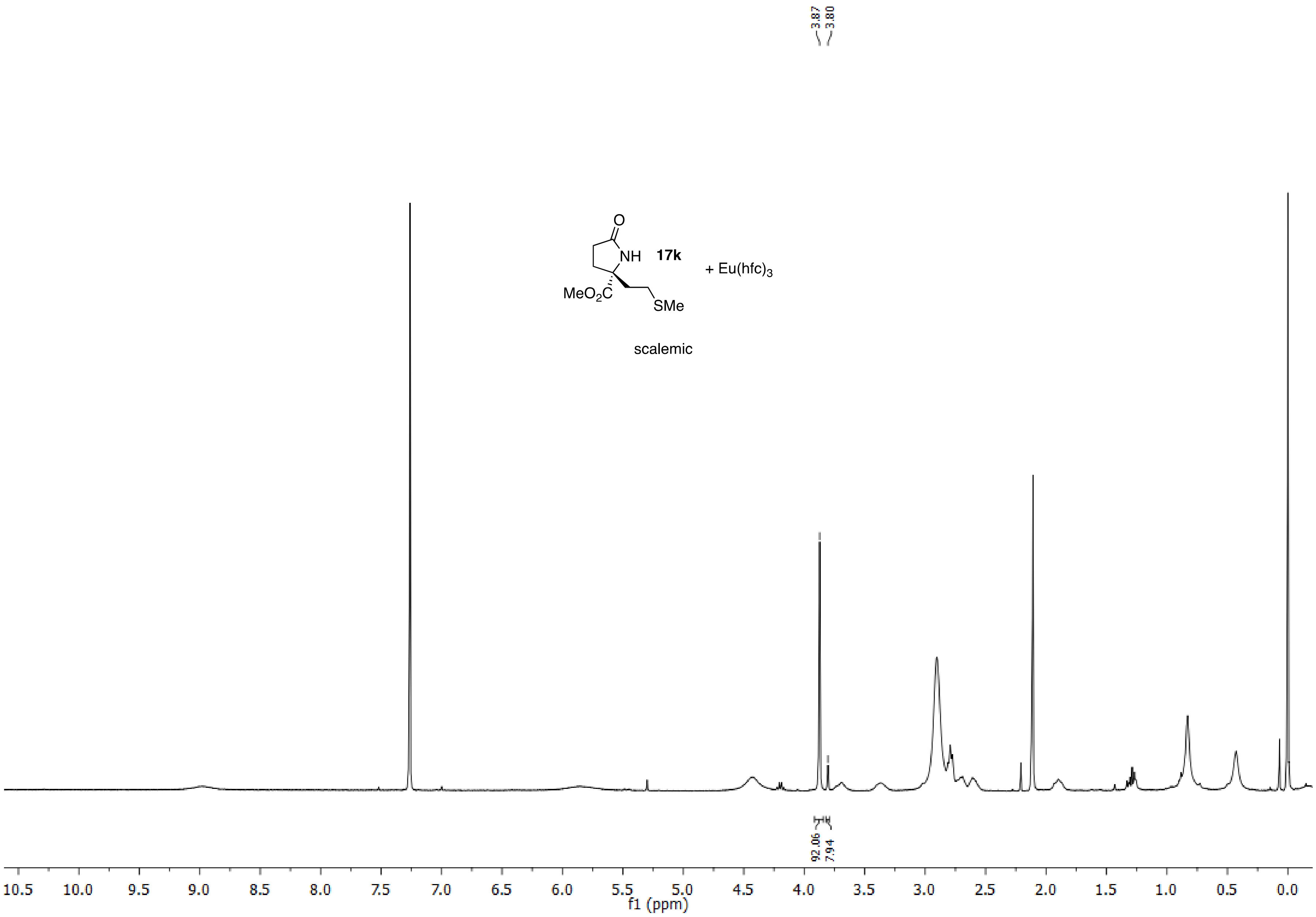
3.87
3.80
17k
+ Eu(hfc)$_3$
scalemic
92.06
7.94
f1 (ppm)

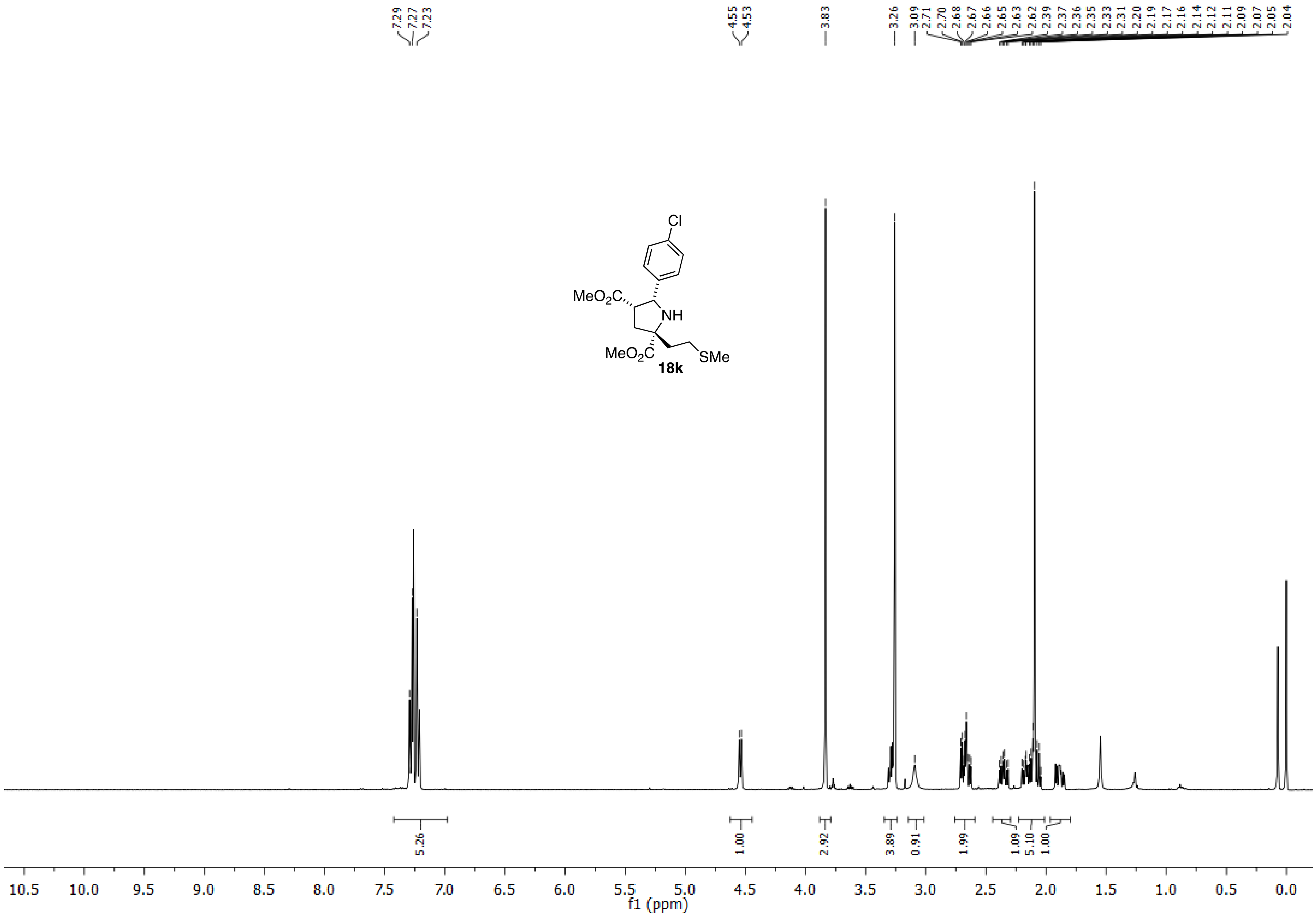
7.29
7.27
7.23
4.55
4.53
3.83
3.26
3.09
2.71
2.70
2.68
2.67
2.66
2.65
2.63
2.62
2.39
2.37
2.36
2.35
2.33
2.31
2.20
2.19
2.17
2.16
2.14
2.12
2.11
2.09
2.07
2.05
2.04
Cl
MeO_2C
NH
MeO_2C
SMe
18k
5.26
1.00
2.92
3.89
0.91
1.99
1.09
5.10
1.00
10.5
10.0
9.5
9.0
8.5
8.0
7.5
7.0
6.5
6.0
5.5
5.0
4.5
4.0
3.5
3.0
2.5
2.0
1.5
1.0
0.5
0.0
f1 (ppm)

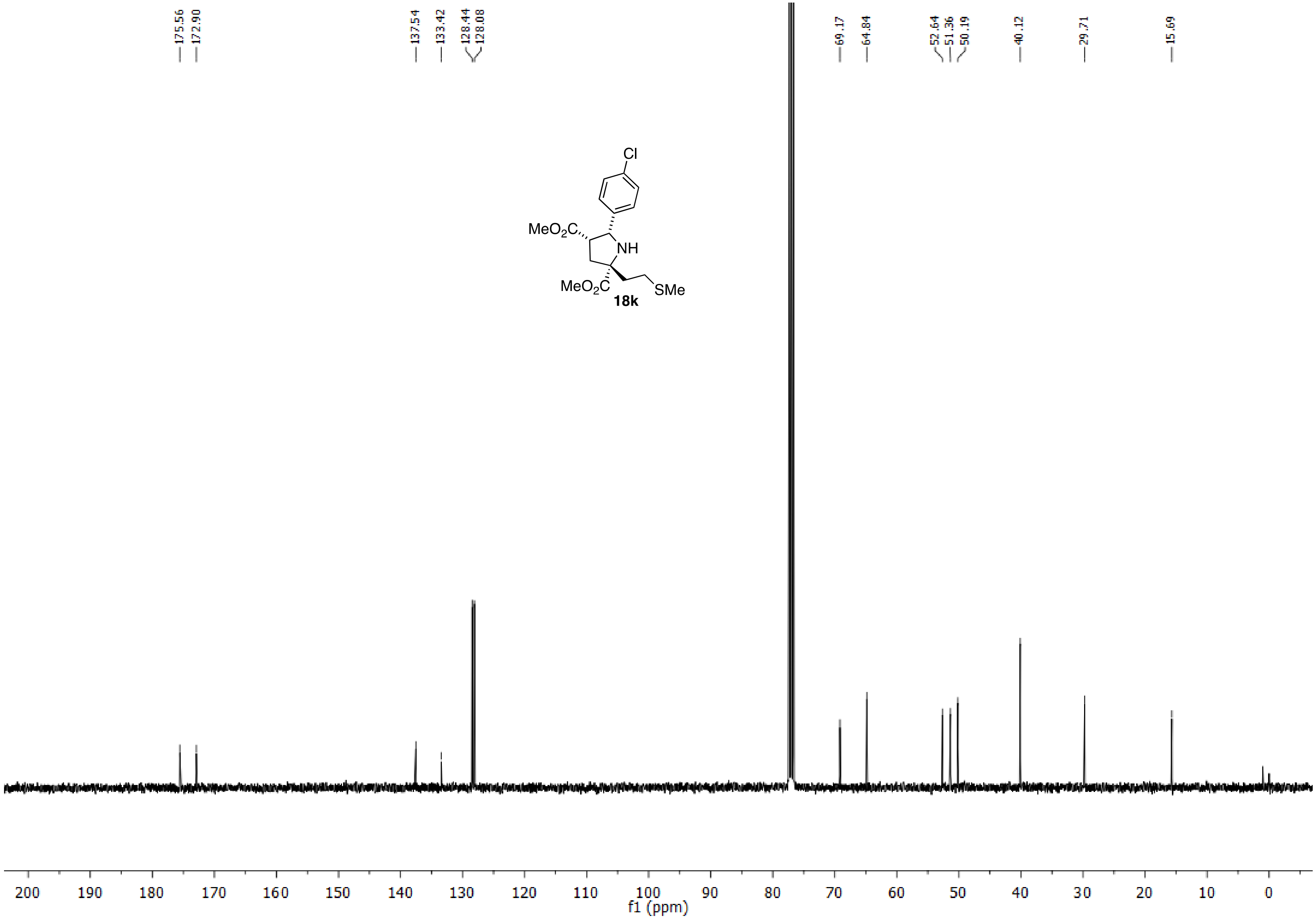
175.56
172.90
137.54
133.42
128.44
128.08
69.17
64.84
52.64
51.36
50.19
40.12
29.71
15.69
Cl
MeO2C
NH
MeO2C
SMe
18k
200 190 180 170 160 150 140 130 120 110 100 90 80 70 60 50 40 30 20 10 0
f1 (ppm)

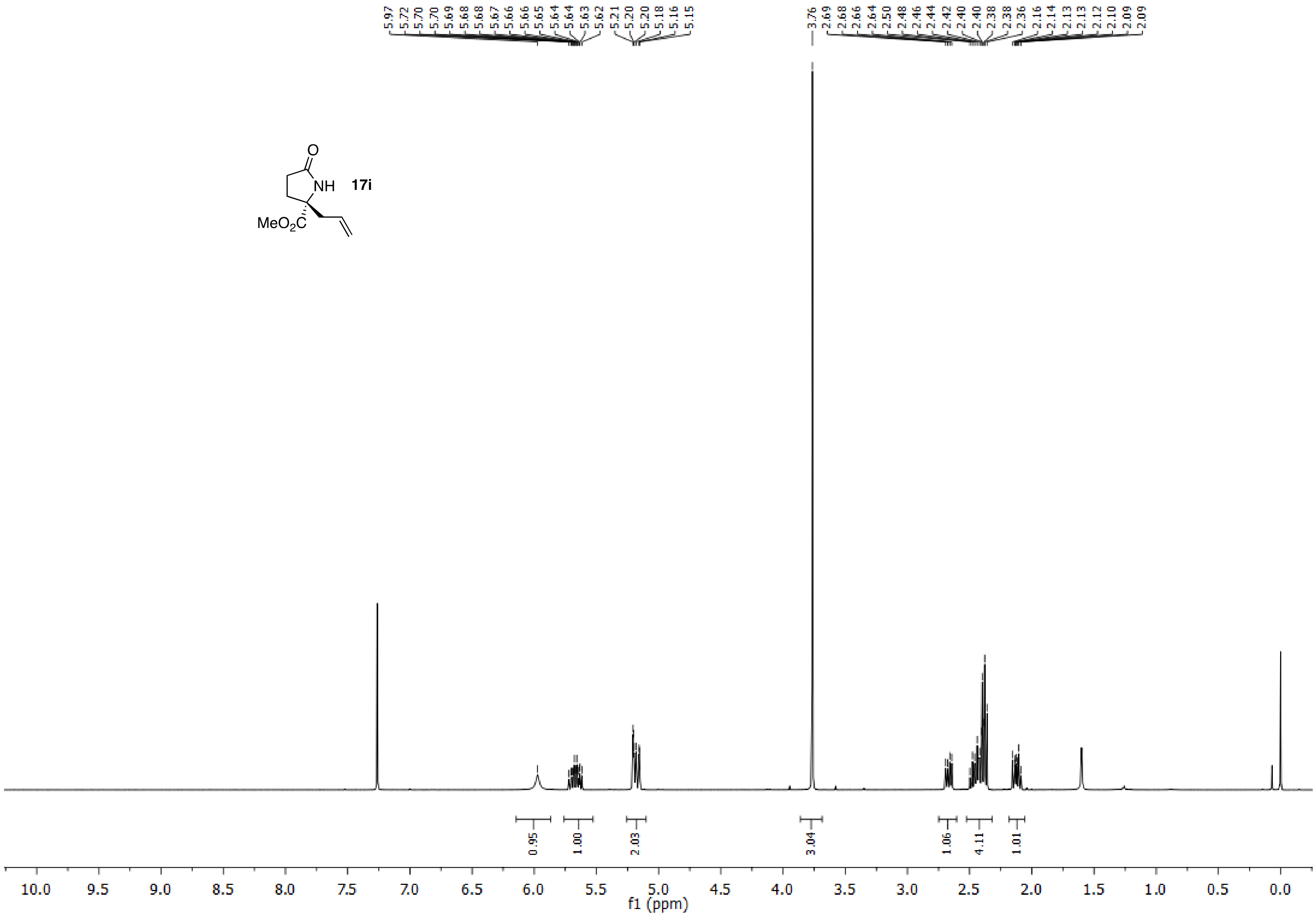
5.97 5.72 5.70 5.70 5.69 5.68 5.68 5.67 5.66 5.66 5.65 5.64 5.64 5.63 5.62 5.21 5.20 5.20 5.18 5.16 5.15
3.76 2.69 2.68 2.66 2.64 2.50 2.48 2.46 2.44 2.42 2.40 2.40 2.38 2.38 2.36 2.16 2.14 2.13 2.13 2.12 2.10 2.09 2.09
MeO2C
NH
O
17i
0.95
1.00
2.03
3.04
1.06
4.11
1.01
10.0 9.5 9.0 8.5 8.0 7.5 7.0 6.5 6.0 5.5 5.0 4.5 4.0 3.5 3.0 2.5 2.0 1.5 1.0 0.5 0.0
f1 (ppm)

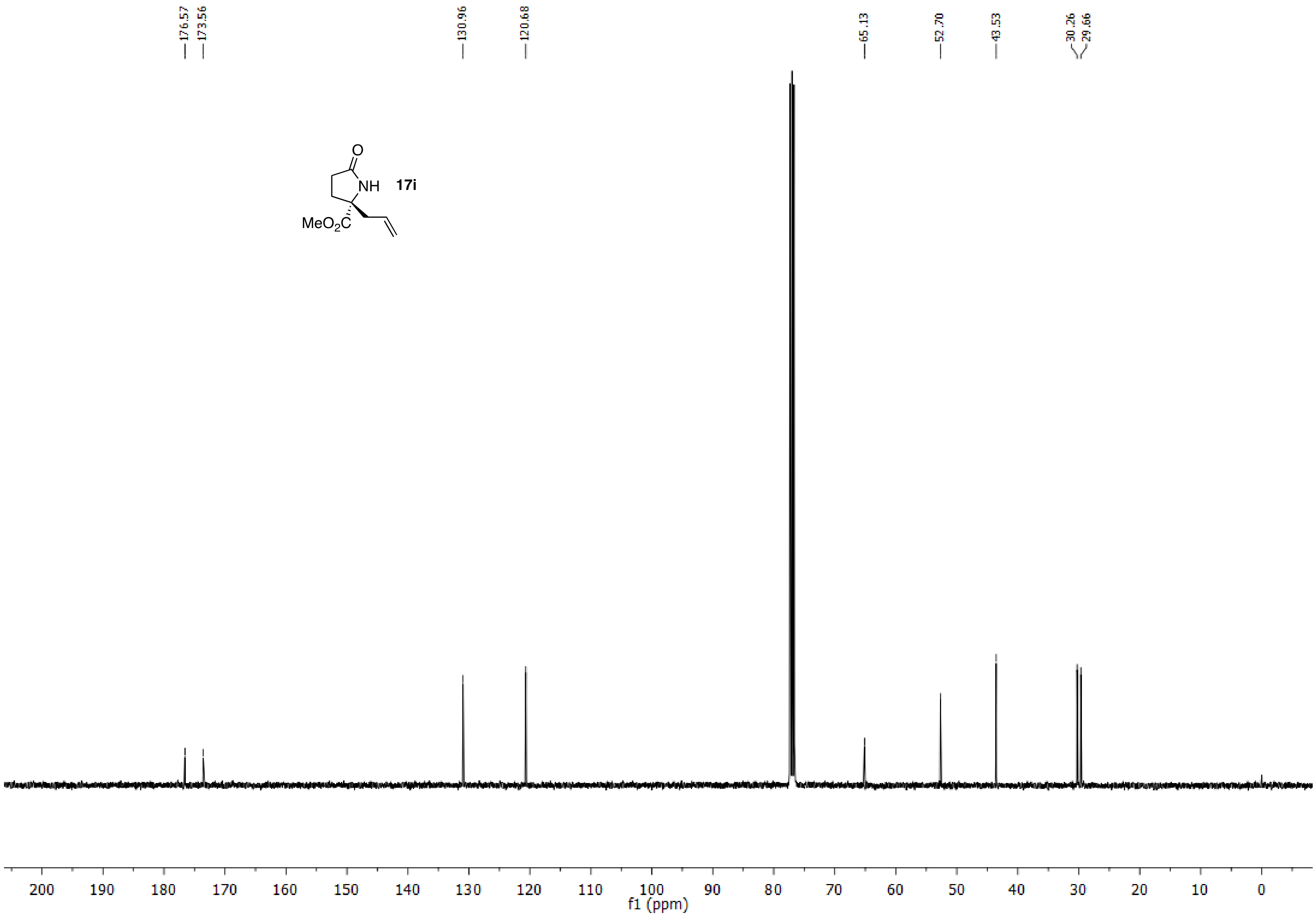

176.57
173.56
130.96
120.68
65.13
52.70
43.53
30.26
29.66
O
NH
17i
MeO2C
200 190 180 170 160 150 140 130 120 110 100 90 80 70 60 50 40 30 20 10 0
f1 (ppm)

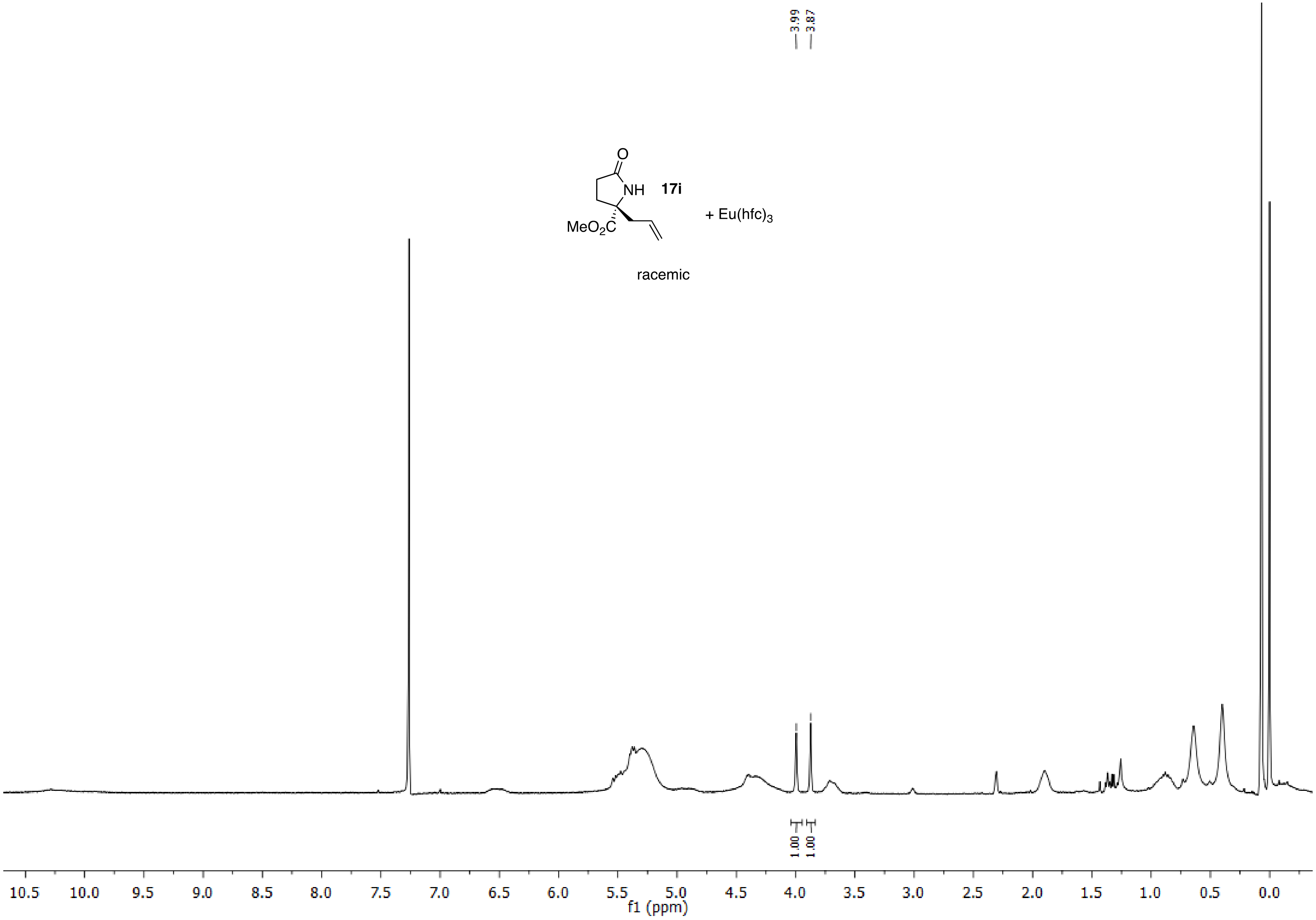

3.99
3.87
O
NH
17i
MeO2C
+ Eu(hfc)3
racemic
1.00
1.00
10.5 10.0 9.5 9.0 8.5 8.0 7.5 7.0 6.5 6.0 5.5 5.0 4.5 4.0 3.5 3.0 2.5 2.0 1.5 1.0 0.5 0.0
f1 (ppm)

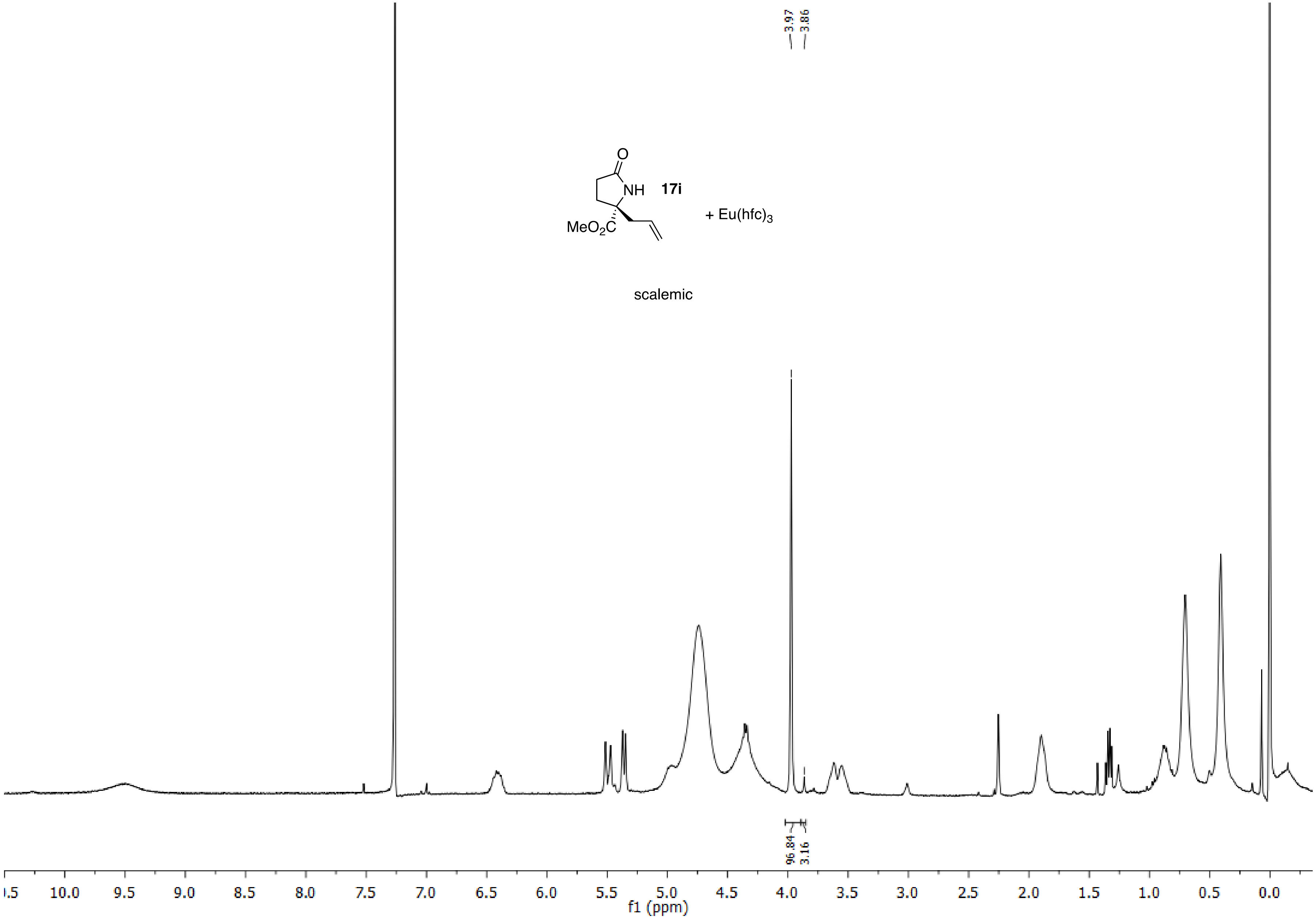
3.97
3.86
O
NH
17i
MeO2C
+ Eu(hfc)3
scalemic
96.84
3.16
10.0 9.5 9.0 8.5 8.0 7.5 7.0 6.5 6.0 5.5 5.0 4.5 4.0 3.5 3.0 2.5 2.0 1.5 1.0 0.5 0.0
f1 (ppm)

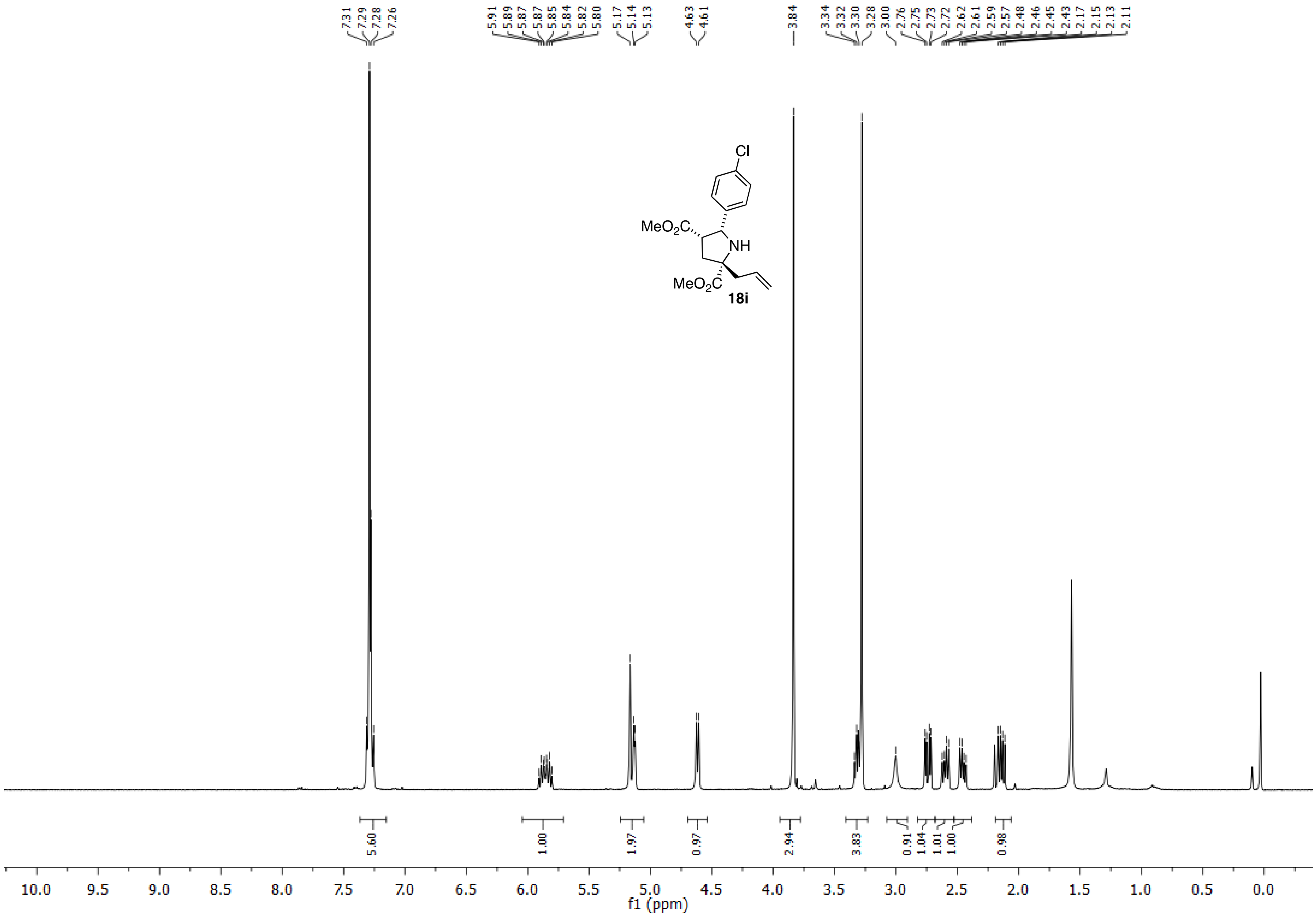
7.31 7.29 7.28 7.26
5.91 5.89 5.87 5.87 5.85 5.84 5.82 5.80
5.17 5.14 5.13
4.63 4.61
3.84
3.34 3.32 3.30 3.28 3.00 2.76 2.75 2.73 2.72 2.62 2.61 2.59 2.57 2.48 2.46 2.45 2.43 2.17 2.15 2.13 2.11
Cl
MeO2C
NH
MeO2C
18i
5.60
1.00
1.97
0.97
2.94
3.83
0.91
1.04
1.01
1.00
0.98
10.0 9.5 9.0 8.5 8.0 7.5 7.0 6.5 6.0 5.5 5.0 4.5 4.0 3.5 3.0 2.5 2.0 1.5 1.0 0.5 0.0
f1 (ppm)

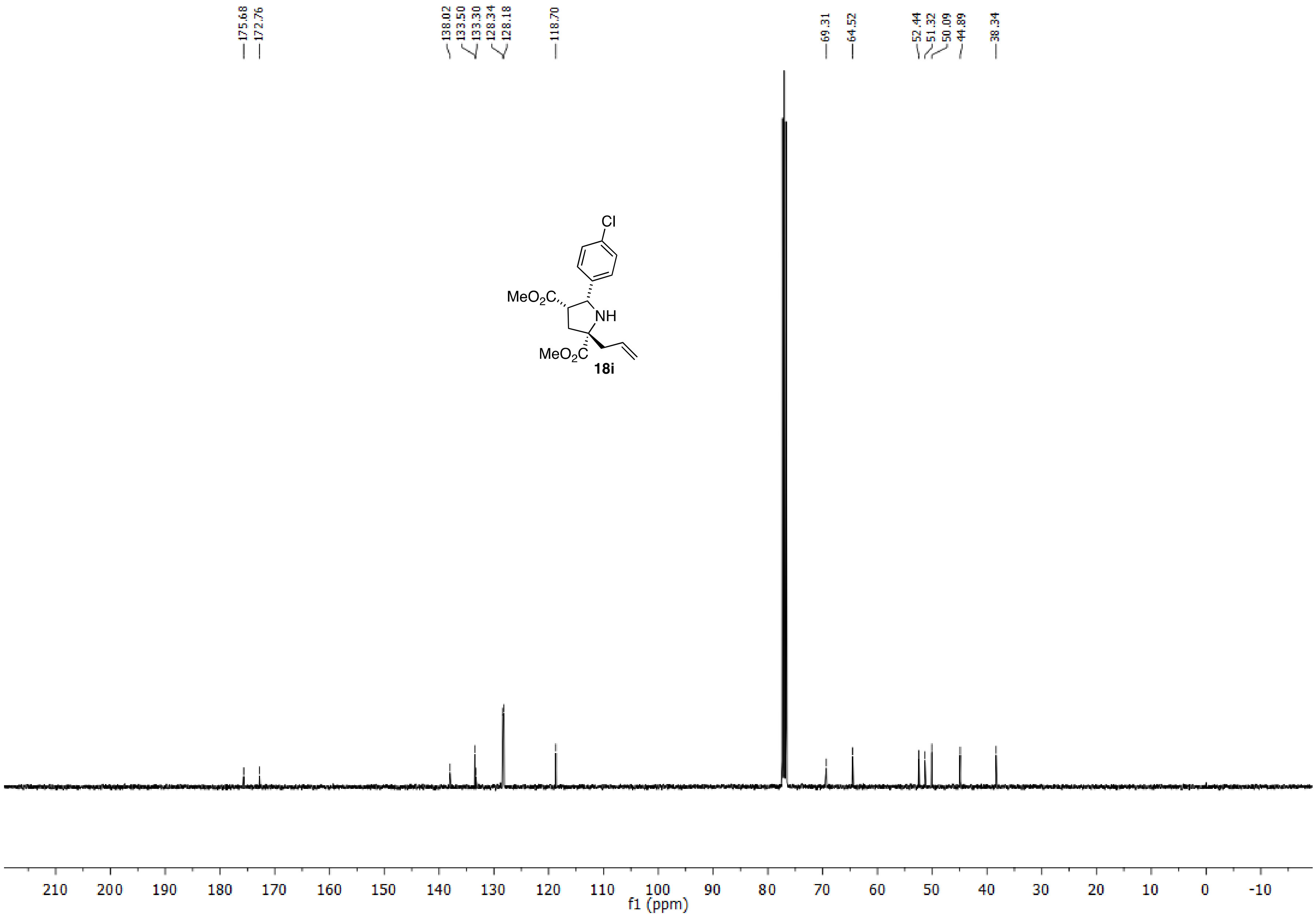
175.68
172.76
138.02
133.50
133.30
128.34
128.18
118.70
69.31
64.52
52.44
51.32
50.09
44.89
38.34
Cl
MeO2C
NH
MeO2C
18i
210 200 190 180 170 160 150 140 130 120 110 100 90 80 70 60 50 40 30 20 10 0 -10
f1 (ppm)

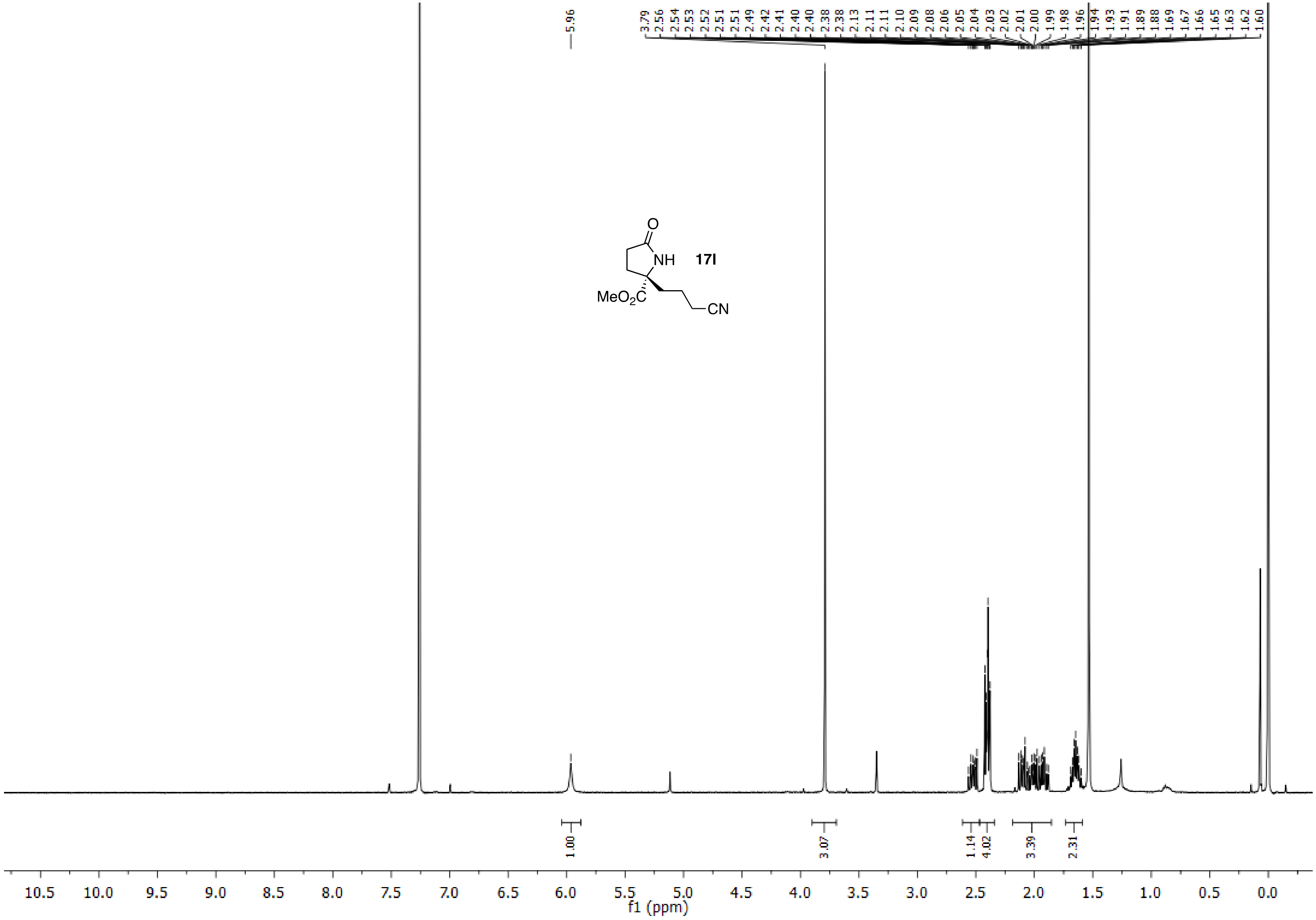

5.96
3.79 2.56 2.54 2.53 2.52 2.51 2.51 2.49 2.42 2.41 2.40 2.40 2.38 2.38 2.13 2.11 2.11 2.10 2.09 2.08 2.06 2.05 2.04 2.03 2.02 2.01 2.00 1.99 1.98 1.96 1.94 1.93 1.91 1.89 1.88 1.69 1.67 1.66 1.65 1.63 1.62 1.60
O
NH
17l
MeO2C
CN
1.00
3.07
1.14
4.02
3.39
2.31
10.5 10.0 9.5 9.0 8.5 8.0 7.5 7.0 6.5 6.0 5.5 5.0 4.5 4.0 3.5 3.0 2.5 2.0 1.5 1.0 0.5 0.0
f1 (ppm)

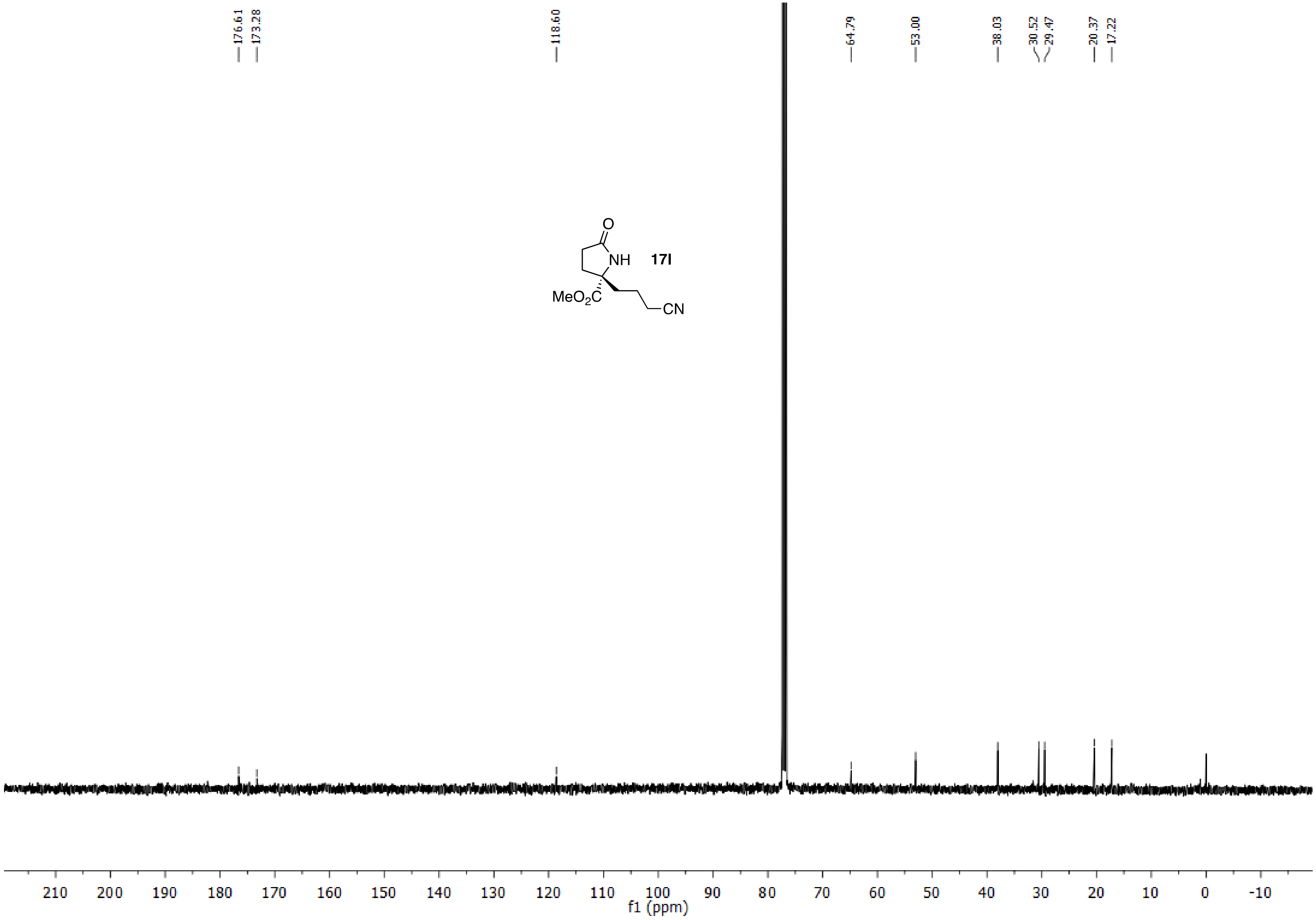
176.61
173.28
118.60
64.79
53.00
38.03
30.52
29.47
20.37
17.22
O
NH
17l
MeO2C
CN
210 200 190 180 170 160 150 140 130 120 110 100 90 80 70 60 50 40 30 20 10 0 -10
f1 (ppm)

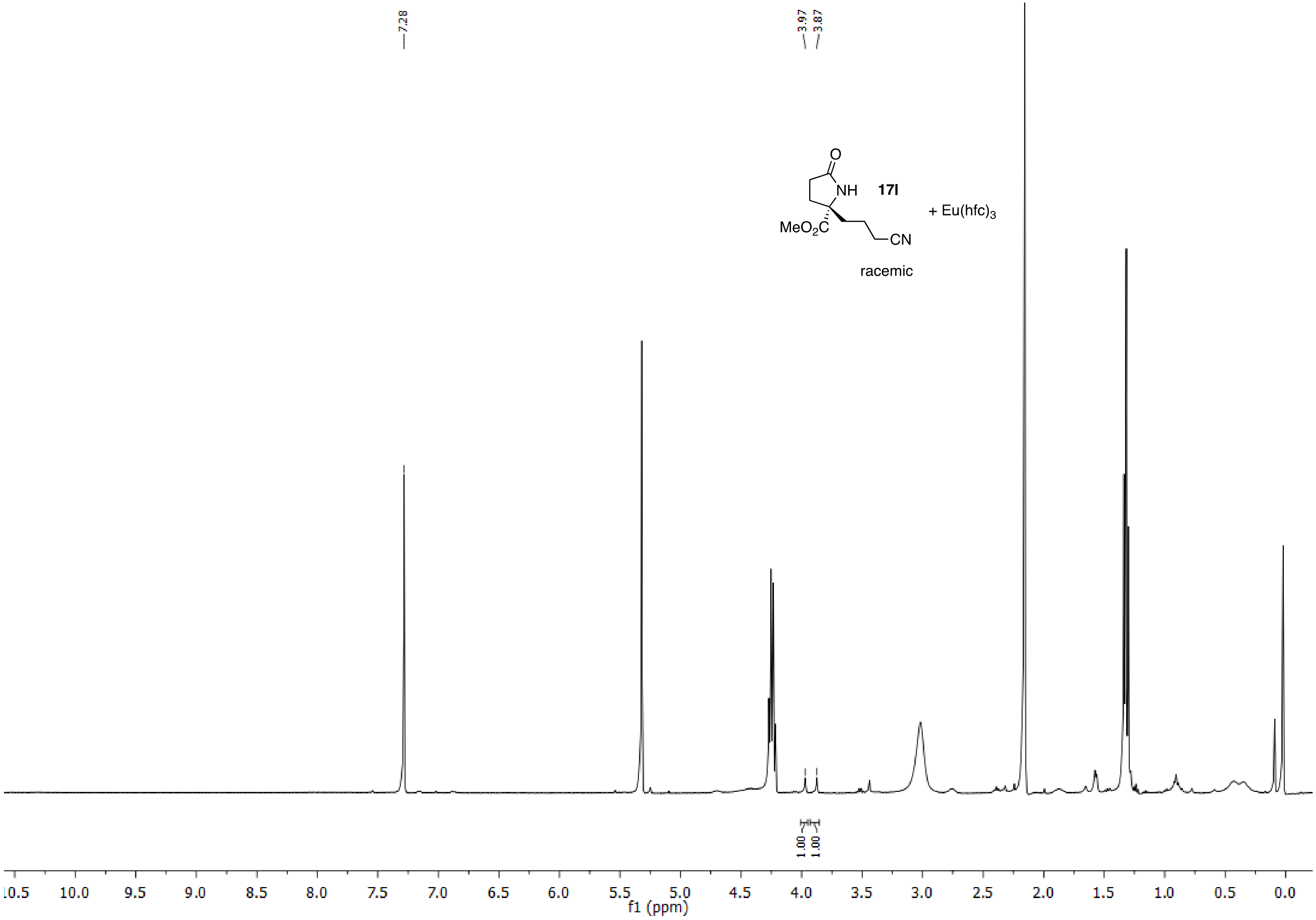
7.28
3.97
3.87
17l
+ Eu(hfc)$_3$
racemic
1.00
1.00
f1 (ppm)

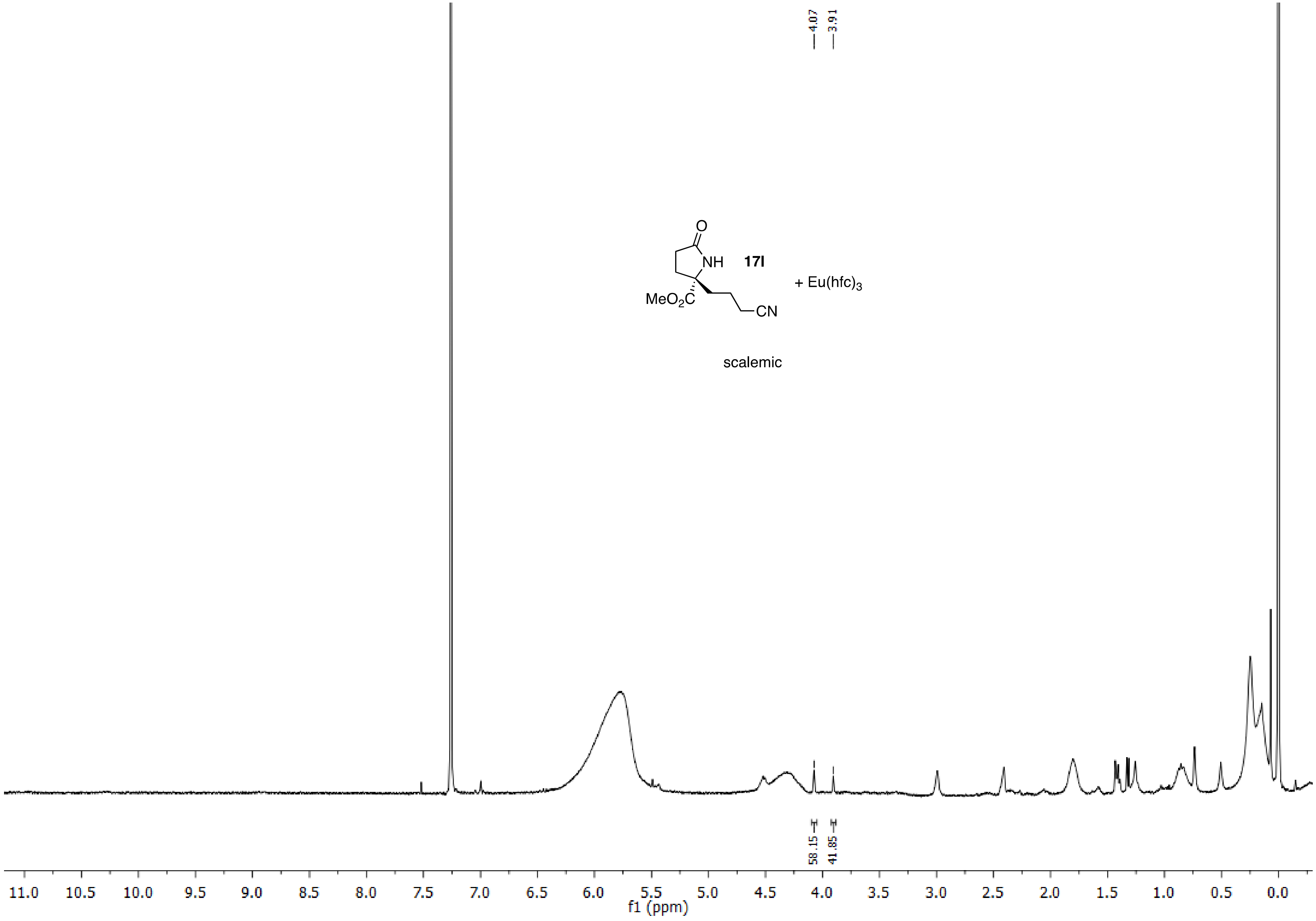

4.07
3.91
17l
MeO2C
NH
O
CN
+ Eu(hfc)3
scalemic
58.15
41.85
11.0 10.5 10.0 9.5 9.0 8.5 8.0 7.5 7.0 6.5 6.0 5.5 5.0 4.5 4.0 3.5 3.0 2.5 2.0 1.5 1.0 0.5 0.0
f1 (ppm)

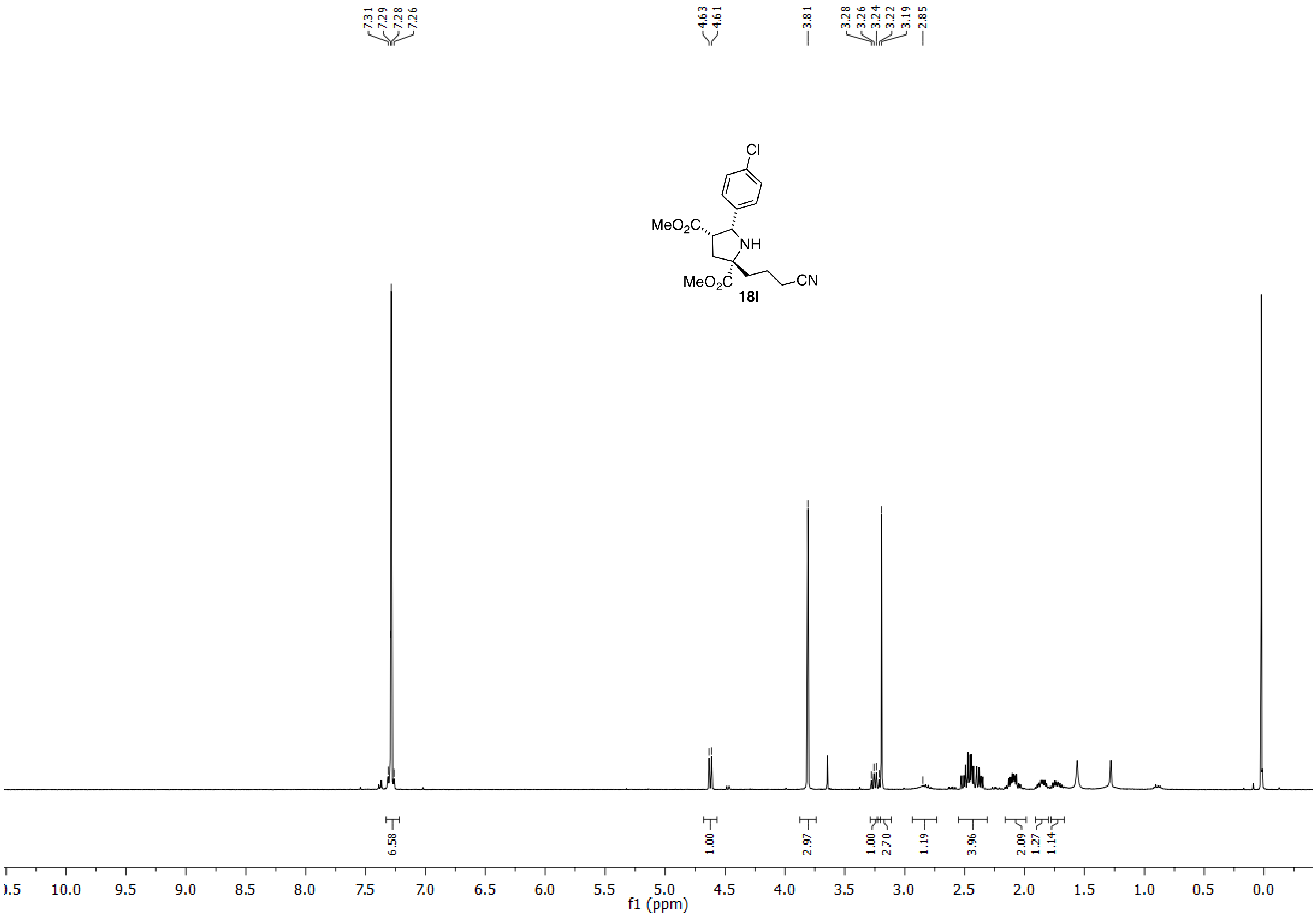
7.31
7.29
7.28
7.26
4.63
4.61
3.81
3.28
3.26
3.24
3.22
3.19
2.85
Cl
MeO2C
NH
MeO2C
CN
18l
6.58
1.00
2.97
1.00
2.70
1.19
3.96
2.09
1.27
1.14
10.0 9.5 9.0 8.5 8.0 7.5 7.0 6.5 6.0 5.5 5.0 4.5 4.0 3.5 3.0 2.5 2.0 1.5 1.0 0.5 0.0
f1 (ppm)

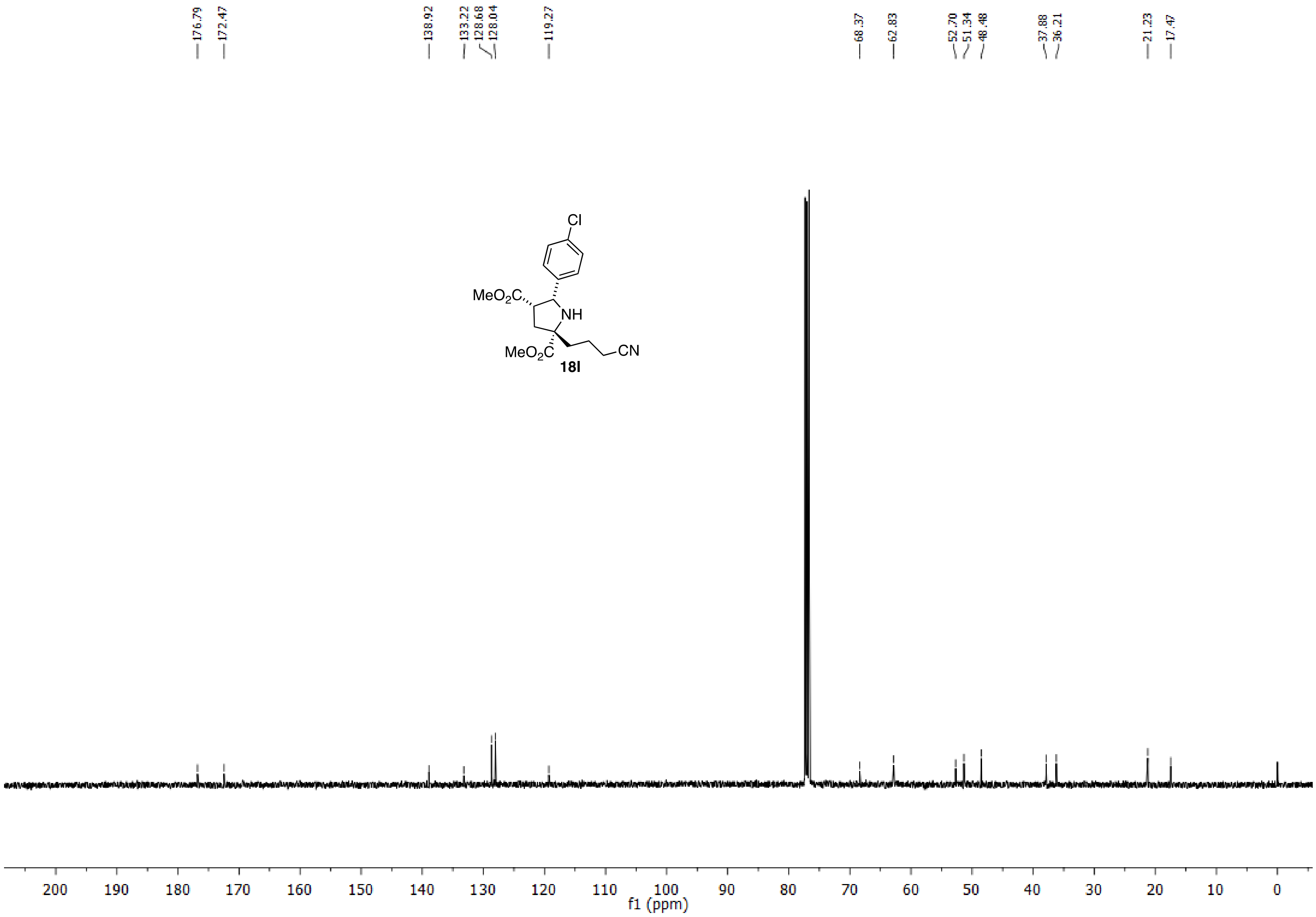
176.79
172.47
138.92
133.22
128.68
128.04
119.27
68.37
62.83
52.70
51.34
48.48
37.88
36.21
21.23
17.47
Cl
MeO2C
NH
MeO2C
CN
18l
200 190 180 170 160 150 140 130 120 110 100 90 80 70 60 50 40 30 20 10 0
f1 (ppm)

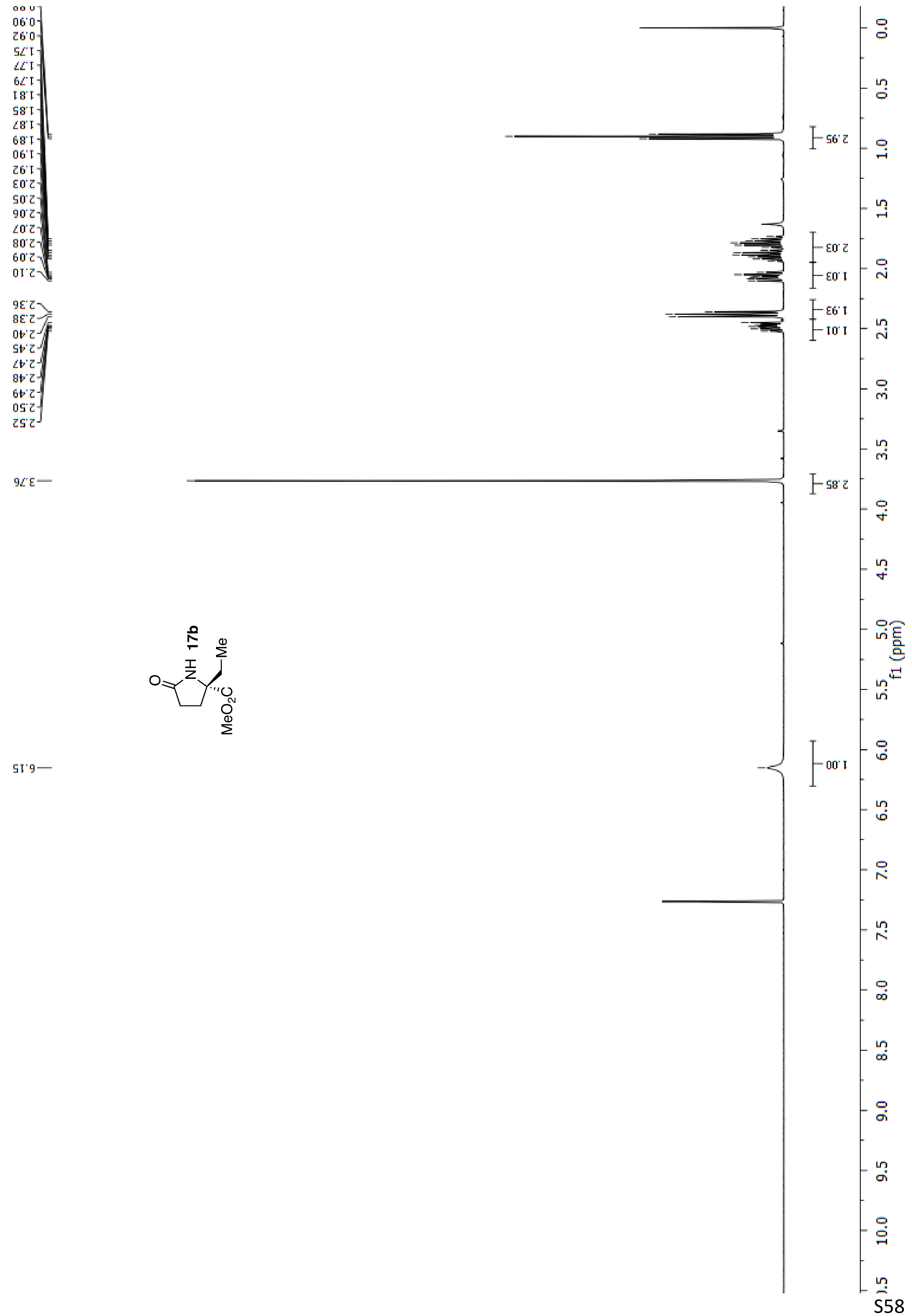
17b
NH
O
MeO2C
Me
0.90
0.92
1.75
1.77
1.79
1.81
1.85
1.87
1.89
1.90
1.92
2.03
2.05
2.06
2.07
2.08
2.09
2.10
2.36
2.38
2.40
2.45
2.47
2.48
2.49
2.50
2.52
3.76
6.15
2.95
2.03
1.03
1.93
1.01
2.85
1.00
f1 (ppm)

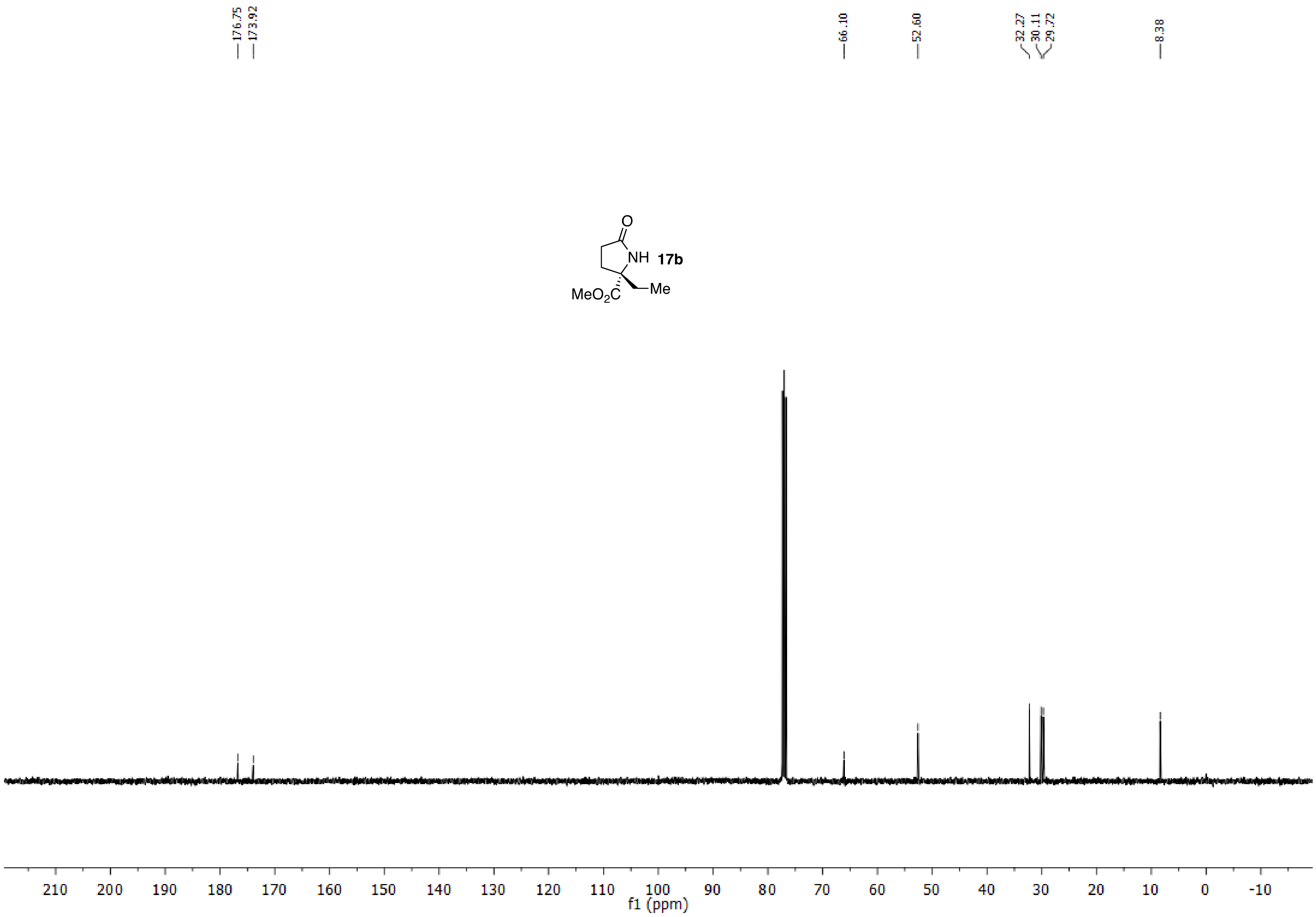
176.75
173.92
66.10
52.60
32.27
30.11
29.72
8.38
O
NH
17b
MeO2C
Me
210 200 190 180 170 160 150 140 130 120 110 100 90 80 70 60 50 40 30 20 10 0 -10
f1 (ppm)

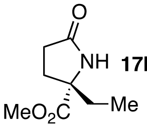

17b + Eu(hfc)$_3$

racemic

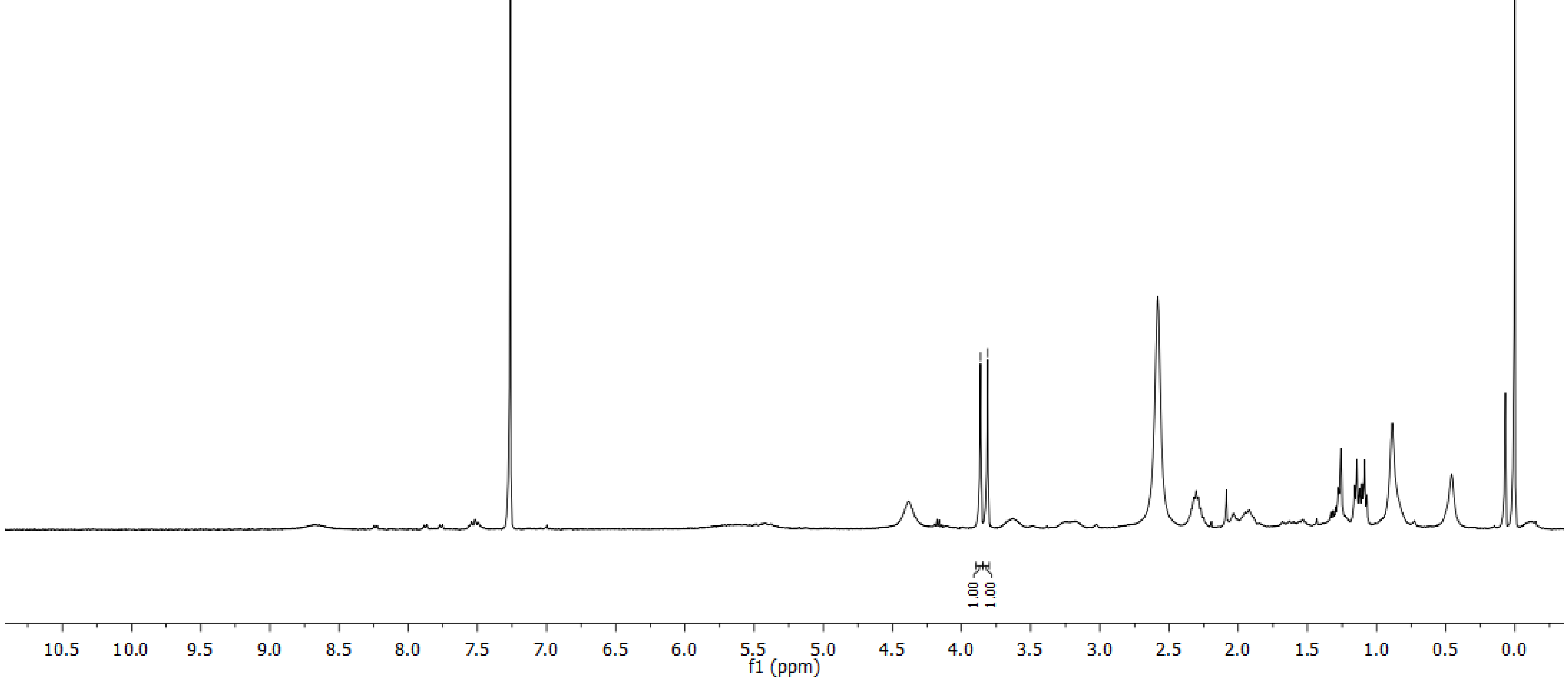

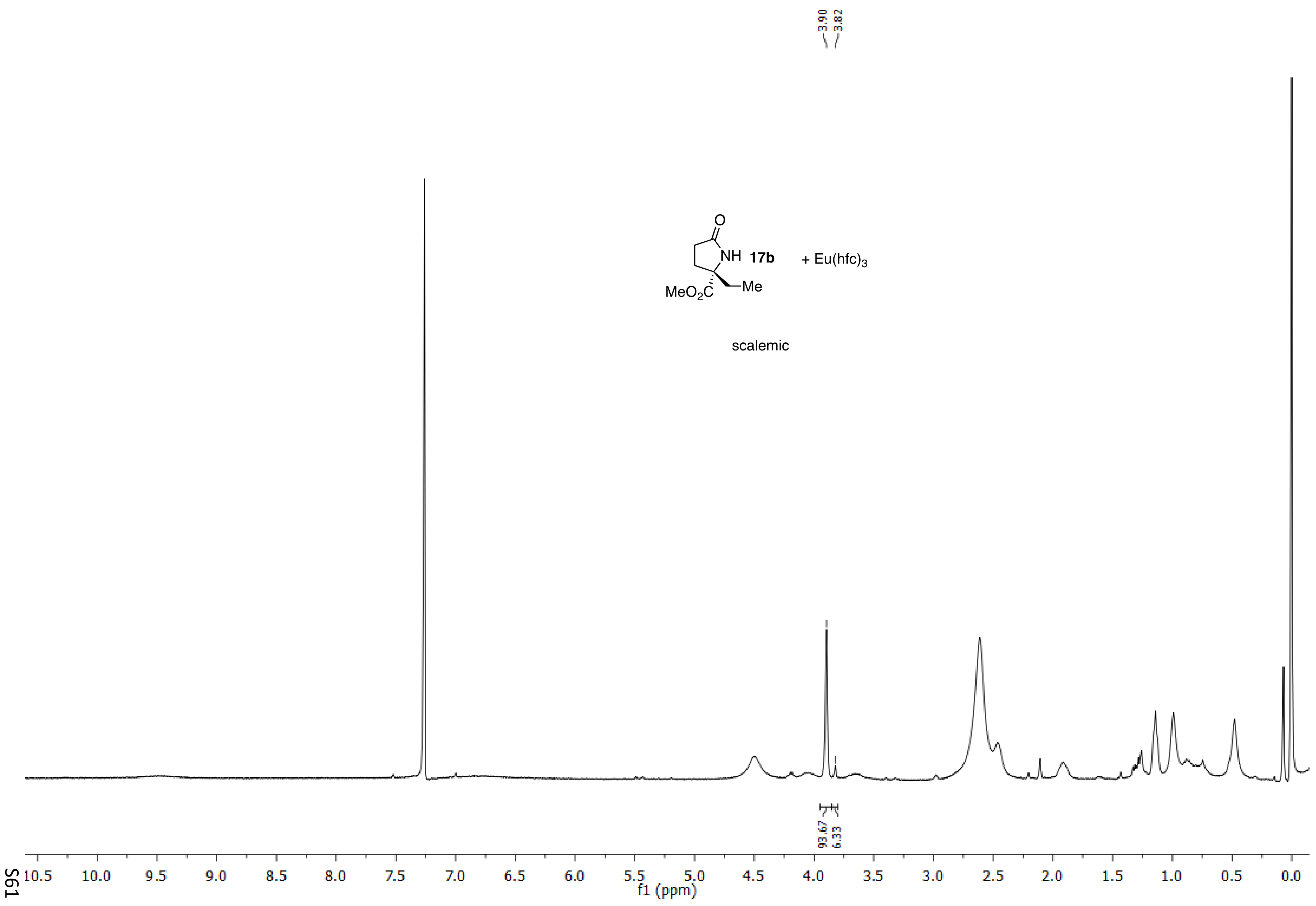
3.90
3.82
17b
+ Eu(hfc)3
MeO2C
NH
O
Me
scalemic
93.67
6.33
10.5 10.0 9.5 9.0 8.5 8.0 7.5 7.0 6.5 6.0 5.5 5.0 4.5 4.0 3.5 3.0 2.5 2.0 1.5 1.0 0.5 0.0
f1 (ppm)

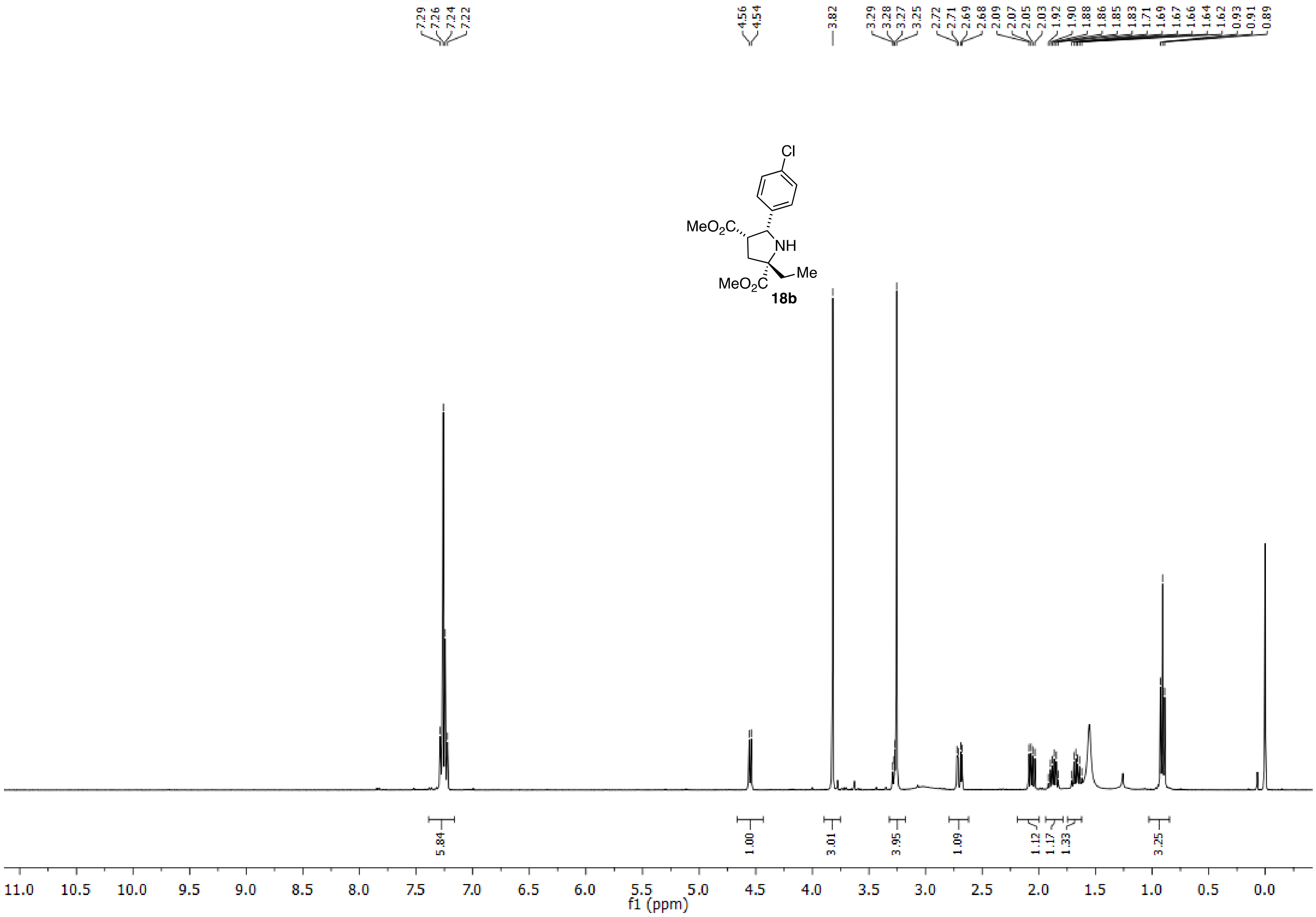
7.29
7.26
7.24
7.22
4.56
4.54
3.82
3.29
3.28
3.27
3.25
2.72
2.71
2.69
2.68
2.09
2.07
2.05
2.03
1.92
1.90
1.88
1.86
1.85
1.83
1.71
1.69
1.67
1.66
1.64
1.62
0.93
0.91
0.89
Cl
MeO2C
NH
Me
MeO2C
18b
5.84
1.00
3.01
3.95
1.09
1.12
1.17
1.33
3.25
11.0
10.5
10.0
9.5
9.0
8.5
8.0
7.5
7.0
6.5
6.0
5.5
5.0
4.5
4.0
3.5
3.0
2.5
2.0
1.5
1.0
0.5
0.0
f1 (ppm)

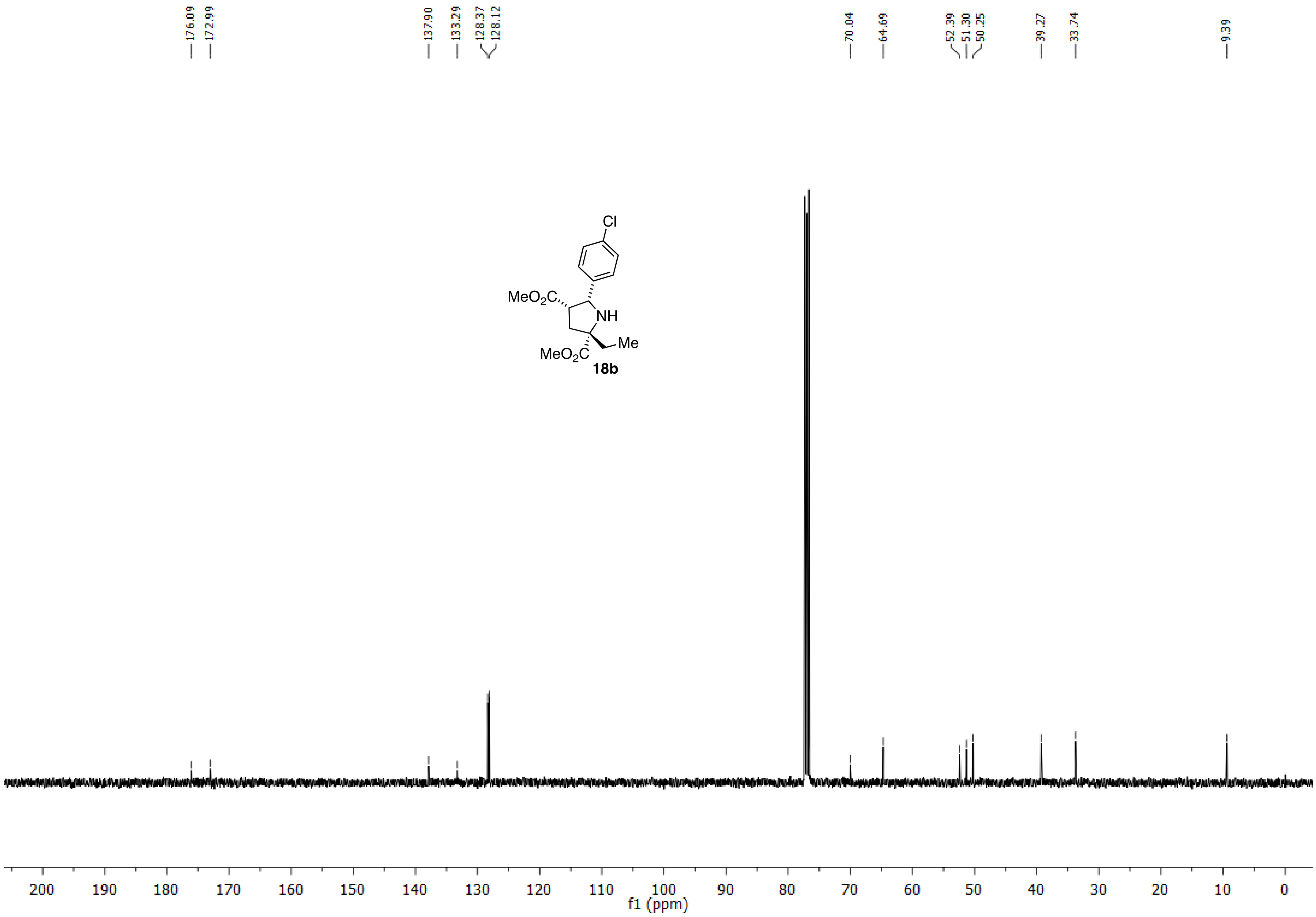
176.09
172.99
137.90
133.29
128.37
128.12
70.04
64.69
52.39
51.30
50.25
39.27
33.74
9.39
Cl
MeO2C
NH
Me
MeO2C
18b
200 190 180 170 160 150 140 130 120 110 100 90 80 70 60 50 40 30 20 10 0
f1 (ppm)

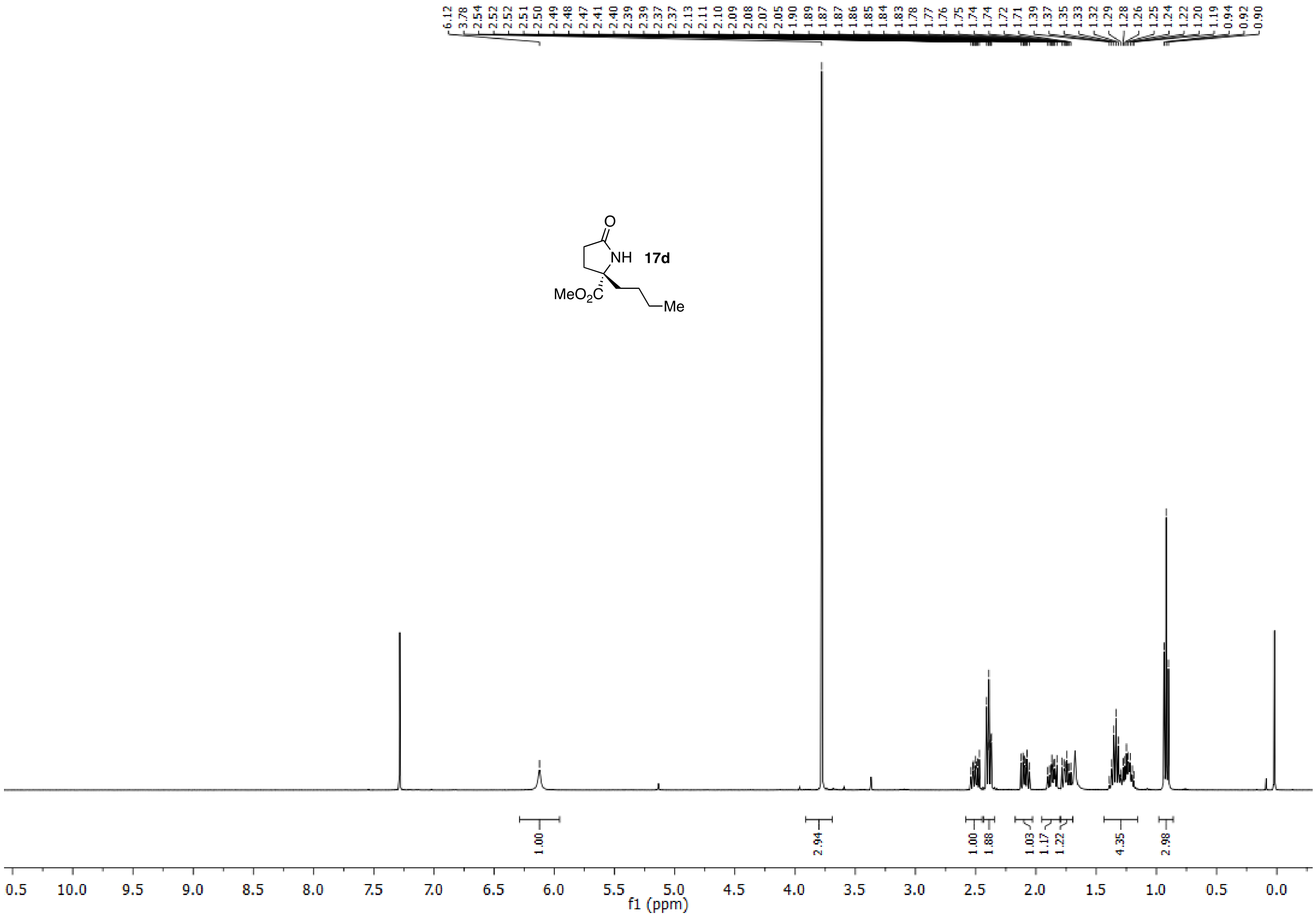
6.12 3.78 2.54 2.52 2.52 2.51 2.50 2.49 2.48 2.47 2.41 2.40 2.39 2.39 2.37 2.37 2.13 2.11 2.10 2.09 2.08 2.07 2.05 1.90 1.89 1.87 1.87 1.86 1.85 1.84 1.83 1.78 1.77 1.76 1.75 1.74 1.74 1.72 1.71 1.39 1.37 1.35 1.33 1.32 1.29 1.28 1.26 1.25 1.24 1.22 1.20 1.19 0.94 0.92 0.90
O
NH
17d
MeO_2C
Me
1.00
2.94
1.00
1.88
1.03
1.17
1.22
4.35
2.98
0.5 10.0 9.5 9.0 8.5 8.0 7.5 7.0 6.5 6.0 5.5 5.0 4.5 4.0 3.5 3.0 2.5 2.0 1.5 1.0 0.5 0.0
f1 (ppm)

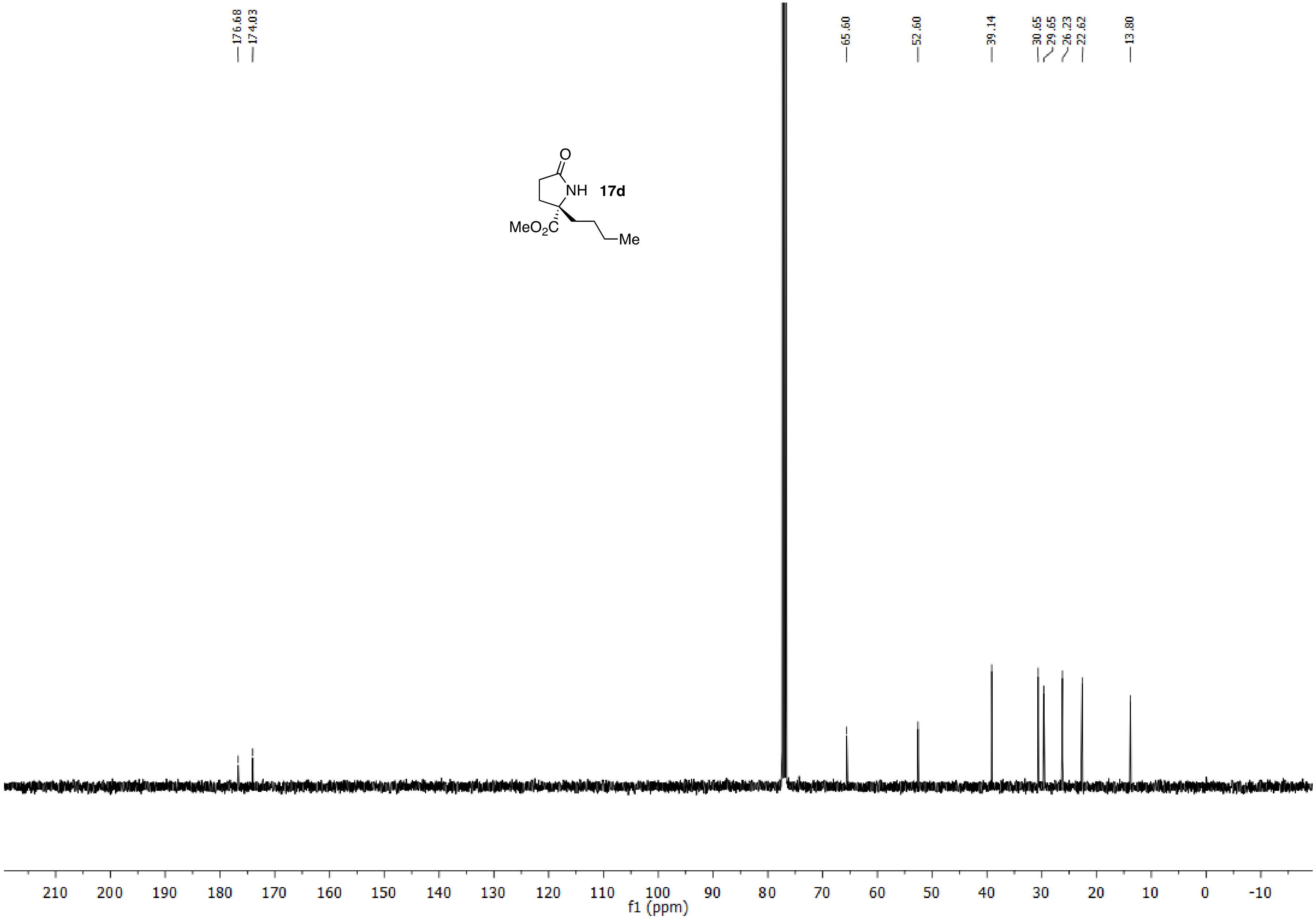
176.68
174.03
65.60
52.60
39.14
30.65
29.65
26.23
22.62
13.80
O
NH
17d
MeO2C
Me
210 200 190 180 170 160 150 140 130 120 110 100 90 80 70 60 50 40 30 20 10 0 -10
f1 (ppm)

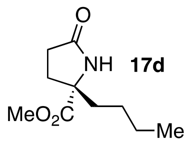

17d + Eu(hfc)$_3$

racemic

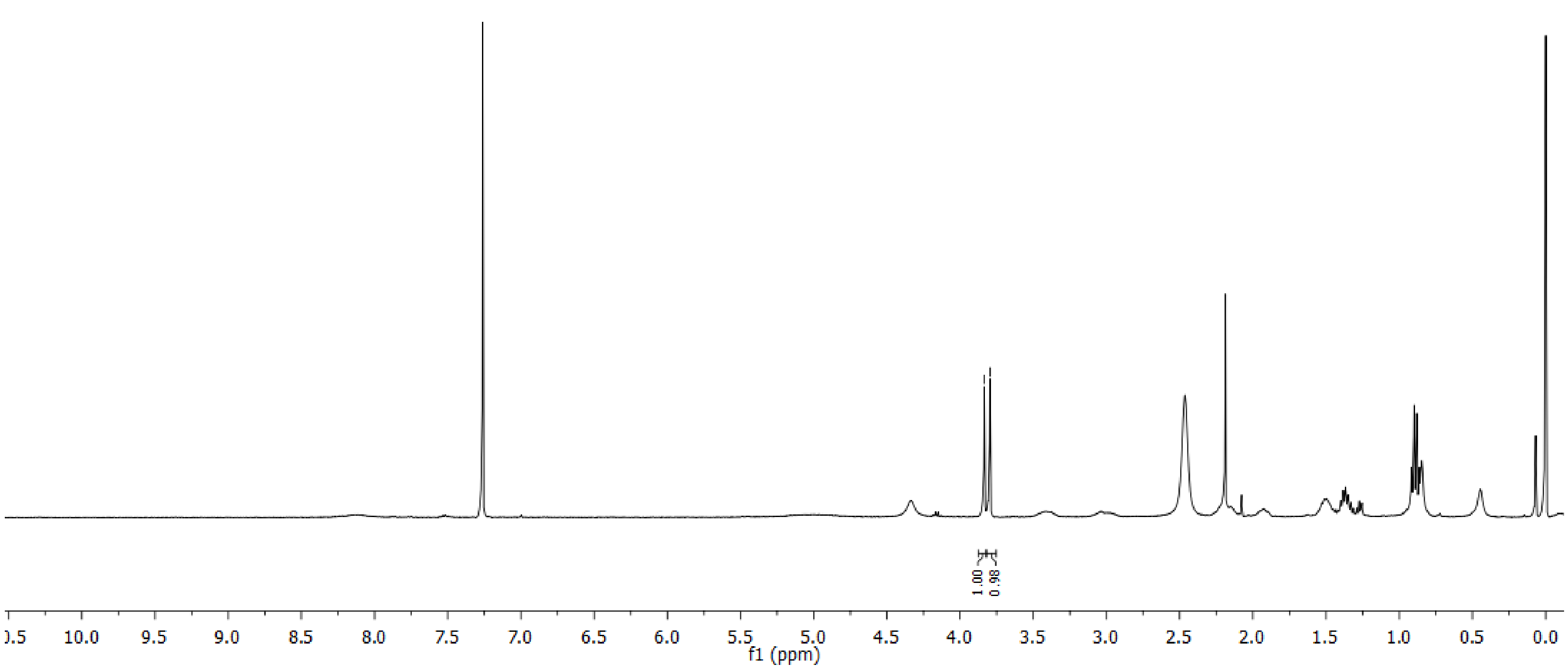

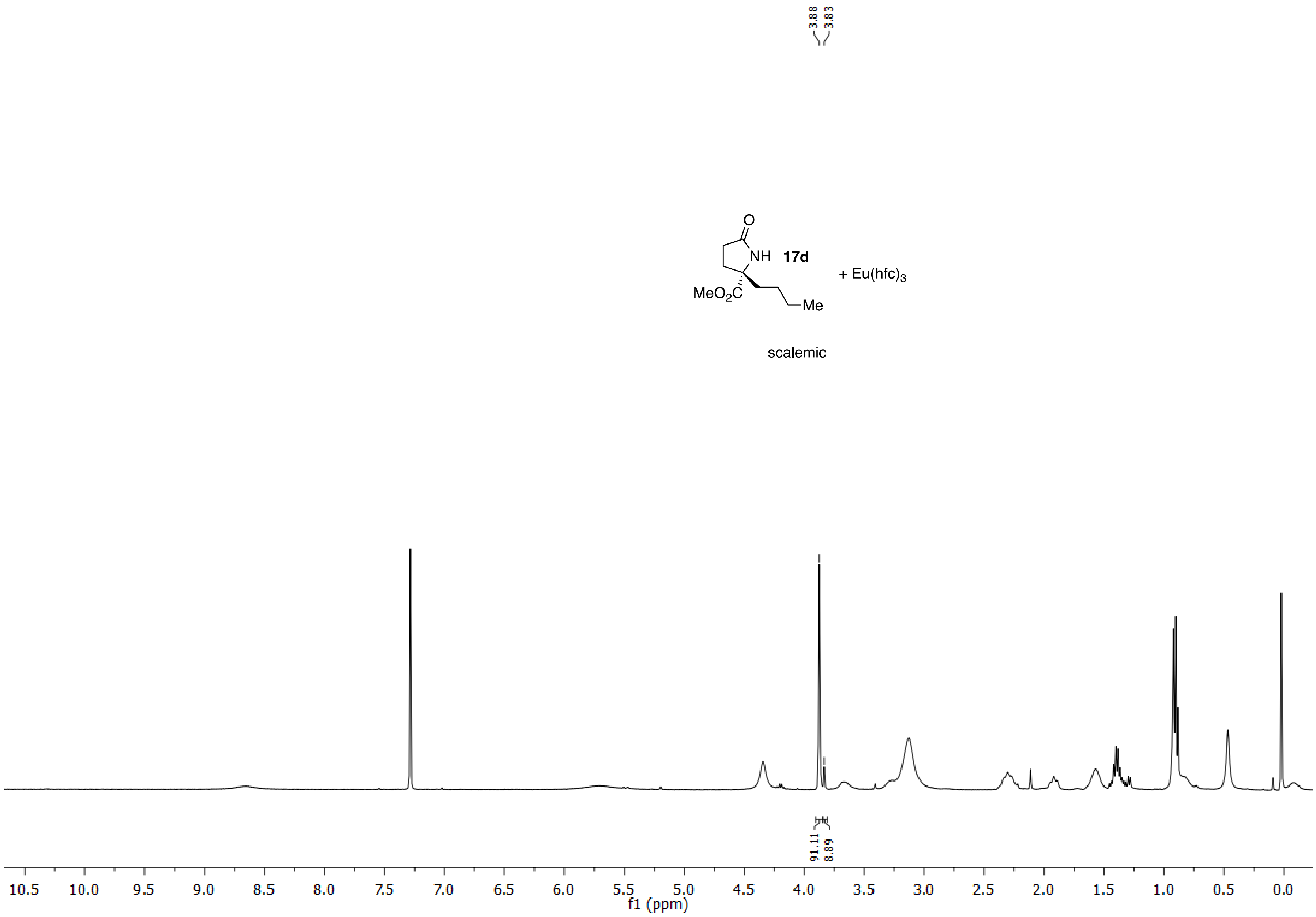
3.88
3.83
O
NH
17d
+ Eu(hfc)$_3$
MeO$_2$C
Me
scalemic
91.11
8.89
10.5 10.0 9.5 9.0 8.5 8.0 7.5 7.0 6.5 6.0 5.5 5.0 4.5 4.0 3.5 3.0 2.5 2.0 1.5 1.0 0.5 0.0
f1 (ppm)

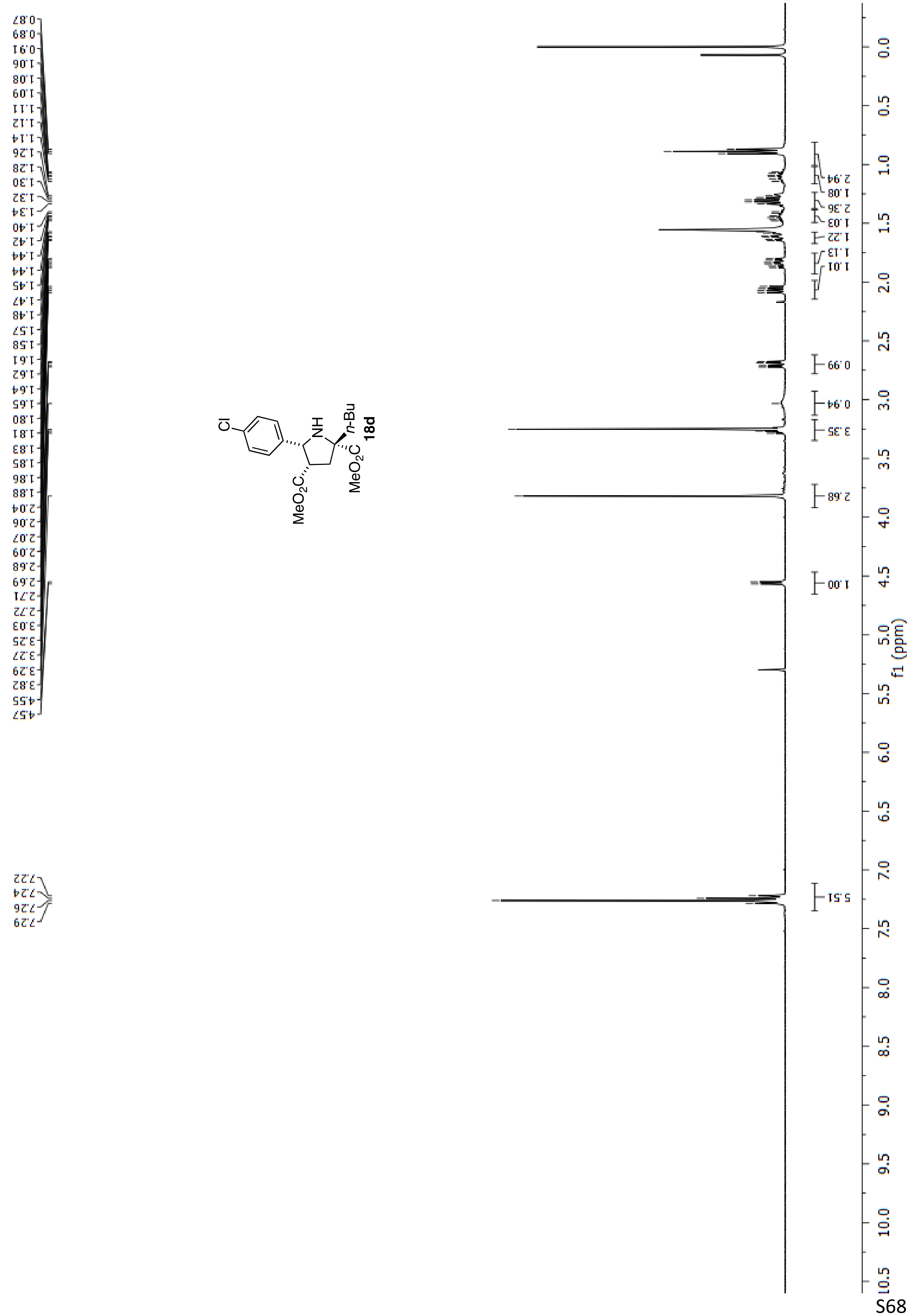
0.87
0.89
0.91
1.06
1.08
1.09
1.11
1.12
1.14
1.26
1.28
1.30
1.32
1.34
1.40
1.42
1.44
1.44
1.45
1.47
1.48
1.57
1.58
1.61
1.62
1.64
1.65
1.80
1.81
1.83
1.85
1.86
1.88
2.04
2.06
2.07
2.09
2.68
2.69
2.71
2.72
3.03
3.25
3.27
3.29
3.82
4.55
4.57
7.22
7.24
7.26
7.29
Cl
NH
n-Bu
MeO2C
MeO2C
18d
2.94
1.08
2.36
1.03
1.22
1.13
1.01
0.99
0.94
3.35
2.68
1.00
5.51
0.0
0.5
1.0
1.5
2.0
2.5
3.0
3.5
4.0
4.5
5.0
5.5
6.0
6.5
7.0
7.5
8.0
8.5
9.0
9.5
10.0
10.5
f1 (ppm)

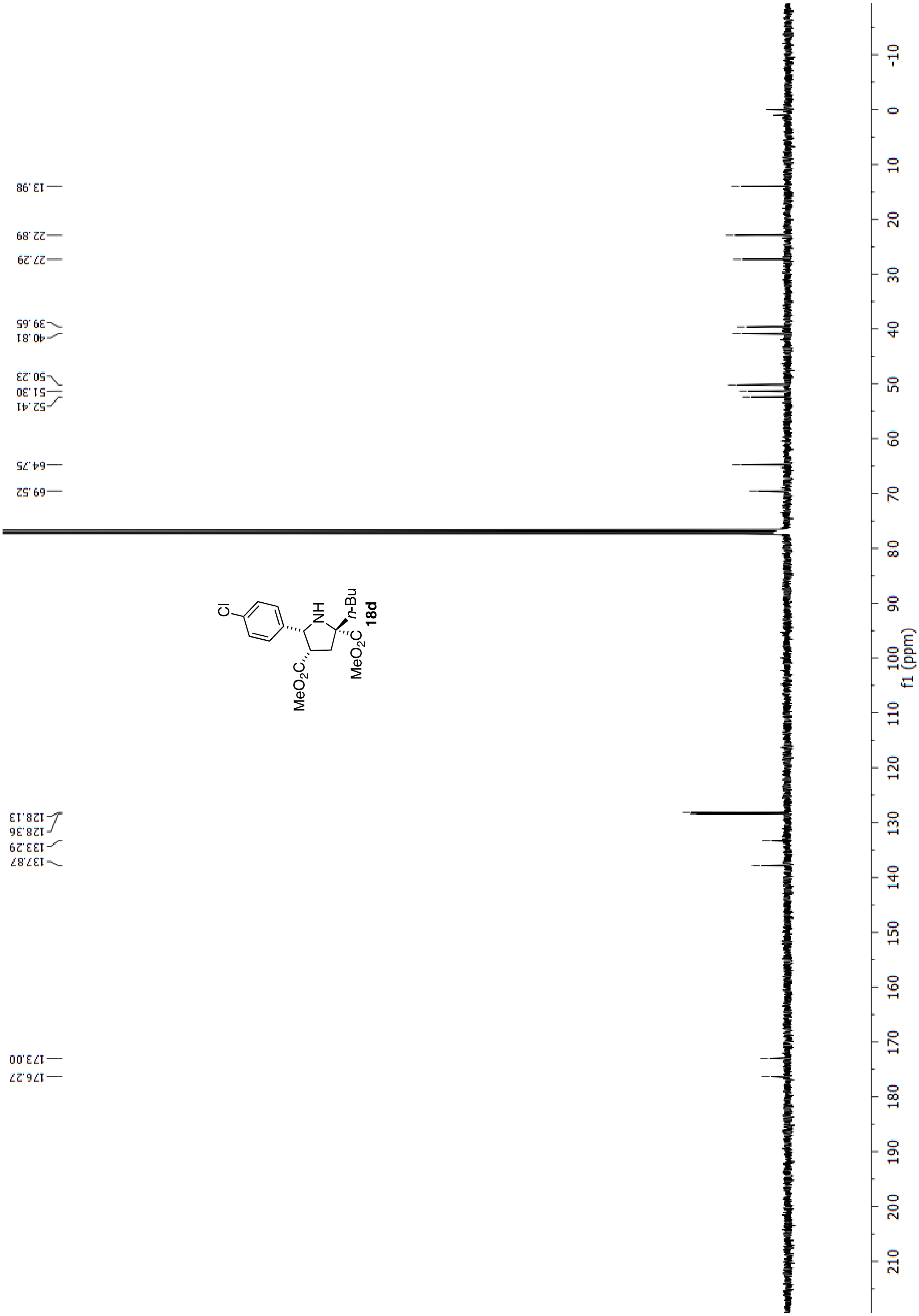
13.98
22.89
27.29
39.65
40.81
50.23
51.30
52.41
64.75
69.52
128.13
128.36
133.29
137.87
173.00
176.27
Cl
NH
n-Bu
MeO2C
MeO2C
18d
f1 (ppm)

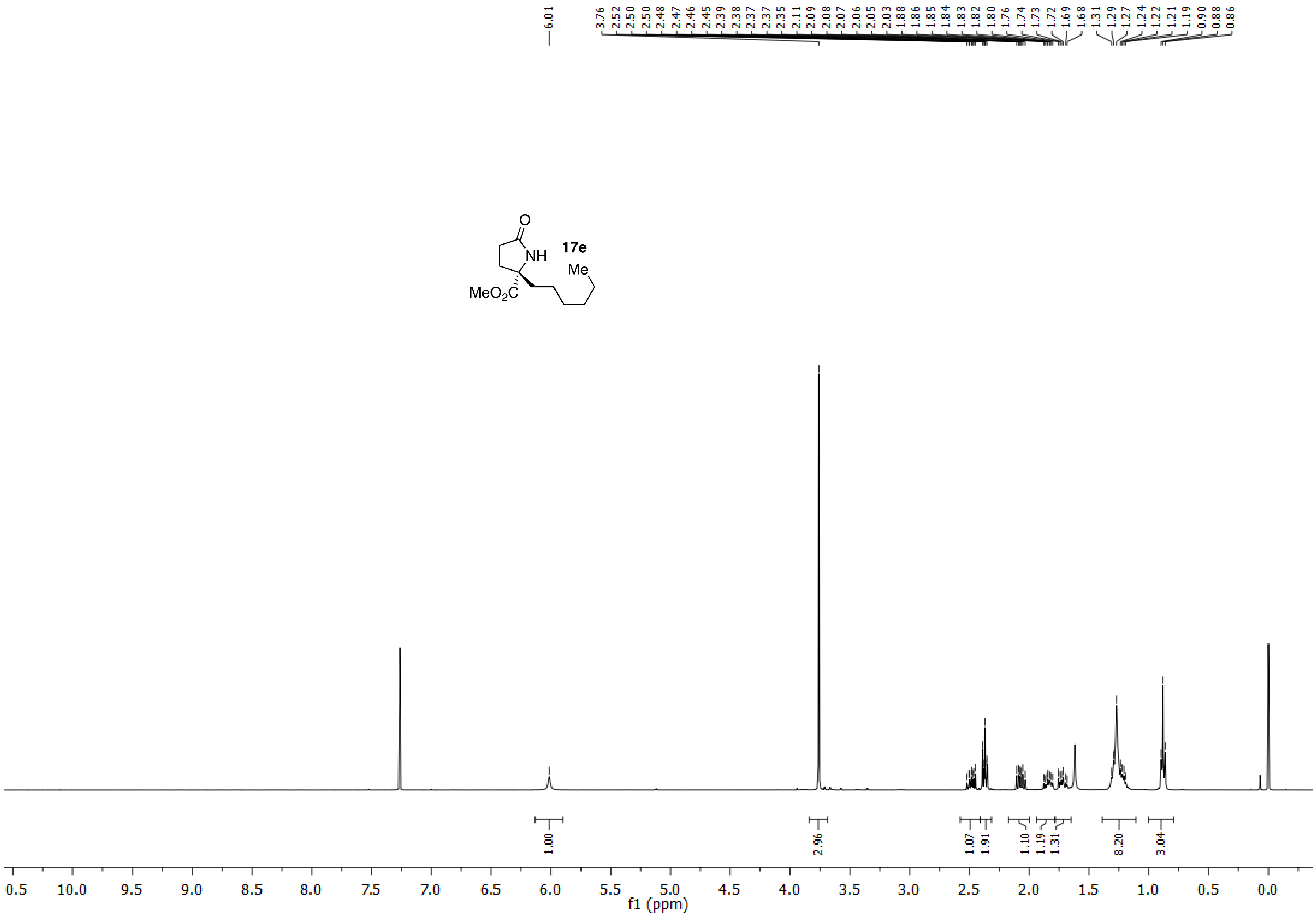
6.01
3.76 2.52 2.50 2.50 2.48 2.47 2.46 2.45 2.39 2.38 2.37 2.37 2.35 2.11 2.09 2.08 2.07 2.06 2.05 2.03 1.88 1.86 1.85 1.84 1.83 1.82 1.80 1.76 1.74 1.73 1.72 1.69 1.68 1.31 1.29 1.27 1.24 1.22 1.21 1.19 0.90 0.88 0.86
O
NH
17e
Me
MeO2C
1.00
2.96
1.07
1.91
1.10
1.19
1.31
8.20
3.04
10.5 10.0 9.5 9.0 8.5 8.0 7.5 7.0 6.5 6.0 5.5 5.0 4.5 4.0 3.5 3.0 2.5 2.0 1.5 1.0 0.5 0.0
f1 (ppm)

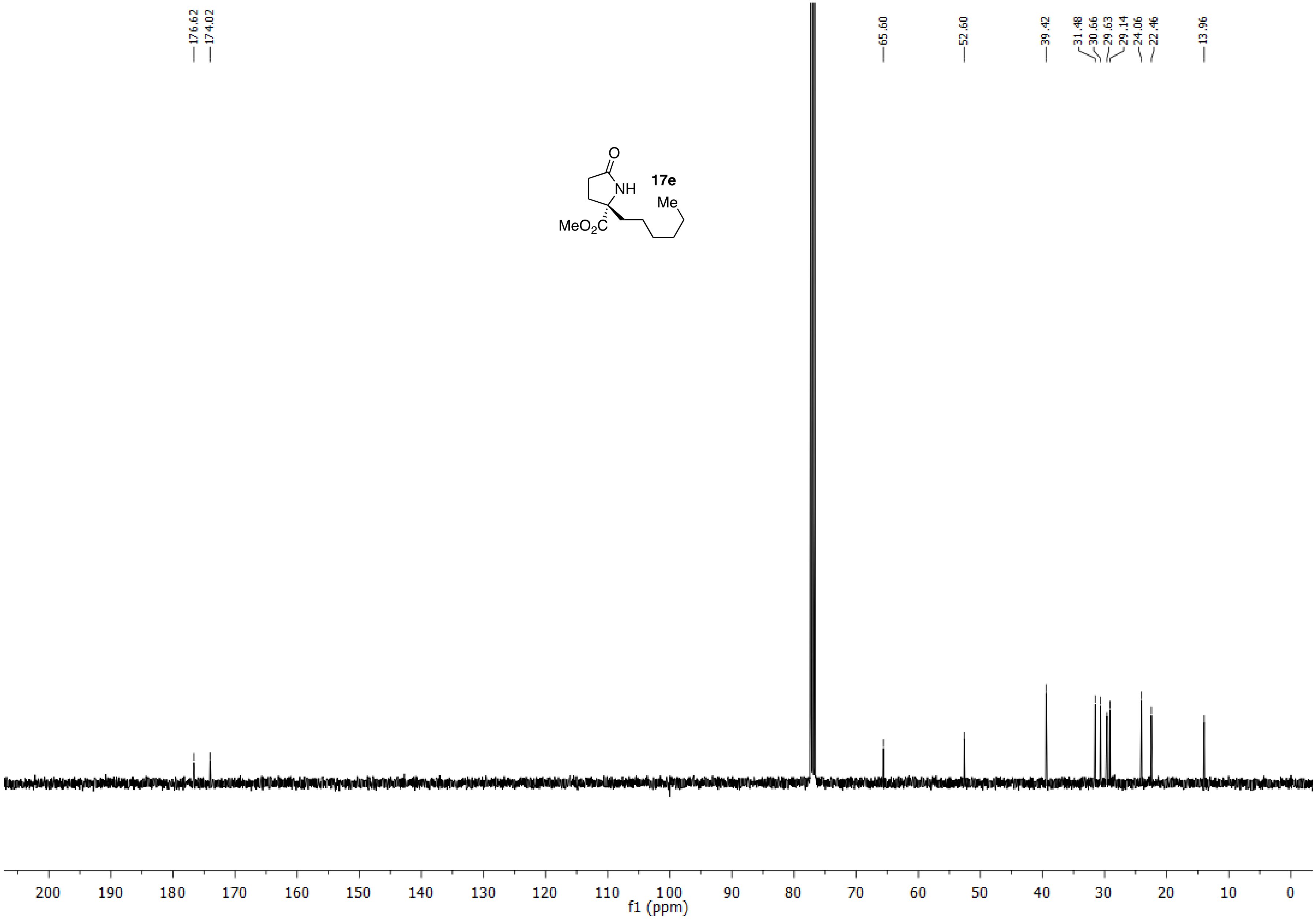
176.62
174.02
65.60
52.60
39.42
31.48
30.66
29.63
29.14
24.06
22.46
13.96
O
NH
17e
Me
MeO2C
200
190
180
170
160
150
140
130
120
110
100
90
80
70
60
50
40
30
20
10
0
f1 (ppm)

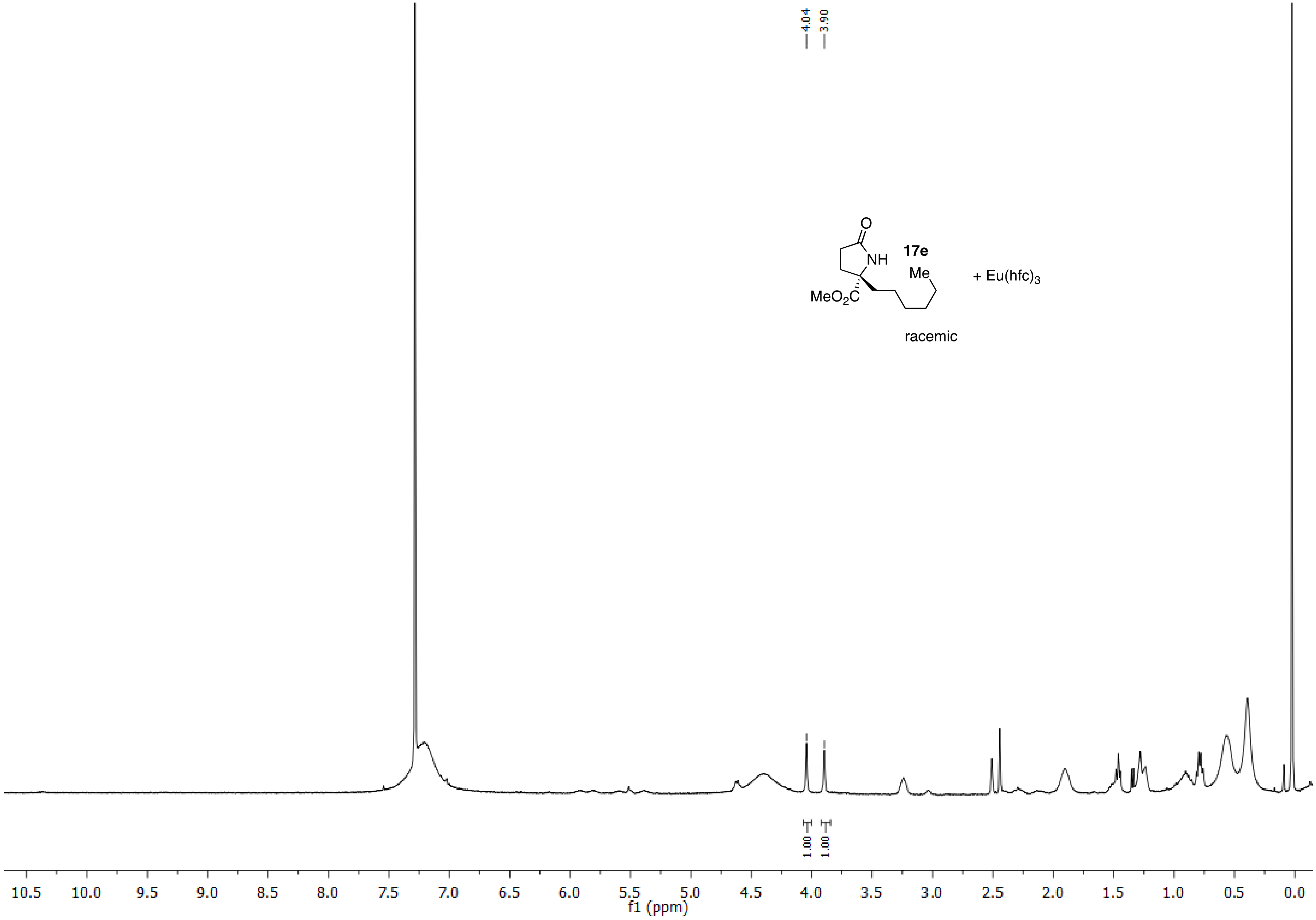

4.04
3.90
17e
NH
O
Me
MeO2C
+ Eu(hfc)3
racemic
1.00
1.00
10.5 10.0 9.5 9.0 8.5 8.0 7.5 7.0 6.5 6.0 5.5 5.0 4.5 4.0 3.5 3.0 2.5 2.0 1.5 1.0 0.5 0.0
f1 (ppm)

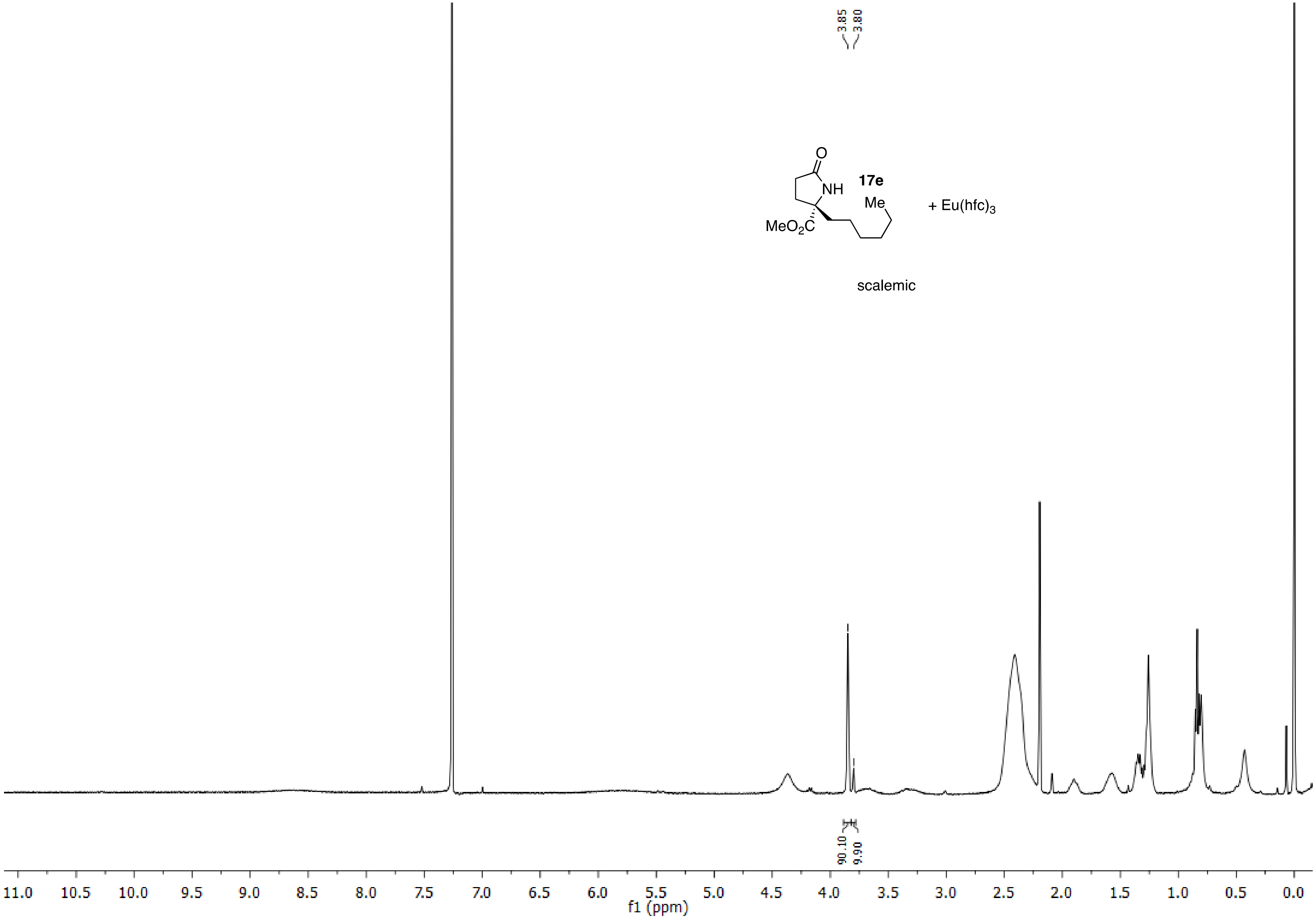
3.85
3.80
17e
NH
Me
MeO$_2$C
+ Eu(hfc)$_3$
scalemic
90.10
9.90
11.0 10.5 10.0 9.5 9.0 8.5 8.0 7.5 7.0 6.5 6.0 5.5 5.0 4.5 4.0 3.5 3.0 2.5 2.0 1.5 1.0 0.5 0.0
f1 (ppm)

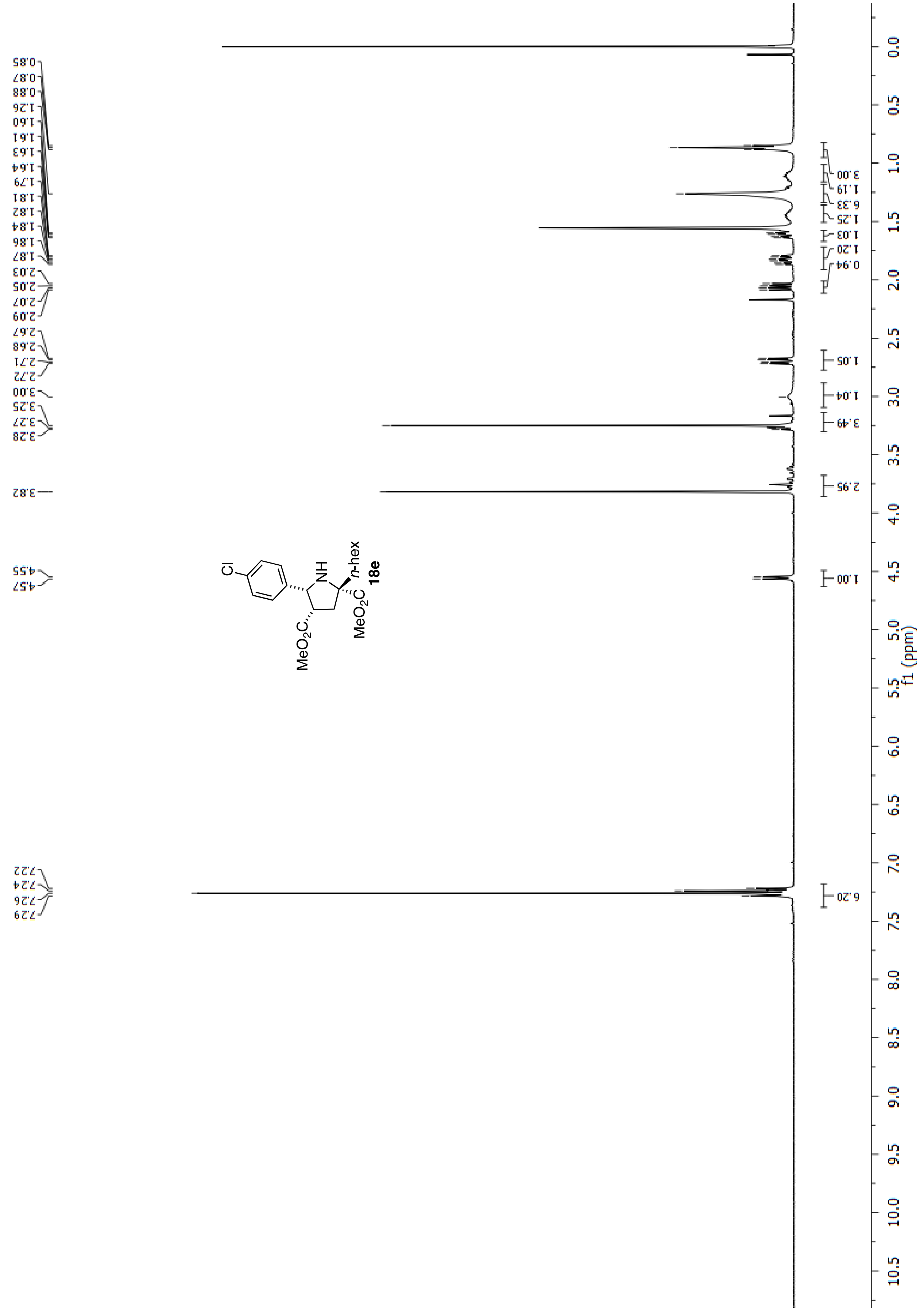

Cl
NH
n-hex
MeO2C
MeO2C
18e
f1 (ppm)

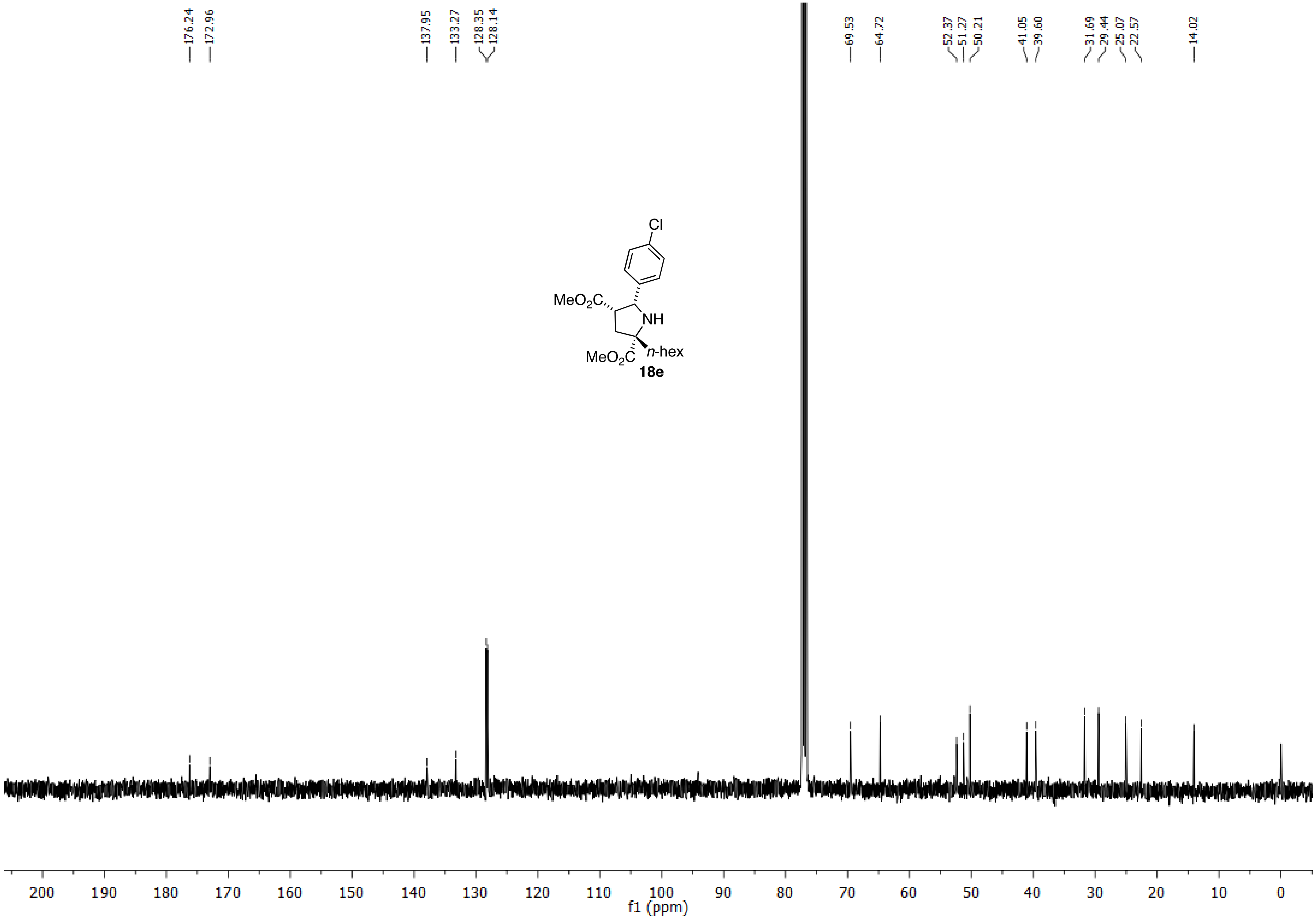
176.24
172.96
137.95
133.27
128.35
128.14
69.53
64.72
52.37
51.27
50.21
41.05
39.60
31.69
29.44
25.07
22.57
14.02
Cl
MeO2C
NH
MeO2C
n-hex
18e
200 190 180 170 160 150 140 130 120 110 100 90 80 70 60 50 40 30 20 10 0
f1 (ppm)

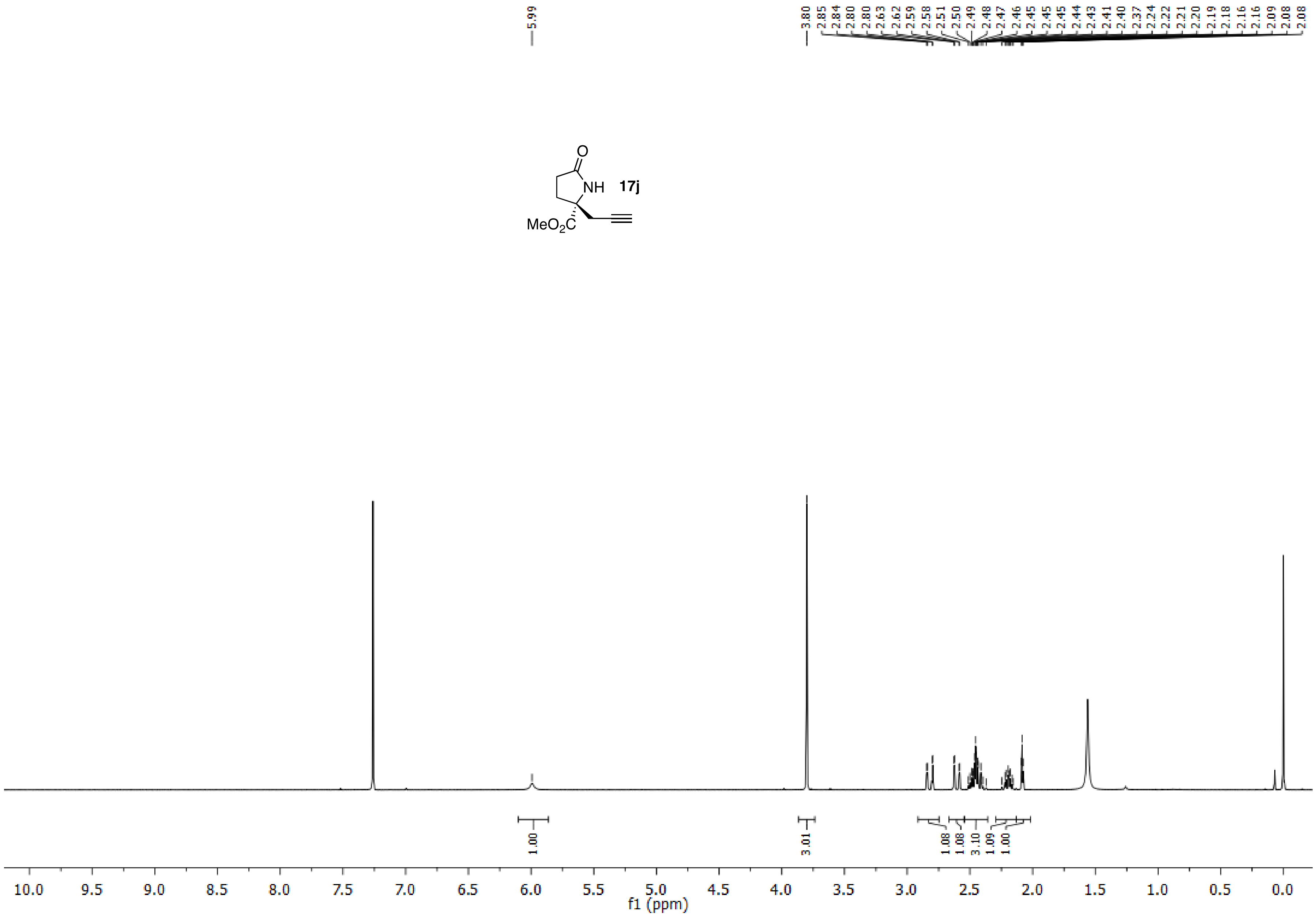
5.99
3.80
2.85
2.84
2.80
2.80
2.63
2.62
2.59
2.58
2.51
2.50
2.49
2.48
2.47
2.46
2.45
2.45
2.45
2.44
2.43
2.41
2.40
2.37
2.24
2.22
2.21
2.20
2.19
2.18
2.16
2.16
2.09
2.08
2.08
O
NH
17j
MeO2C
1.00
3.01
1.08
1.08
3.10
1.09
1.00
10.0
9.5
9.0
8.5
8.0
7.5
7.0
6.5
6.0
5.5
5.0
4.5
4.0
3.5
3.0
2.5
2.0
1.5
1.0
0.5
0.0
f1 (ppm)

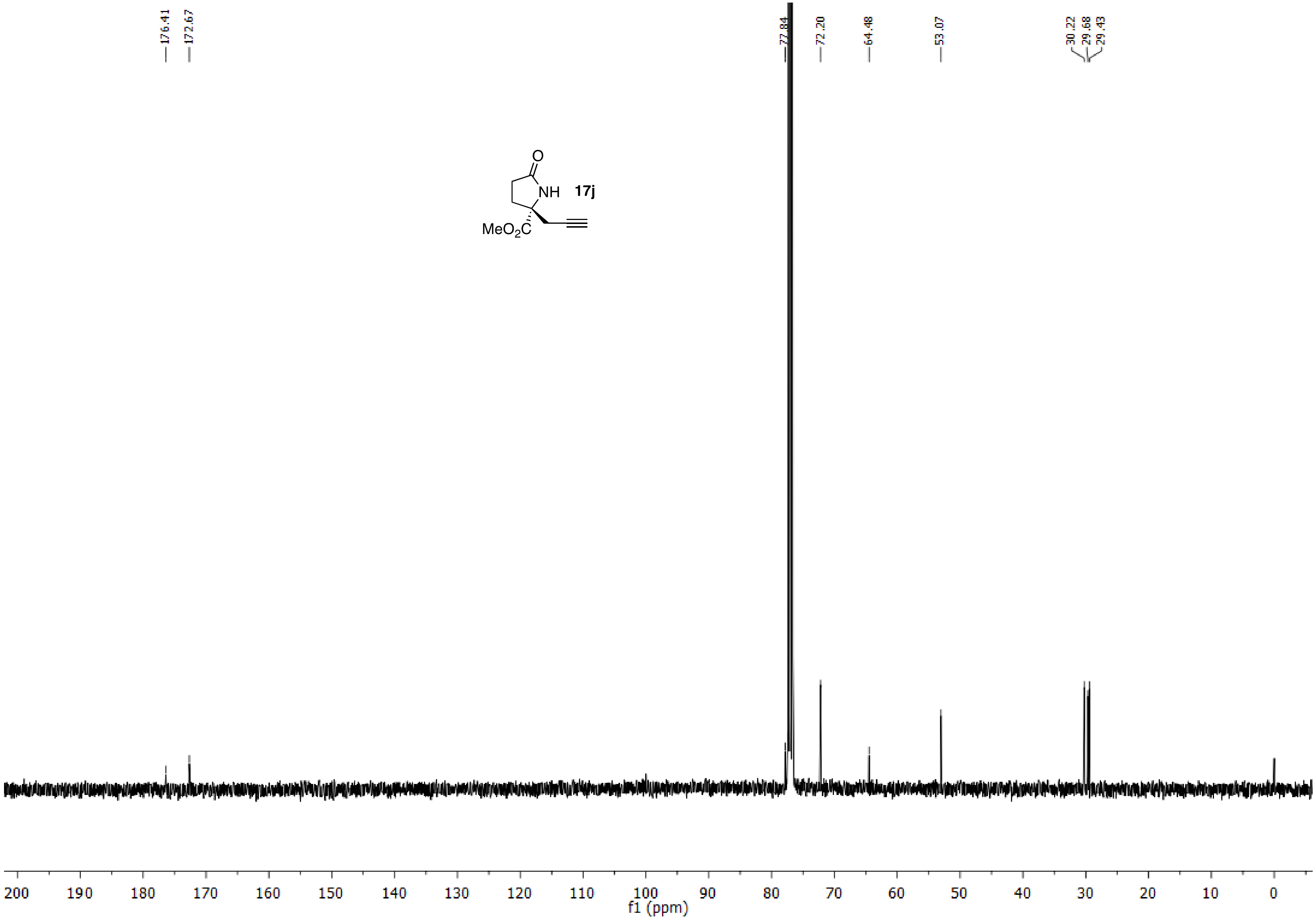
17j
176.41
172.67
77.84
72.20
64.48
53.07
30.22
29.68
29.43
200 190 180 170 160 150 140 130 120 110 100 90 80 70 60 50 40 30 20 10 0
f1 (ppm)

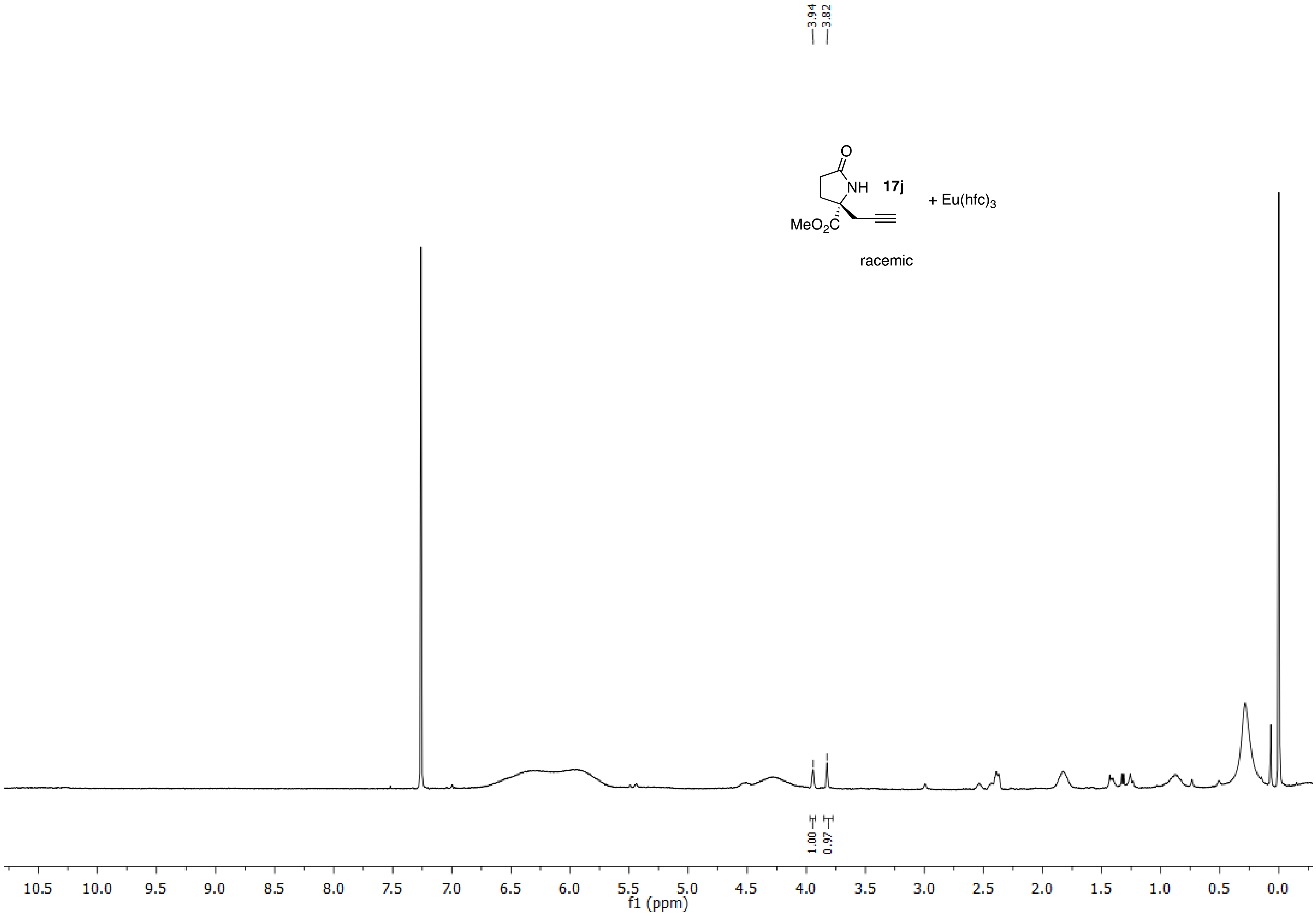
3.94
3.82
O
NH
17j
MeO$_2$C
+ Eu(hfc)$_3$
racemic
1.00
0.97
10.5 10.0 9.5 9.0 8.5 8.0 7.5 7.0 6.5 6.0 5.5 5.0 4.5 4.0 3.5 3.0 2.5 2.0 1.5 1.0 0.5 0.0
f1 (ppm)

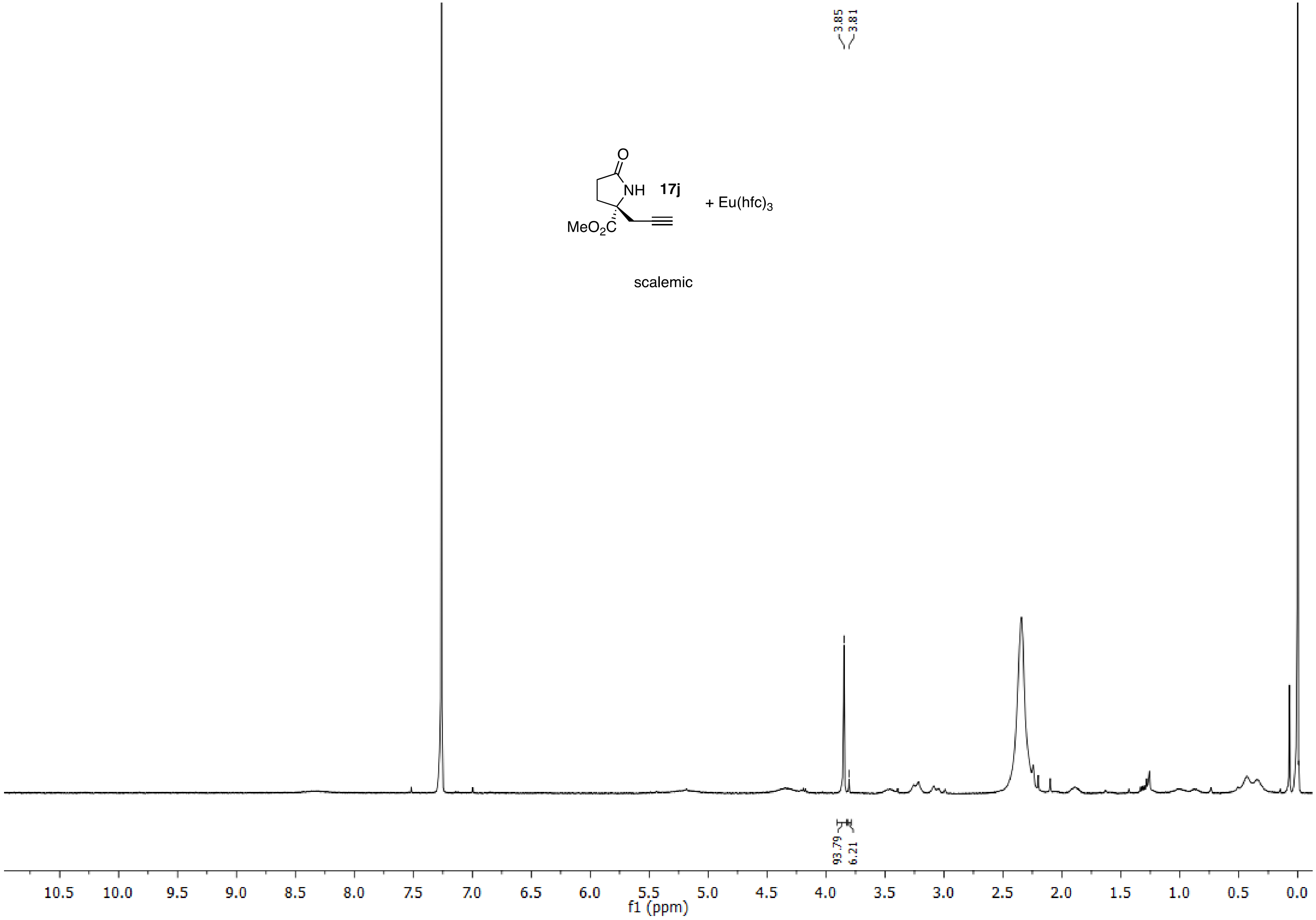
3.85
3.81
O
NH
MeO2C
17j
+ Eu(hfc)3
scalemic
93.79
6.21
10.5 10.0 9.5 9.0 8.5 8.0 7.5 7.0 6.5 6.0 5.5 5.0 4.5 4.0 3.5 3.0 2.5 2.0 1.5 1.0 0.5 0.0
f1 (ppm)

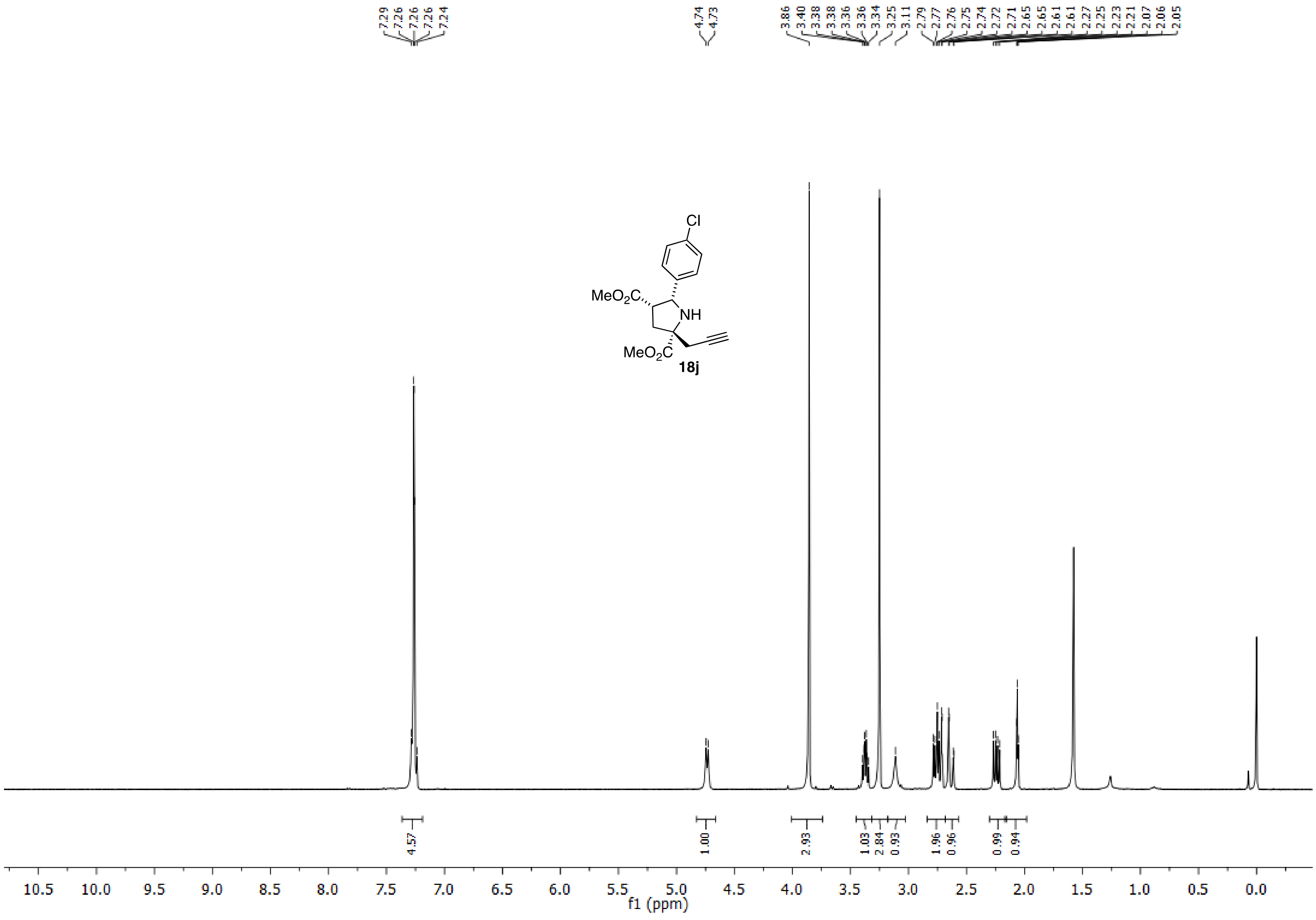
7.29
7.26
7.26
7.26
7.24
4.74
4.73
3.86
3.40
3.38
3.38
3.36
3.36
3.34
3.25
3.11
2.79
2.77
2.76
2.75
2.74
2.72
2.71
2.65
2.65
2.61
2.61
2.27
2.25
2.23
2.21
2.07
2.06
2.05
Cl
MeO2C
NH
MeO2C
18j
4.57
1.00
2.93
1.03
2.84
0.93
1.96
0.96
0.99
0.94
10.5
10.0
9.5
9.0
8.5
8.0
7.5
7.0
6.5
6.0
5.5
5.0
4.5
4.0
3.5
3.0
2.5
2.0
1.5
1.0
0.5
0.0
f1 (ppm)

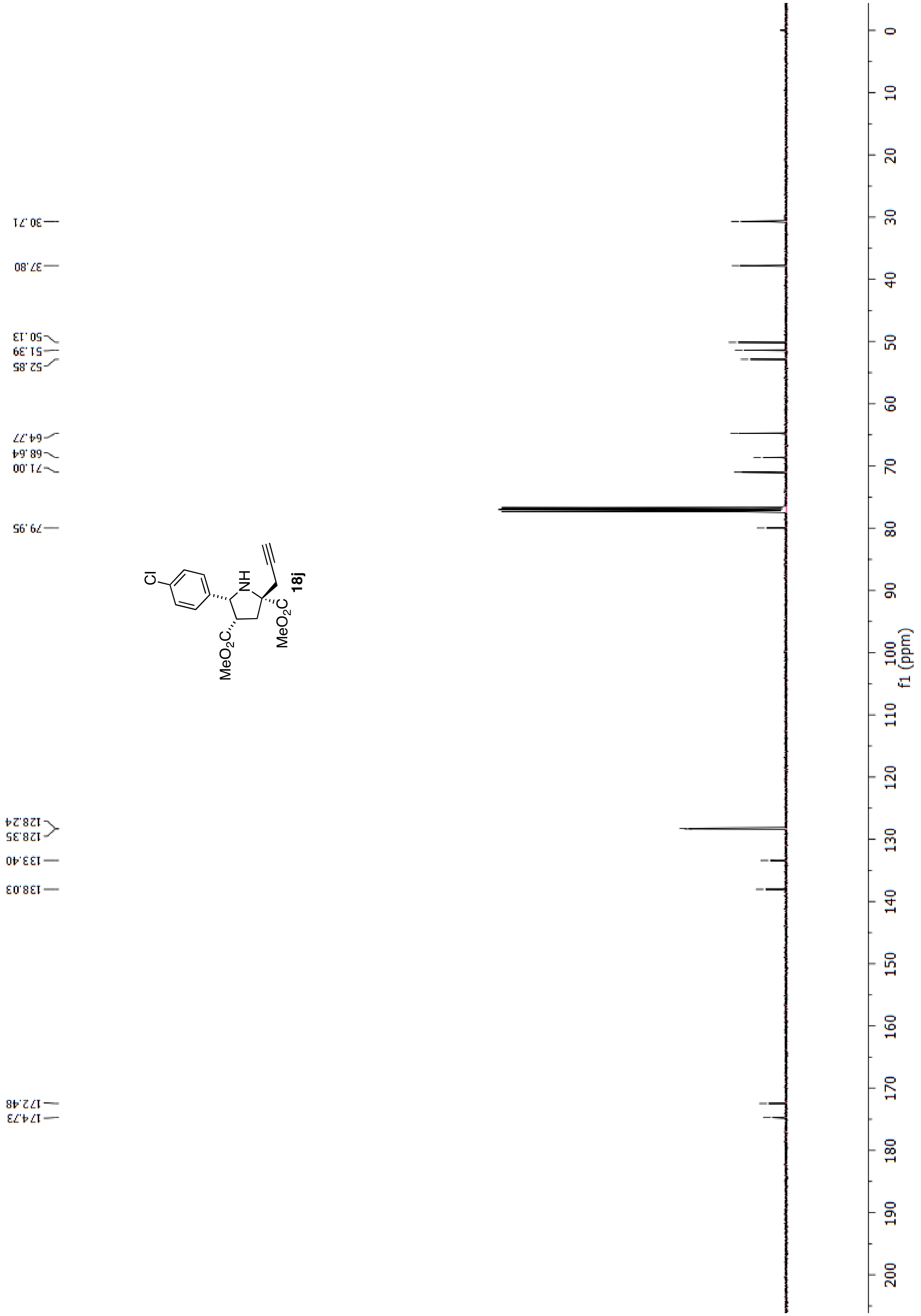
30.71
37.80
50.13
51.39
52.85
64.77
68.64
71.00
79.95
128.24
128.35
133.40
138.03
172.48
174.73
Cl
NH
MeO2C
MeO2C
18j
f1 (ppm)

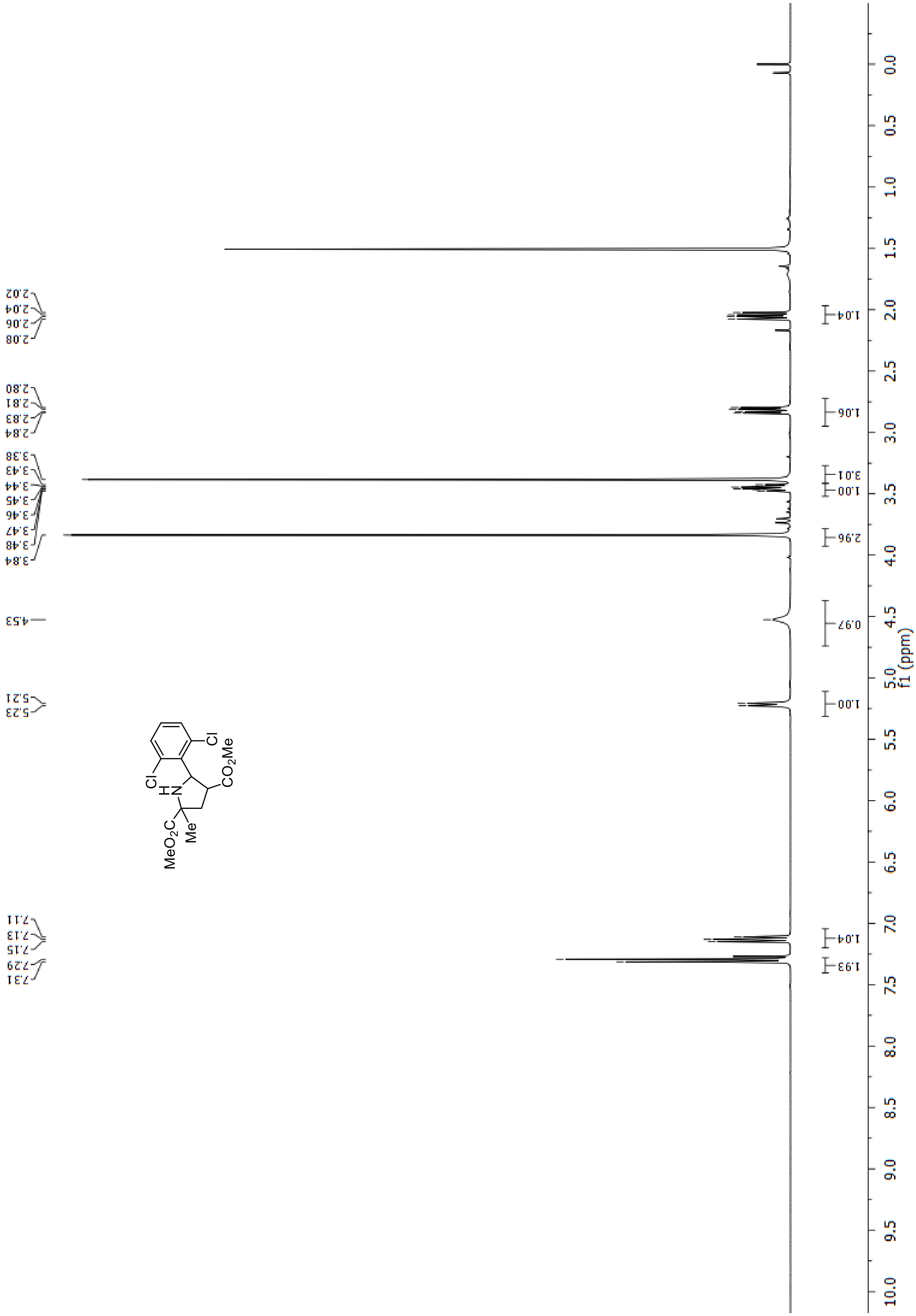
2.02
2.04
2.06
2.08
2.80
2.81
2.83
2.84
3.38
3.43
3.44
3.45
3.46
3.47
3.48
3.84
4.53
5.21
5.23
7.11
7.13
7.15
7.29
7.31
Cl
Cl
HN
MeO2C
Me
CO2Me
1.04
1.06
3.01
1.00
2.96
0.97
1.00
1.04
1.93
0.0
0.5
1.0
1.5
2.0
2.5
3.0
3.5
4.0
4.5
5.0
5.5
6.0
6.5
7.0
7.5
8.0
8.5
9.0
9.5
10.0
f1 (ppm)

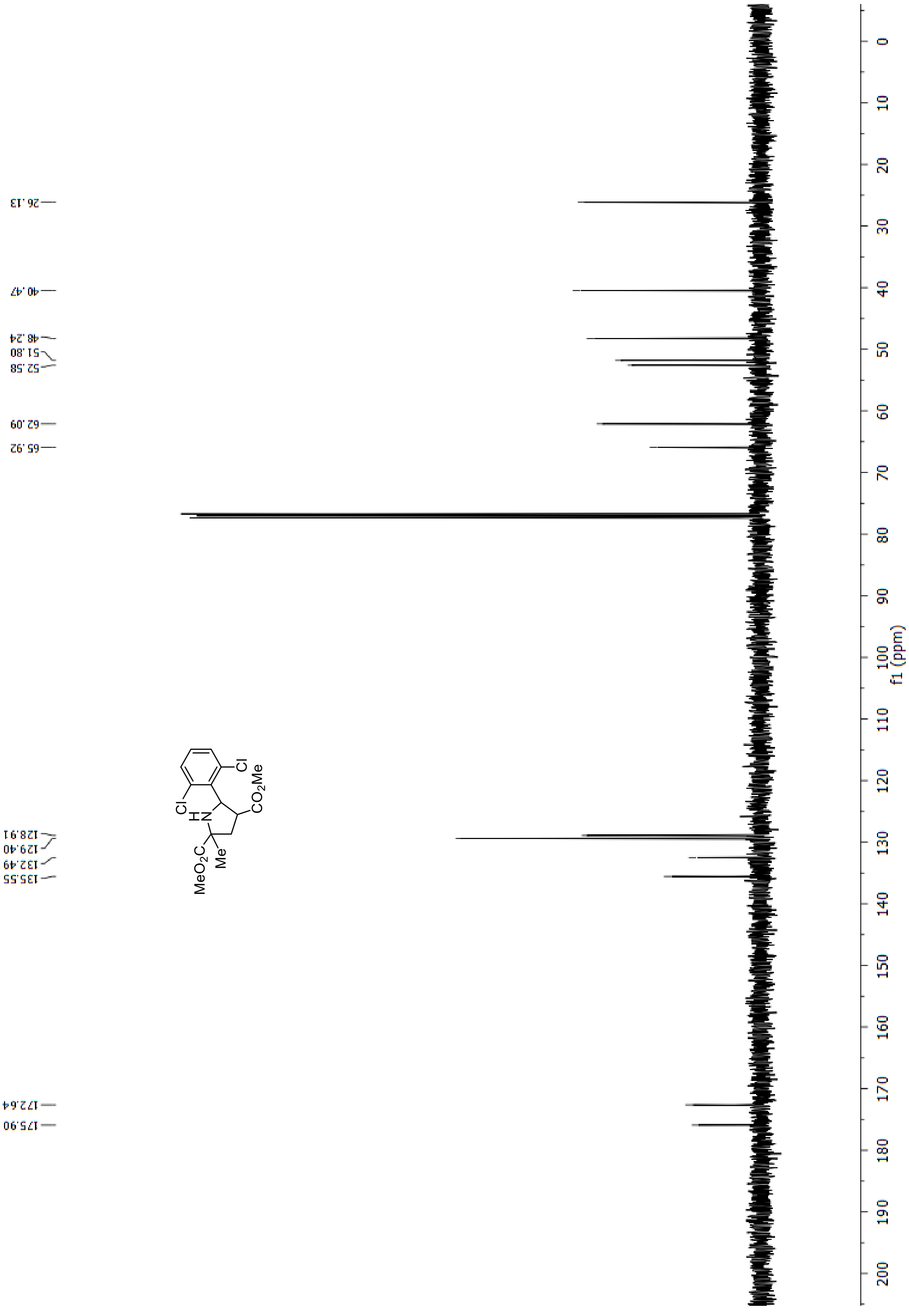
26.13
40.47
48.24
51.80
52.58
62.09
65.92
128.91
129.40
132.49
135.55
172.64
175.90
f1 (ppm)
Cl
Cl
CO2Me
MeO2C
Me
HN

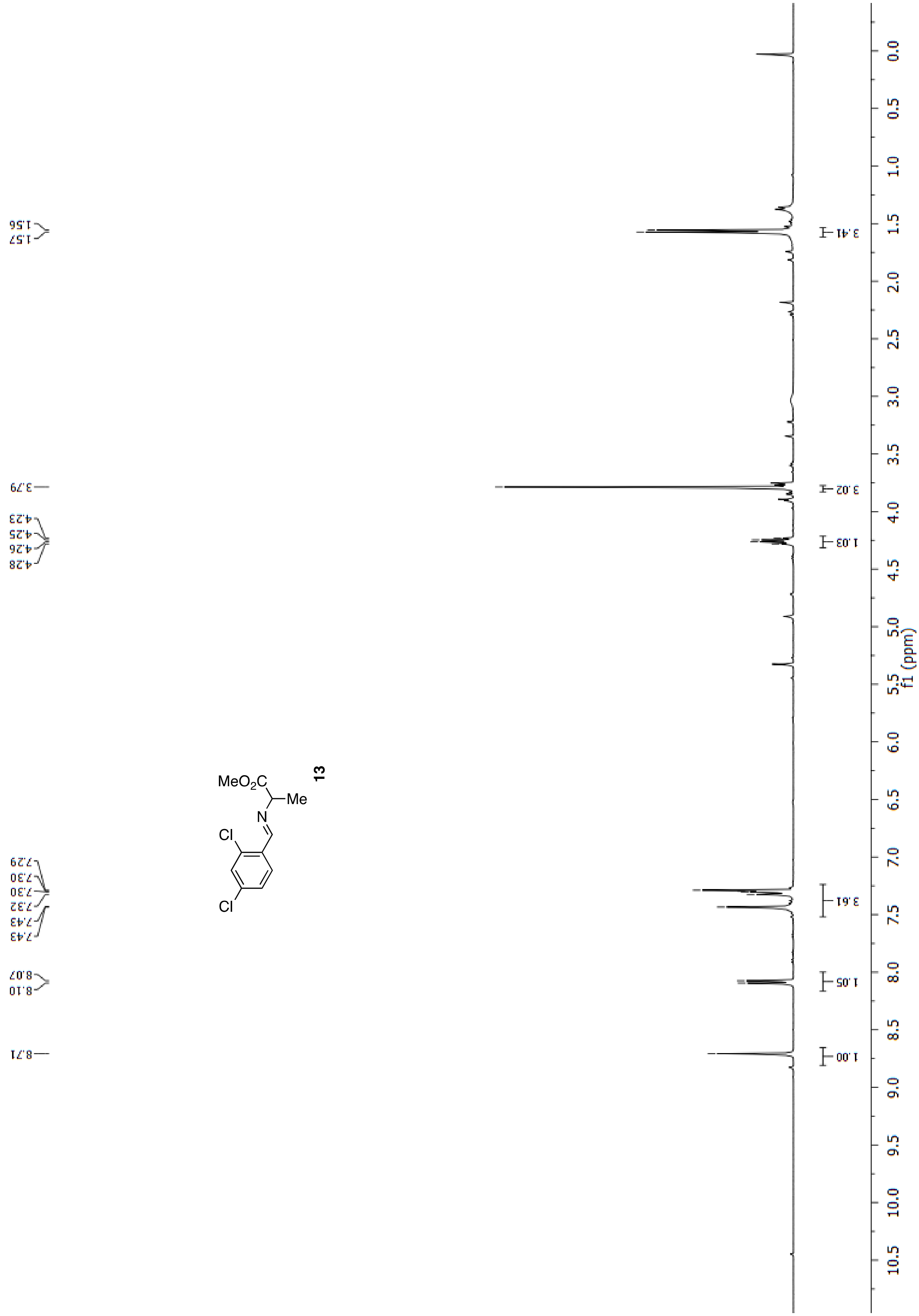
8.71
8.10
8.07
7.43
7.43
7.32
7.30
7.30
7.29
4.28
4.26
4.25
4.23
3.79
1.57
1.56
MeO2C
N
Me
Cl
Cl
13
1.00
1.05
3.61
1.03
3.02
3.41
10.5
10.0
9.5
9.0
8.5
8.0
7.5
7.0
6.5
6.0
5.5
5.0
4.5
4.0
3.5
3.0
2.5
2.0
1.5
1.0
0.5
0.0
f1 (ppm)

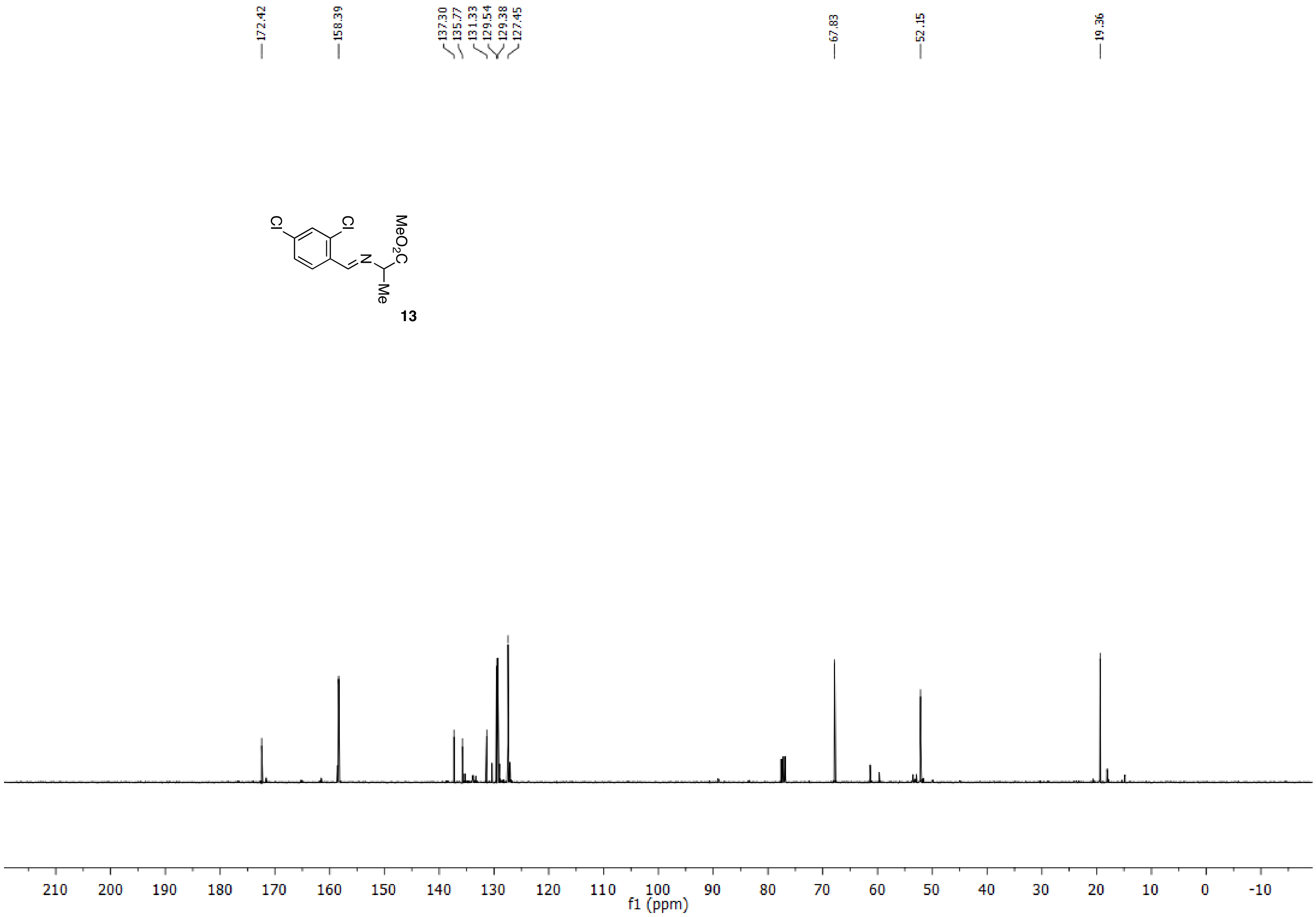
172.42
158.39
137.30
135.77
131.33
129.54
129.38
127.45
67.83
52.15
19.36
Cl
Cl
MeO2C
N
Me
13
210 200 190 180 170 160 150 140 130 120 110 100 90 80 70 60 50 40 30 20 10 0 -10
f1 (ppm)

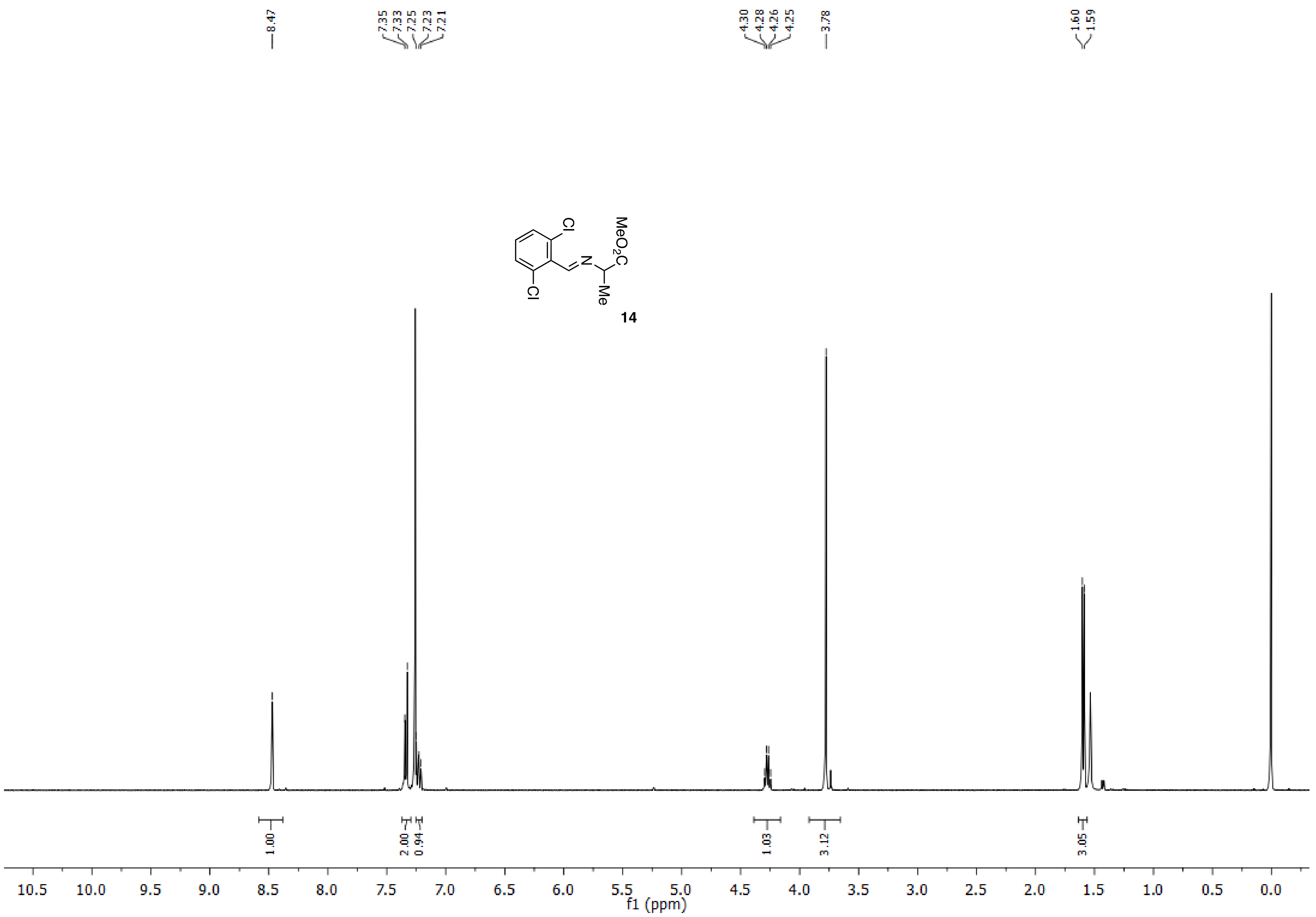
8.47
7.35
7.33
7.25
7.23
7.21
4.30
4.28
4.26
4.25
3.78
1.60
1.59
MeO2C
Me
N
Cl
Cl
14
1.00
2.00
0.94
1.03
3.12
3.05
10.5
10.0
9.5
9.0
8.5
8.0
7.5
7.0
6.5
6.0
5.5
5.0
4.5
4.0
3.5
3.0
2.5
2.0
1.5
1.0
0.5
0.0
f1 (ppm)

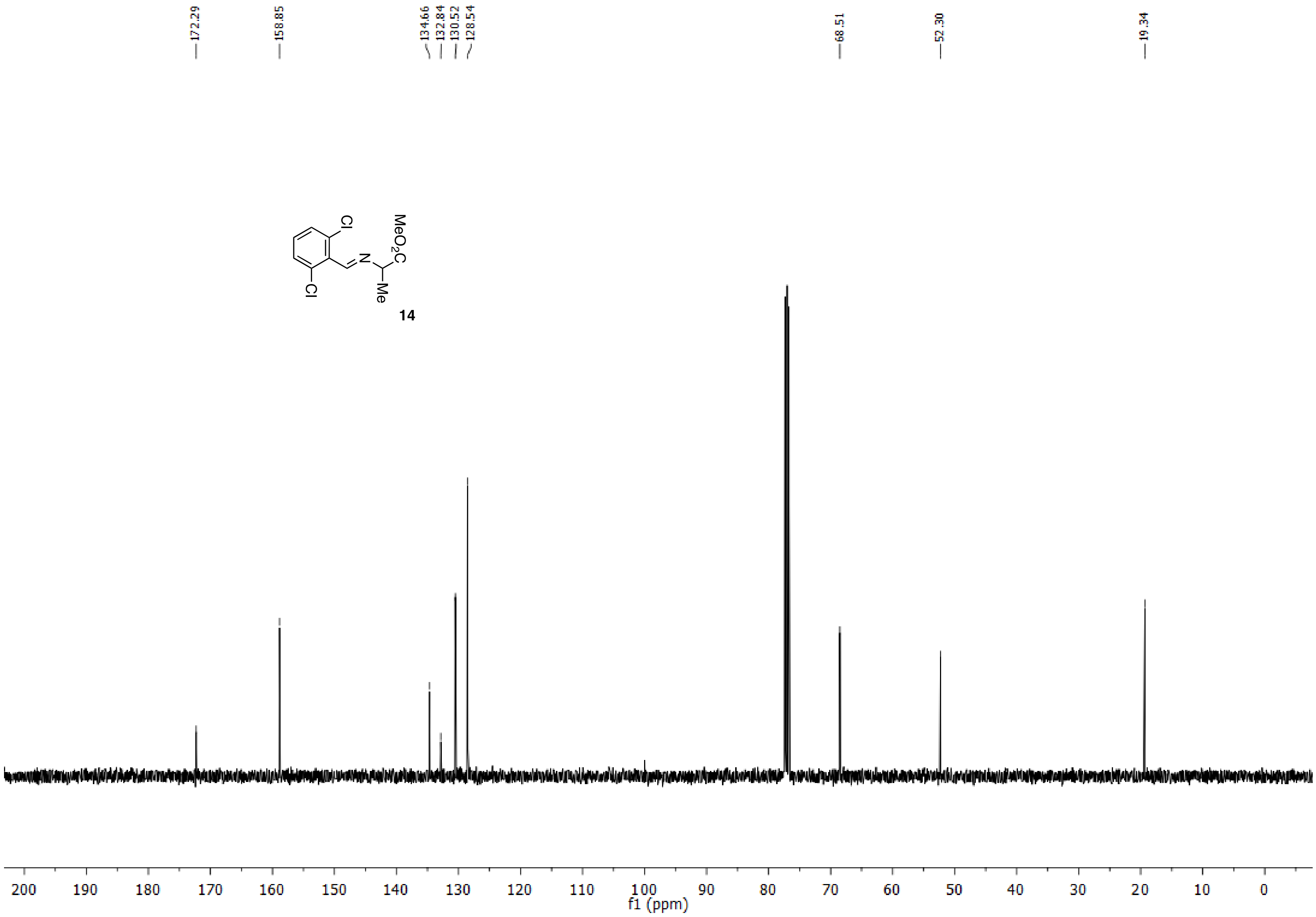
172.29
158.85
134.66
132.84
130.52
128.54
68.51
52.30
19.34
MeO2C
N
Me
Cl
Cl
14
200 190 180 170 160 150 140 130 120 110 100 90 80 70 60 50 40 30 20 10 0
f1 (ppm)

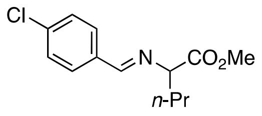
Cl
N
CO2Me
n-Pr

16c

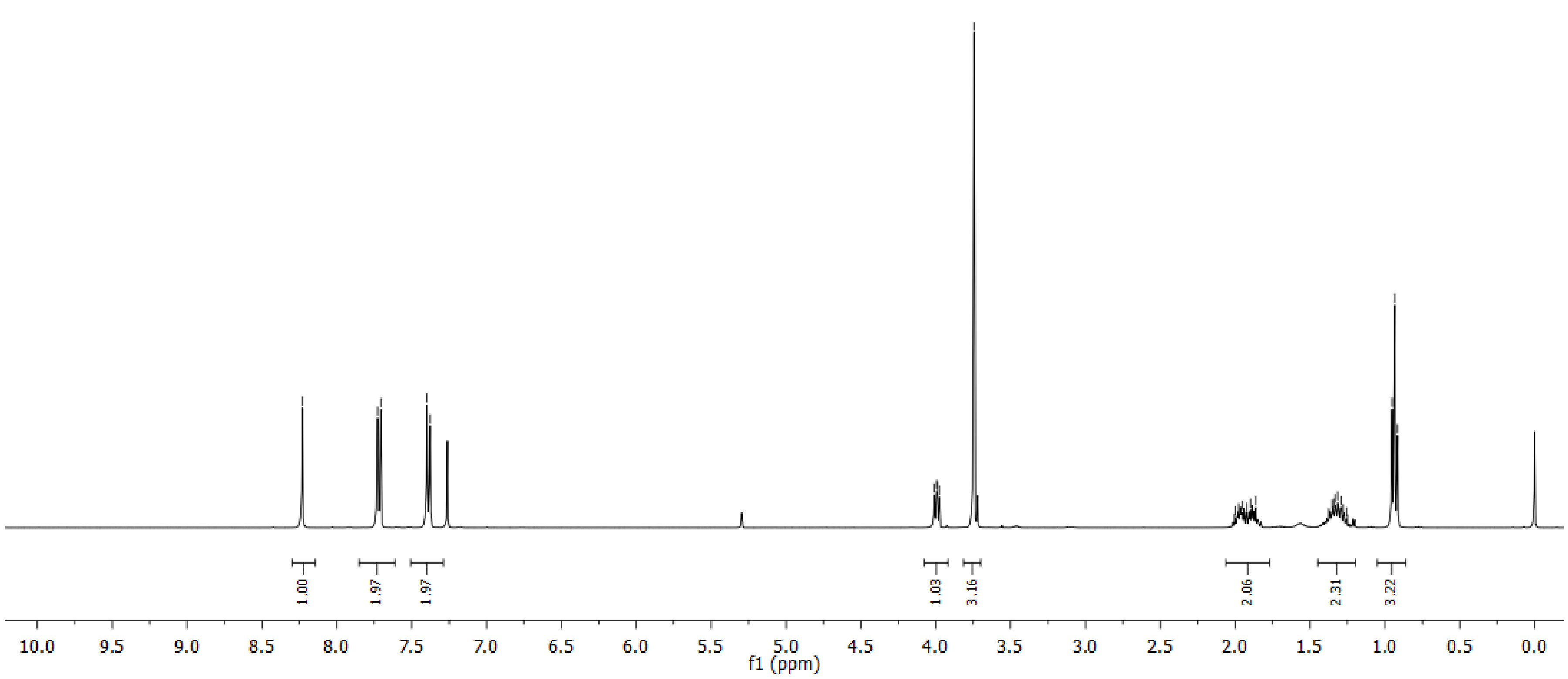
8.23
7.73
7.71
7.40
7.38
4.01
4.00
3.99
3.97
3.74
2.01
2.00
1.98
1.95
1.92
1.90
1.86
1.37
1.35
1.34
1.33
1.31
1.29
1.28
1.26
1.25
0.95
0.93
0.92
1.00
1.97
1.97
1.03
3.16
2.06
2.31
3.22
10.0
9.5
9.0
8.5
8.0
7.5
7.0
6.5
6.0
5.5
5.0
4.5
4.0
3.5
3.0
2.5
2.0
1.5
1.0
0.5
0.0
f1 (ppm)

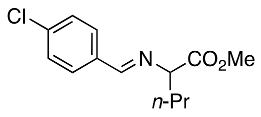
Cl
N
CO2Me
n-Pr

16c

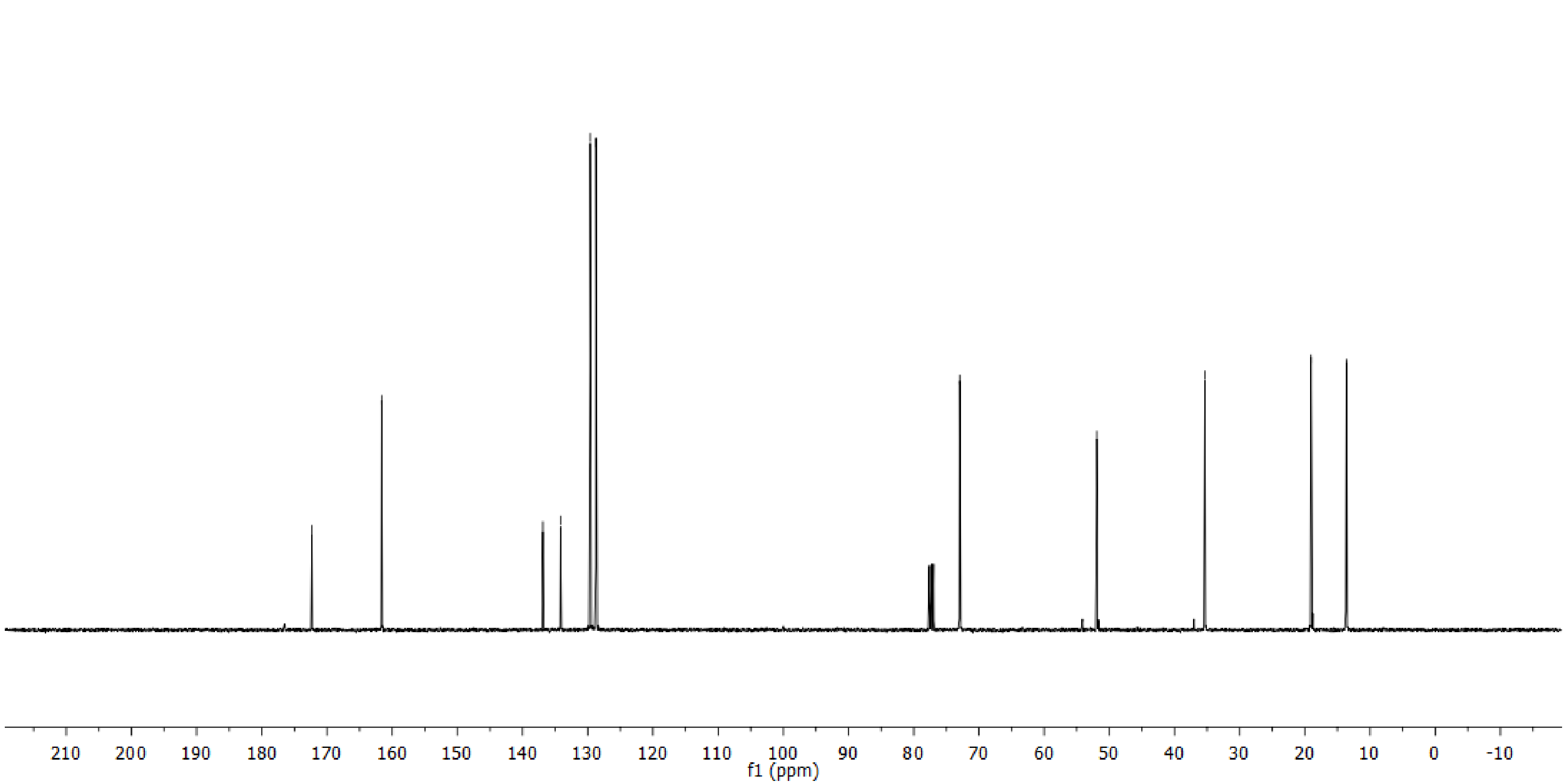
172.31
161.61
136.88
134.17
129.63
128.72
72.93
51.90
35.34
19.04
13.60
210 200 190 180 170 160 150 140 130 120 110 100 90 80 70 60 50 40 30 20 10 0 -10
f1 (ppm)

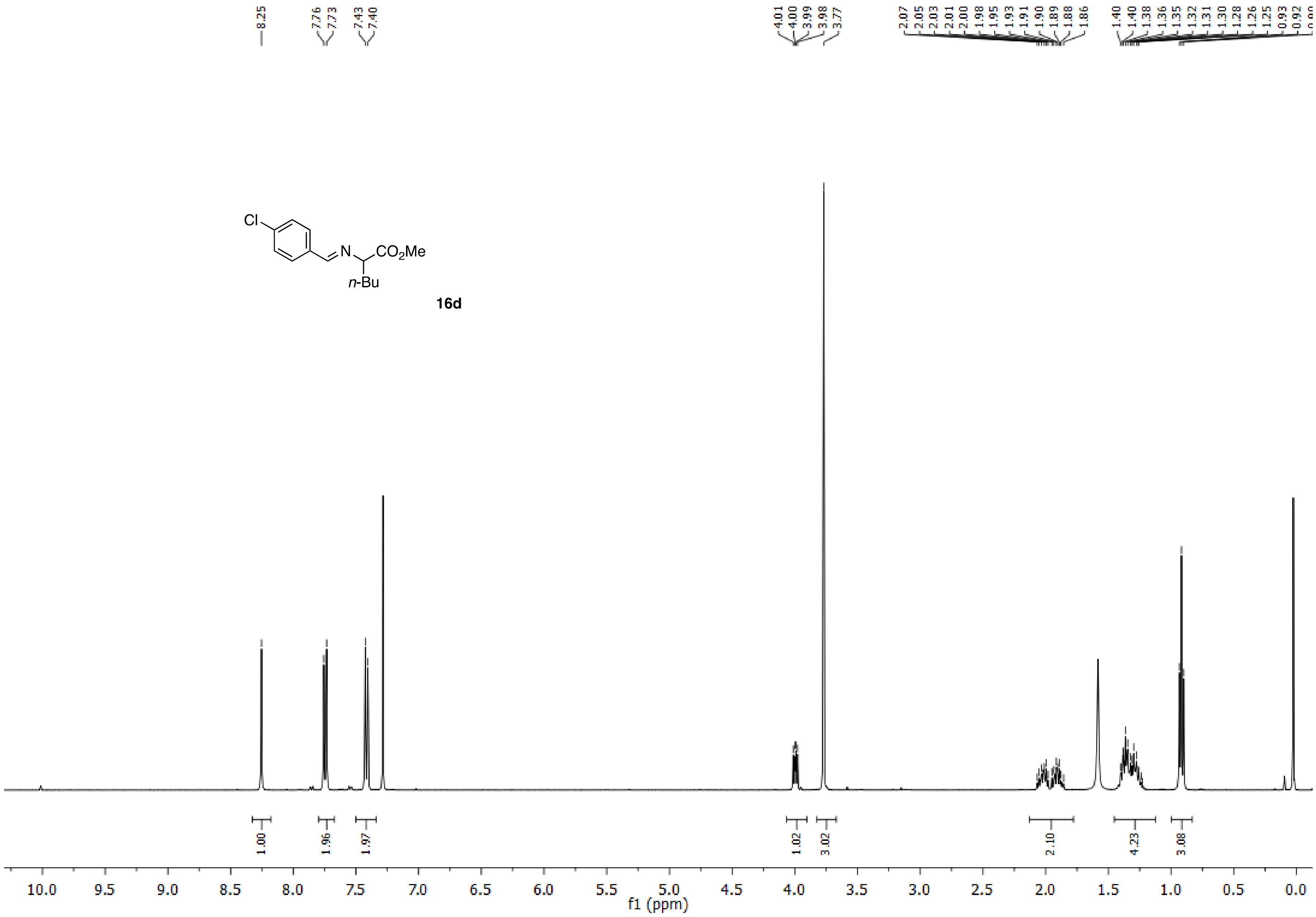
8.25
7.76
7.73
7.43
7.40
4.01
4.00
3.99
3.98
3.77
2.07
2.05
2.03
2.01
2.00
1.98
1.95
1.93
1.91
1.90
1.89
1.88
1.86
1.40
1.40
1.38
1.36
1.35
1.32
1.31
1.30
1.28
1.26
1.25
0.93
0.92
Cl
N
CO_2Me
n-Bu
1.00
1.96
1.97
1.02
3.02
2.10
4.23
3.08
f1 (ppm)

16d

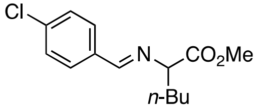
Cl
N
CO_2Me
n-Bu

16d

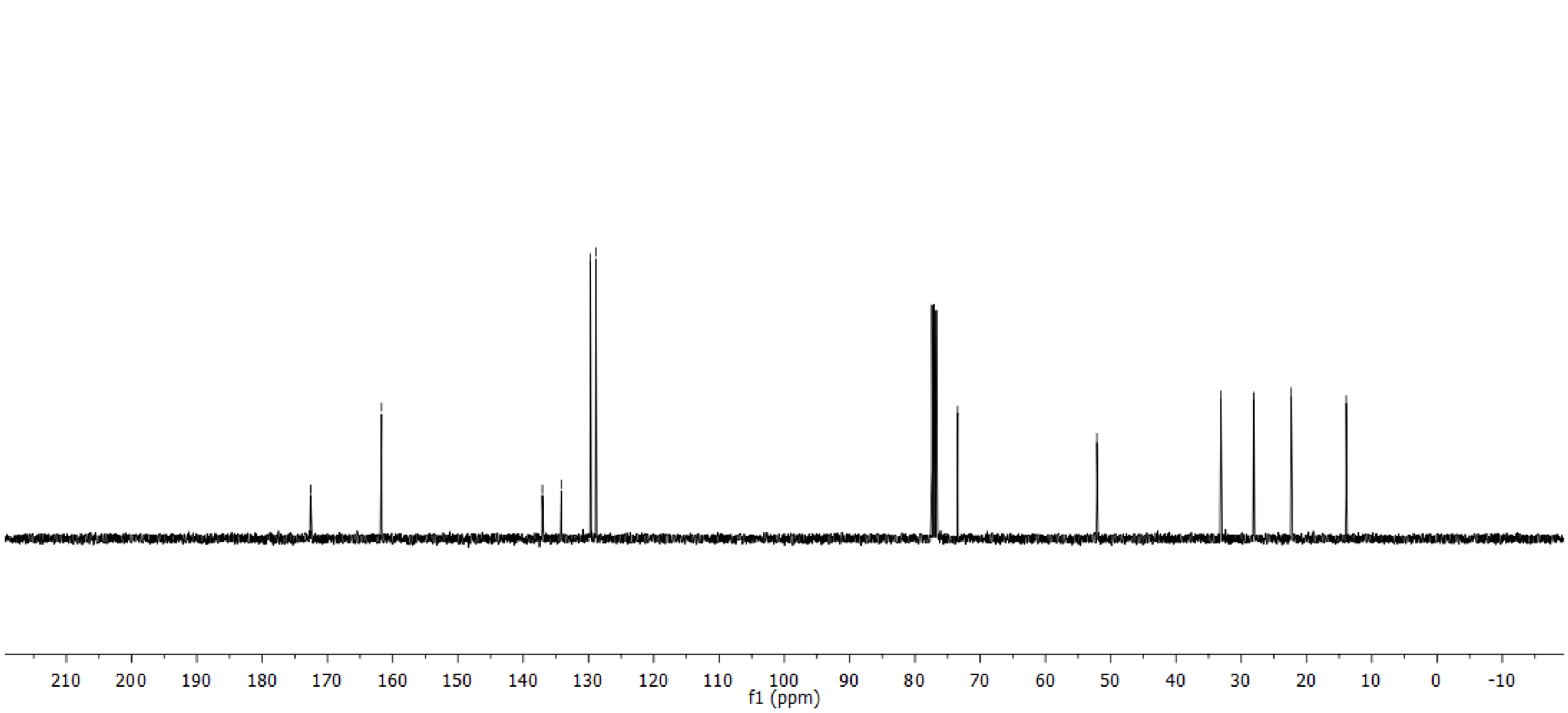
172.58
161.72
137.09
134.17
129.72
128.86
73.46
52.12
33.12
28.07
22.33
13.91
210 200 190 180 170 160 150 140 130 120 110 100 90 80 70 60 50 40 30 20 10 0 -10
f1 (ppm)

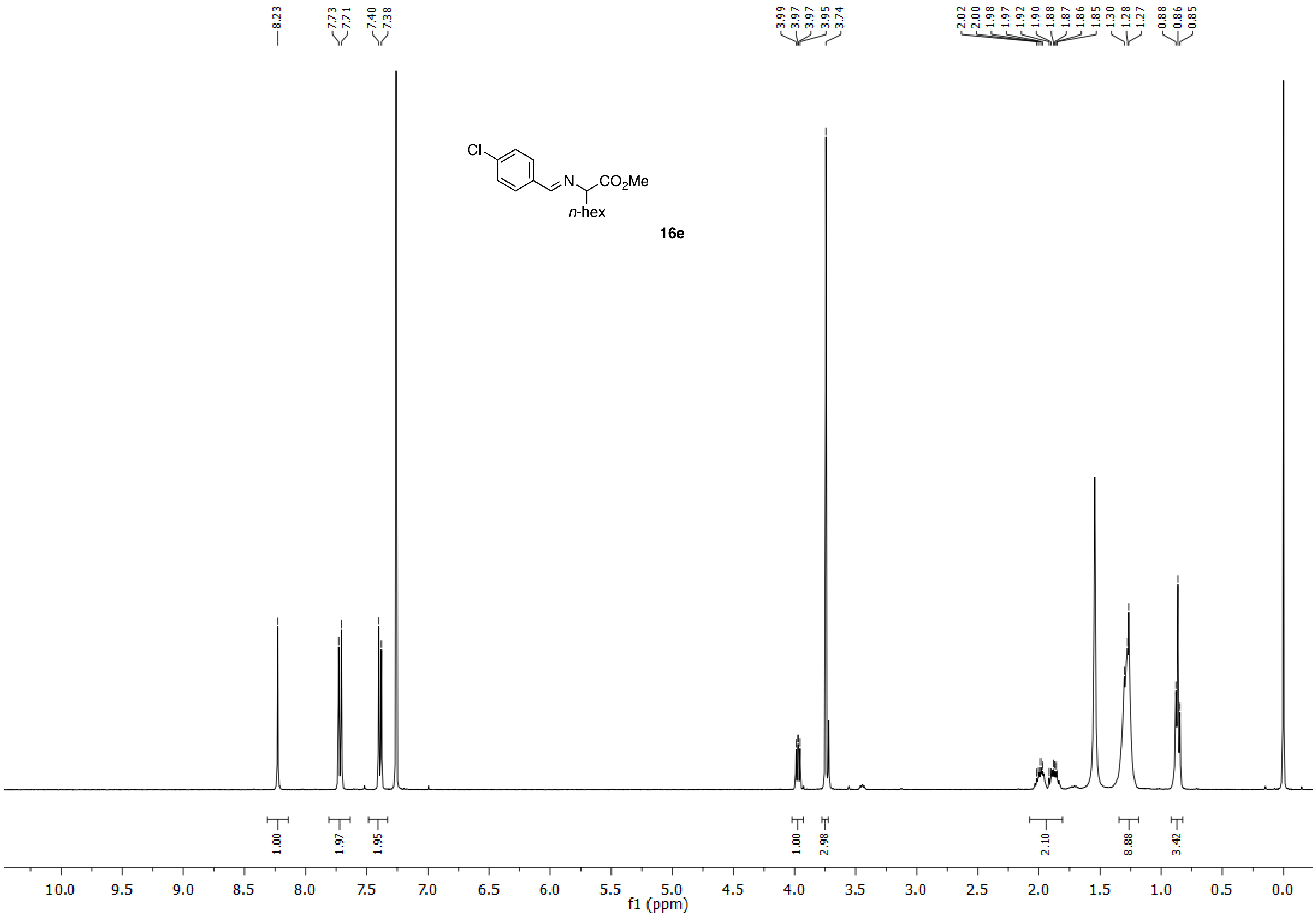
8.23
7.73
7.71
7.40
7.38
3.99
3.97
3.97
3.95
3.74
2.02
2.00
1.98
1.97
1.92
1.90
1.88
1.87
1.86
1.85
1.30
1.28
1.27
0.88
0.86
0.85
Cl
N
CO2Me
n-hex
16e
1.00
1.97
1.95
1.00
2.98
2.10
8.88
3.42
10.0 9.5 9.0 8.5 8.0 7.5 7.0 6.5 6.0 5.5 5.0 4.5 4.0 3.5 3.0 2.5 2.0 1.5 1.0 0.5 0.0
f1 (ppm)

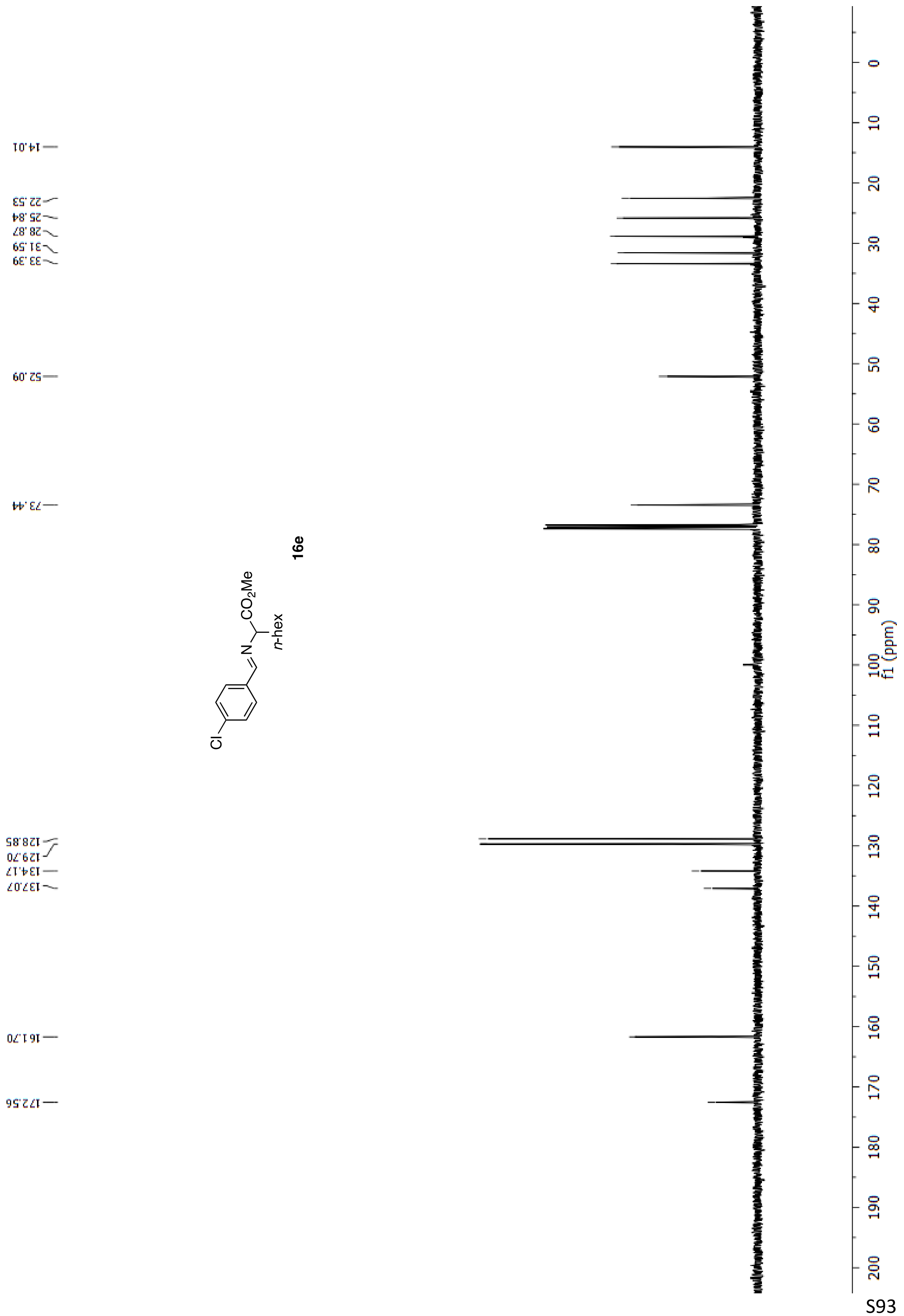

14.01
22.53
25.84
28.87
31.59
33.39
52.09
73.44
128.85
129.70
134.17
137.07
161.70
172.56
Cl
N
CO2Me
n-hex
16e
f1 (ppm)

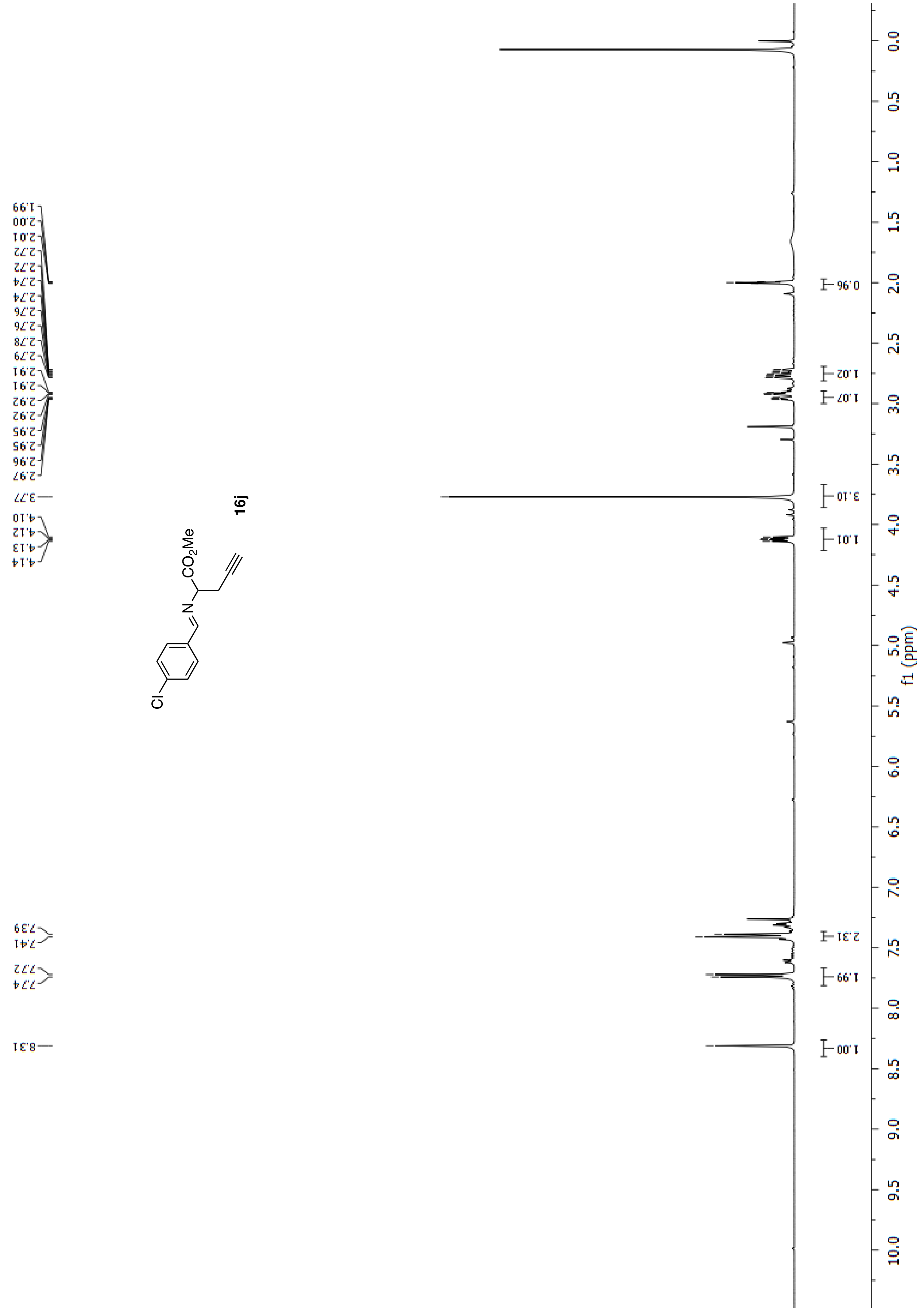
16j
f1 (ppm)

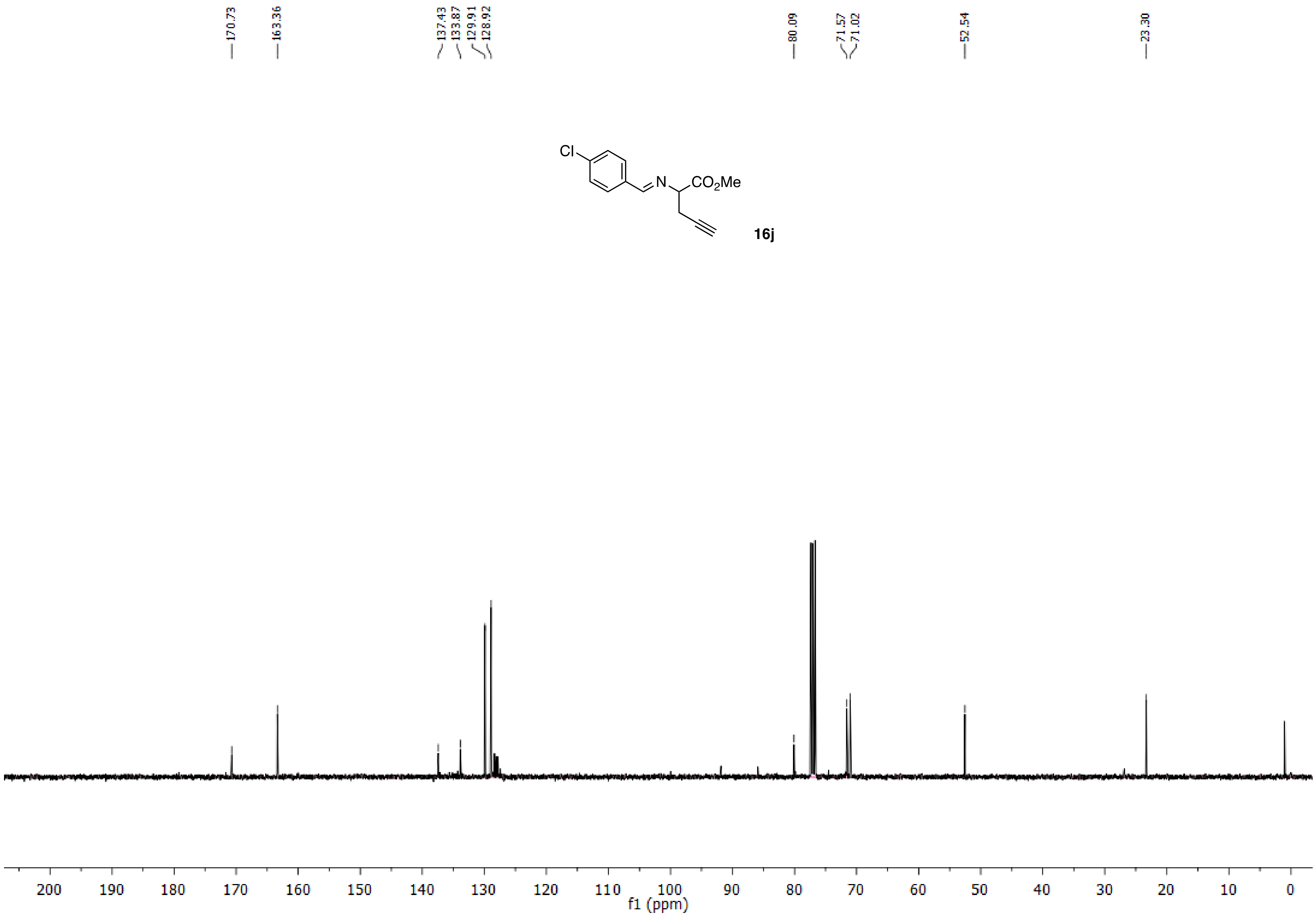
170.73
163.36
137.43
133.87
129.91
128.92
80.09
71.57
71.02
52.54
23.30
Cl
N
CO_2Me
16j
200
190
180
170
160
150
140
130
120
110
100
90
80
70
60
50
40
30
20
10
0
f1 (ppm)

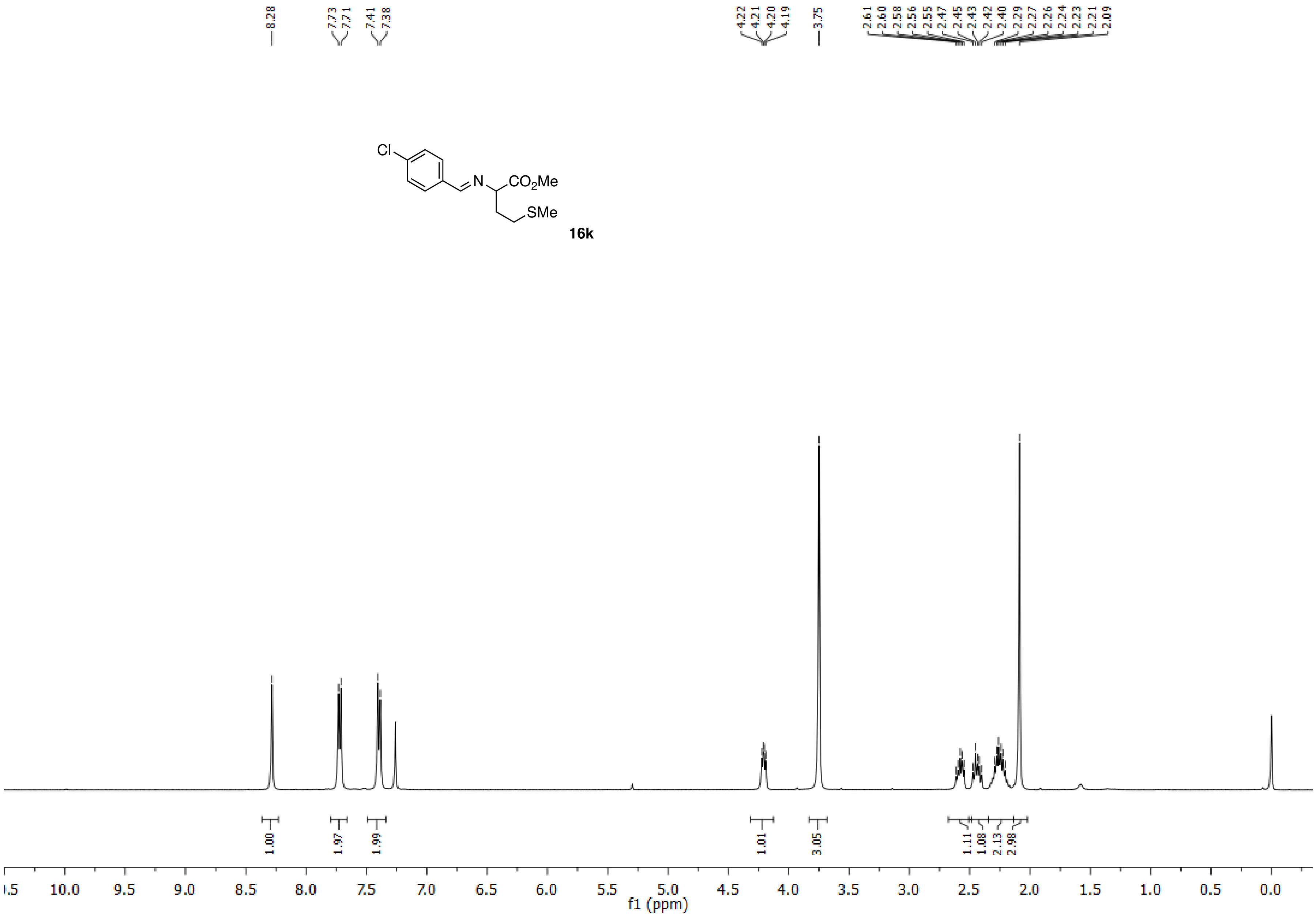

8.28
7.73
7.71
7.41
7.38
4.22
4.21
4.20
4.19
3.75
2.61
2.60
2.58
2.56
2.55
2.47
2.45
2.43
2.42
2.40
2.29
2.27
2.26
2.24
2.23
2.21
2.09
Cl
N
CO_2Me
SMe
1.00
1.97
1.99
1.01
3.05
1.11
1.08
2.13
2.98
f1 (ppm)

16k

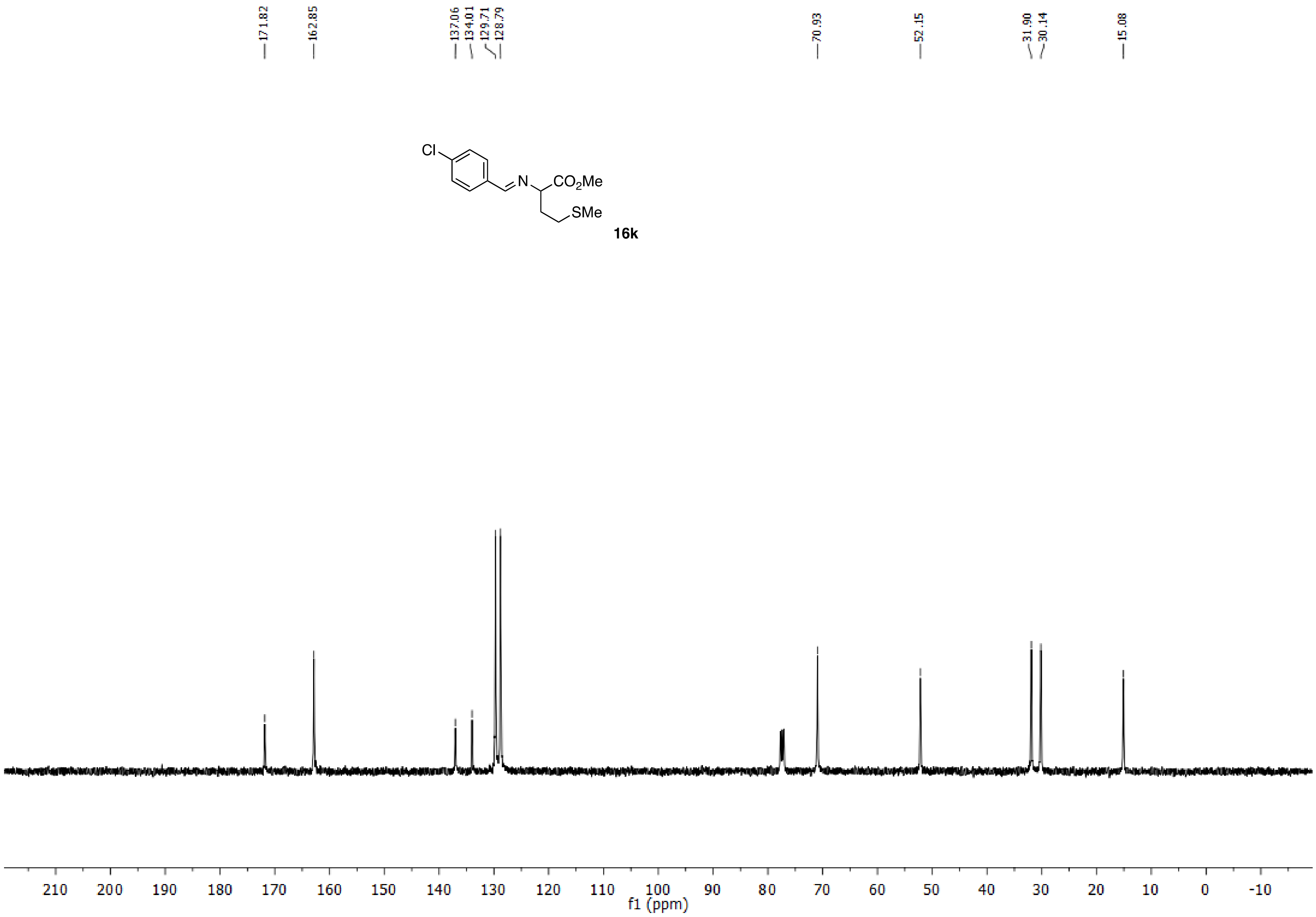
171.82
162.85
137.06
134.01
129.71
128.79
70.93
52.15
31.90
30.14
15.08
Cl
N
CO_2Me
SMe
16k
210 200 190 180 170 160 150 140 130 120 110 100 90 80 70 60 50 40 30 20 10 0 -10
f1 (ppm)

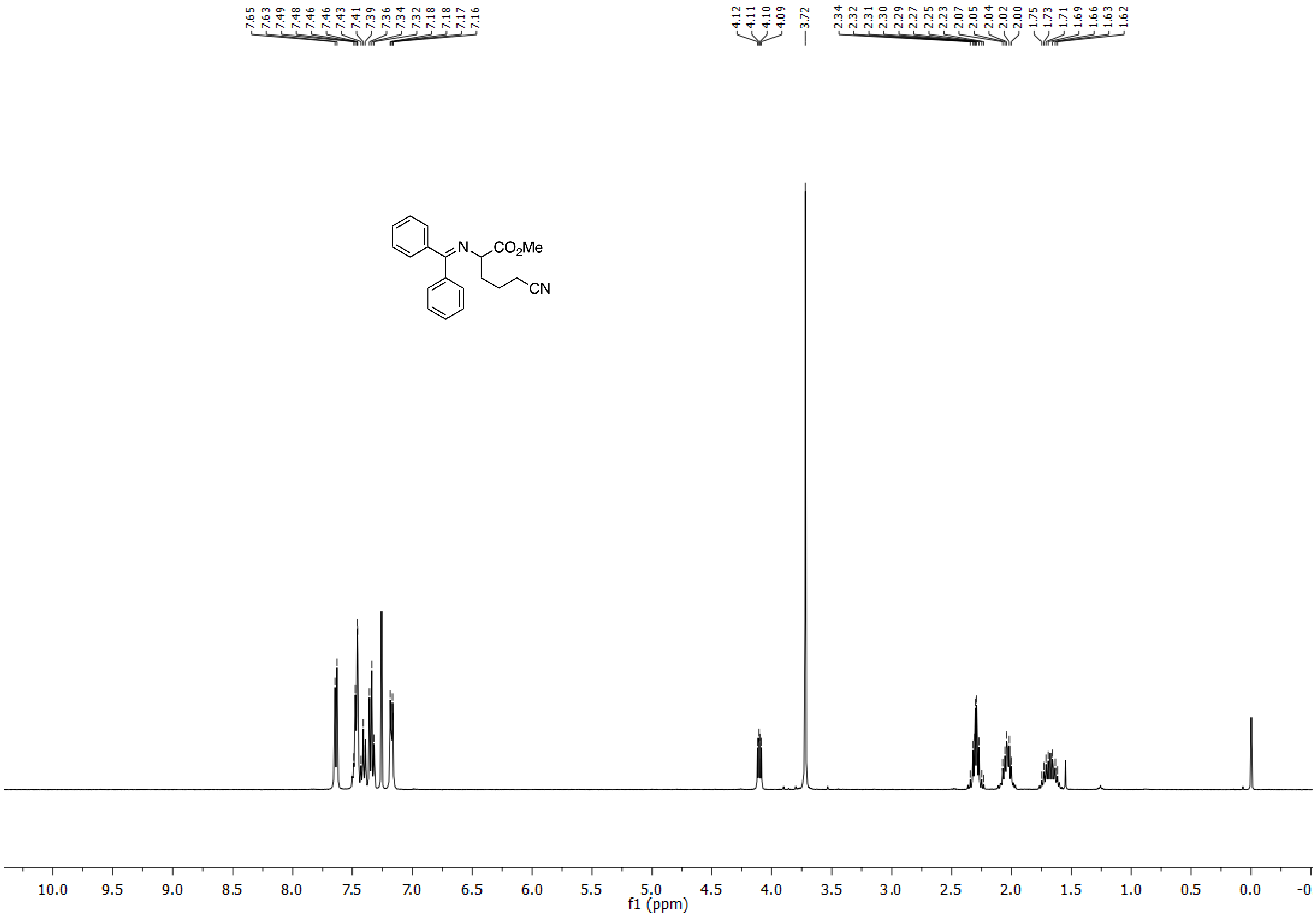
7.65
7.63
7.49
7.48
7.46
7.46
7.43
7.41
7.39
7.36
7.34
7.32
7.18
7.18
7.17
7.16
4.12
4.11
4.10
4.09
3.72
2.34
2.32
2.31
2.30
2.29
2.27
2.25
2.23
2.07
2.05
2.04
2.02
2.00
1.75
1.73
1.71
1.69
1.66
1.63
1.62
N
CO2Me
CN
10.0
9.5
9.0
8.5
8.0
7.5
7.0
6.5
6.0
5.5
5.0
4.5
4.0
3.5
3.0
2.5
2.0
1.5
1.0
0.5
0.0
-0
f1 (ppm)

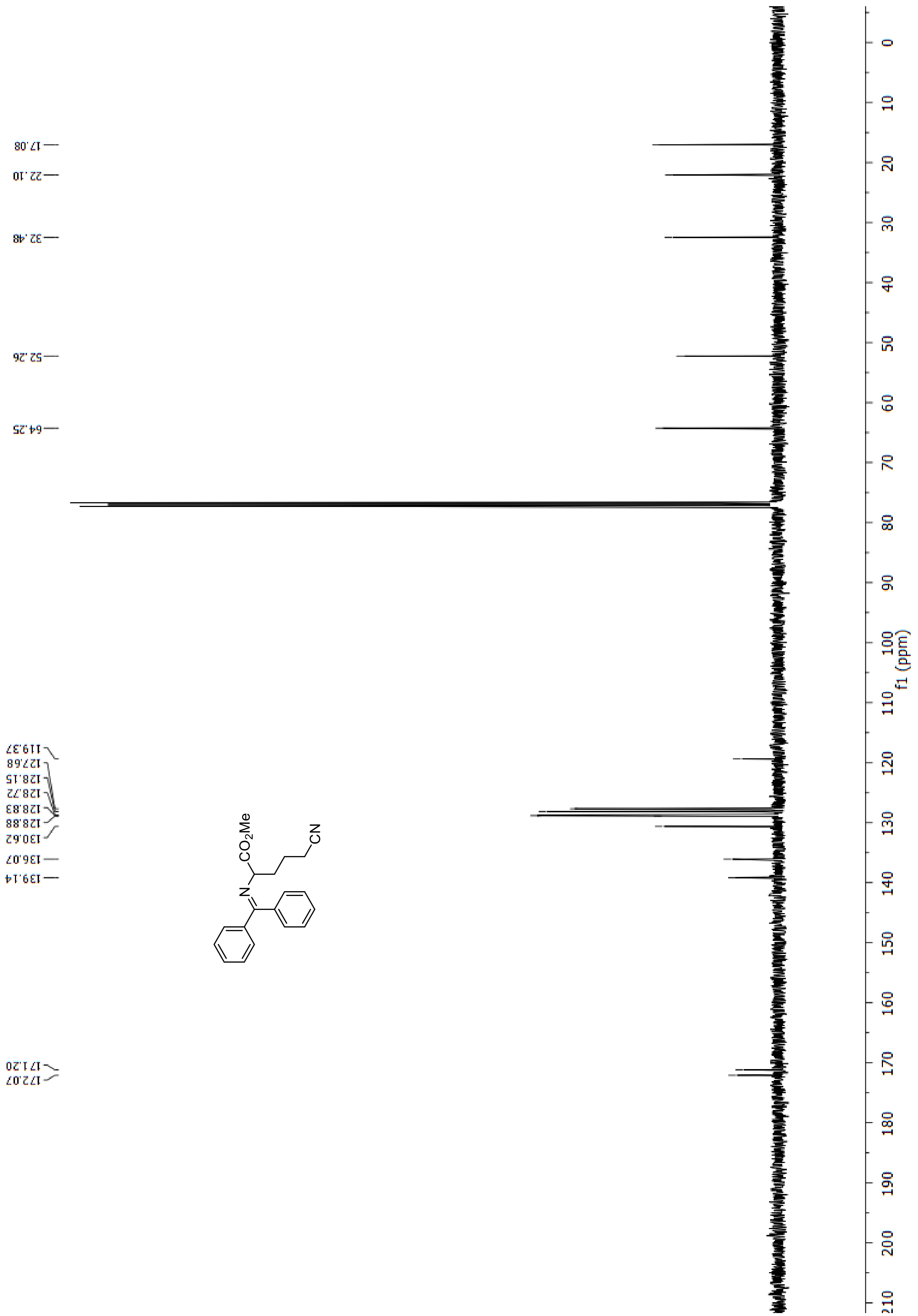
17.08
22.10
32.48
52.26
64.25
119.37
127.68
128.15
128.72
128.83
128.88
130.62
136.07
139.14
171.20
172.07
N
CO2Me
CN
f1 (ppm)

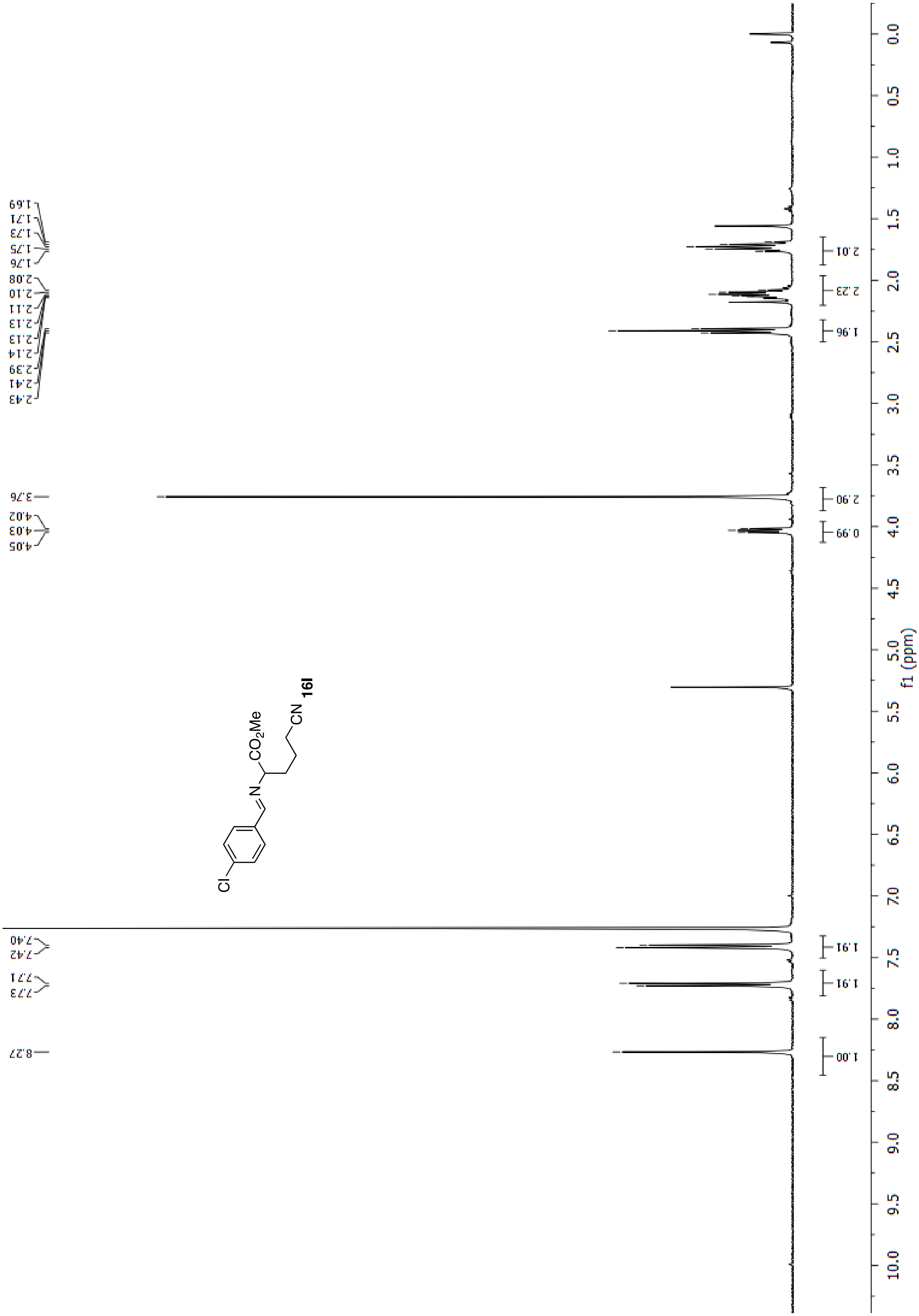
1.69
1.71
1.73
1.75
1.76
2.08
2.10
2.11
2.13
2.13
2.14
2.39
2.41
2.43
3.76
4.02
4.03
4.05
7.40
7.42
7.71
7.73
8.27
Cl
N
CO2Me
CN
16l
2.01
2.23
1.96
2.90
0.99
1.91
1.91
1.00
f1 (ppm)

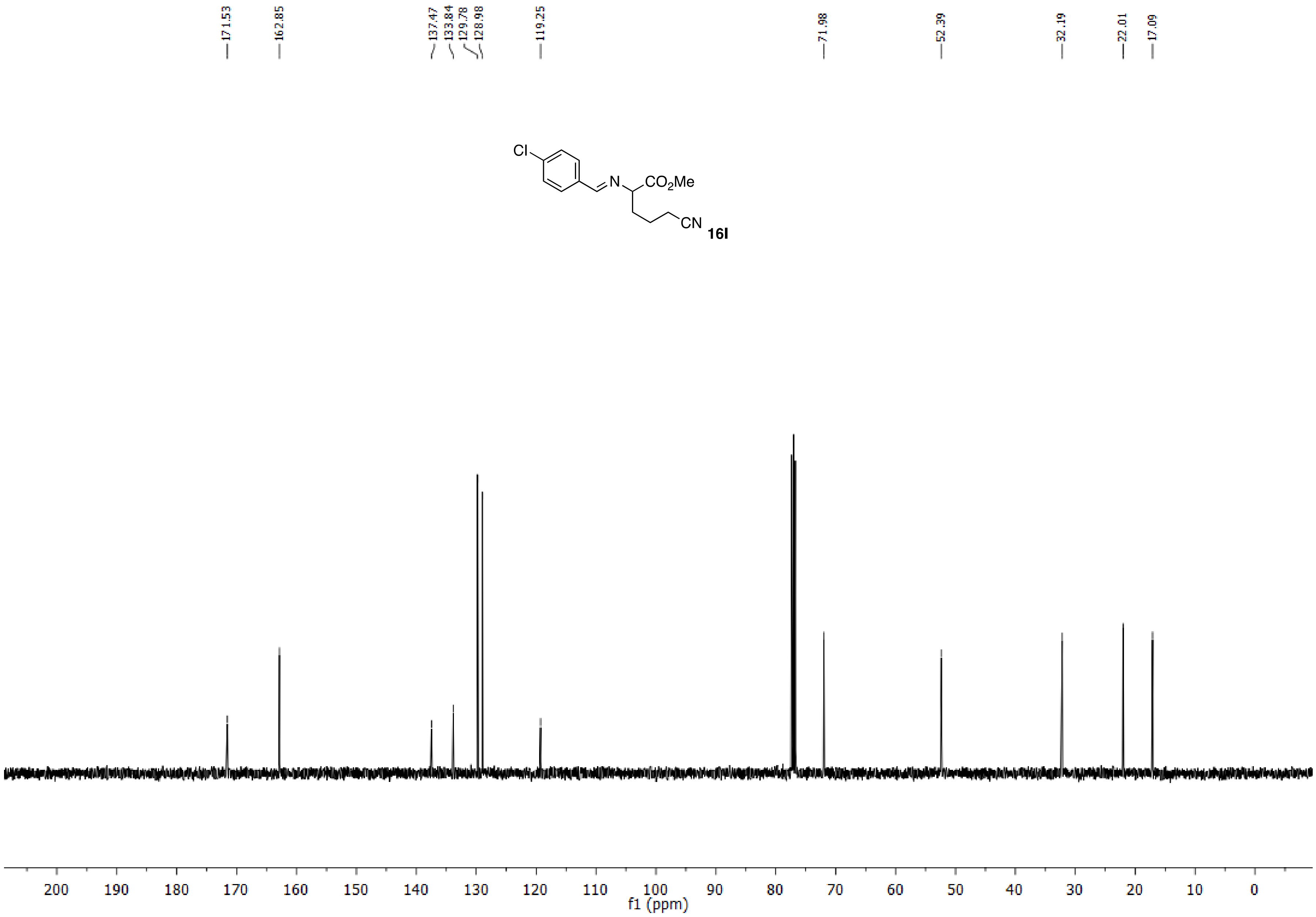
171.53
162.85
137.47
133.84
129.78
128.98
119.25
71.98
52.39
32.19
22.01
17.09
Cl
N
CO2Me
CN
16l
f1 (ppm)

Chiral HPLC Traces

Note: For each entry the top HPLC trace is a racemic sample that was prepared using an achiral cyclopropenimine

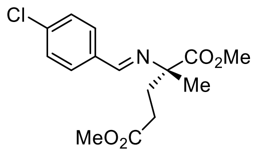

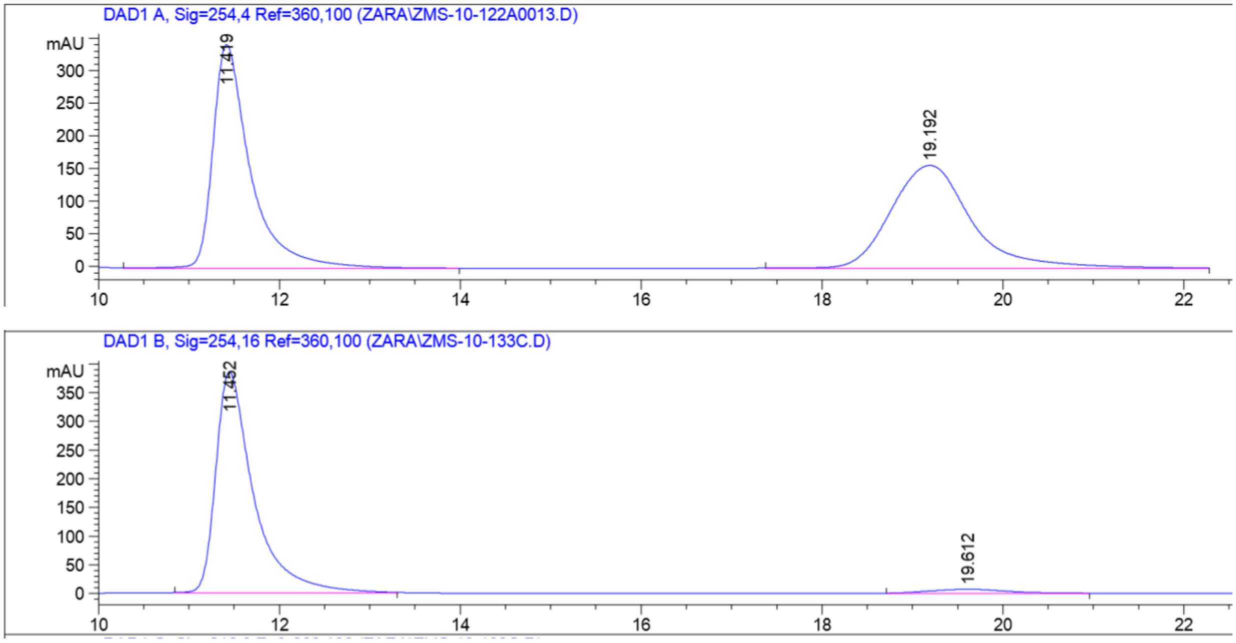

Signal 2: DAD1 B, Sig=254,16 Ref=360,100

Peak #	RetTime [min]	Type	Width [min]	Area [mAU*s]	Height [mAU]	Area %
1	11.452	BB	0.4295	1.12013e4	384.98013	96.4973
2	19.612	BB	0.8173	406.59311	7.02483	3.5027

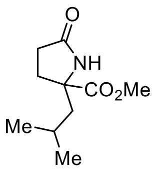

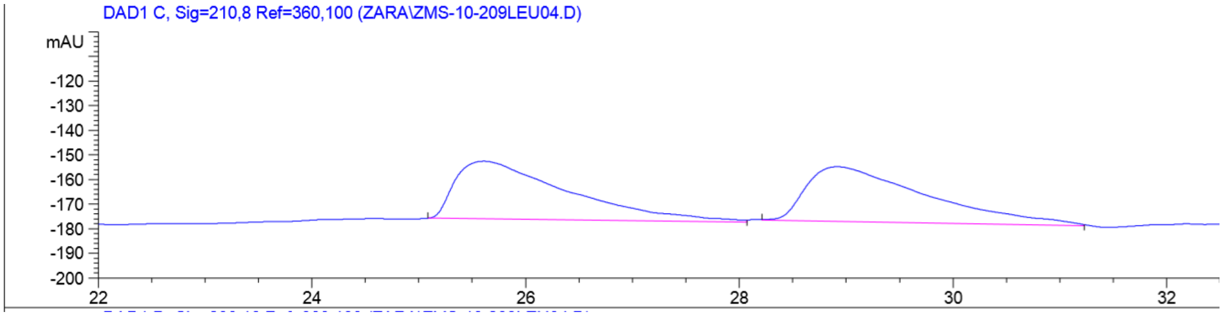

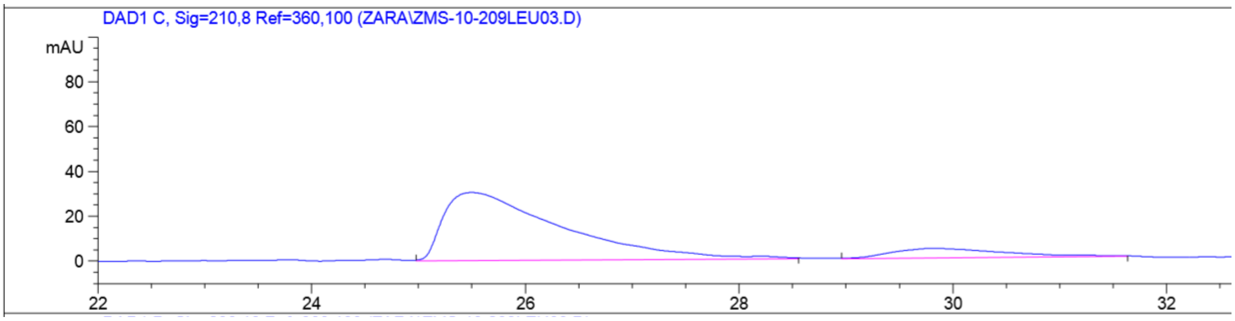

Signal 2: DAD1 C, Sig=210,8 Ref=360,100

Peak #	RetTime [min]	Type	Width [min]	Area [mAU*s]	Area %
1	25.498	MM	1.2768	2325.11548	88.5153
2	29.816	MM	1.1754	301.67957	11.4847

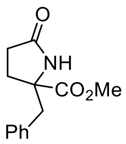

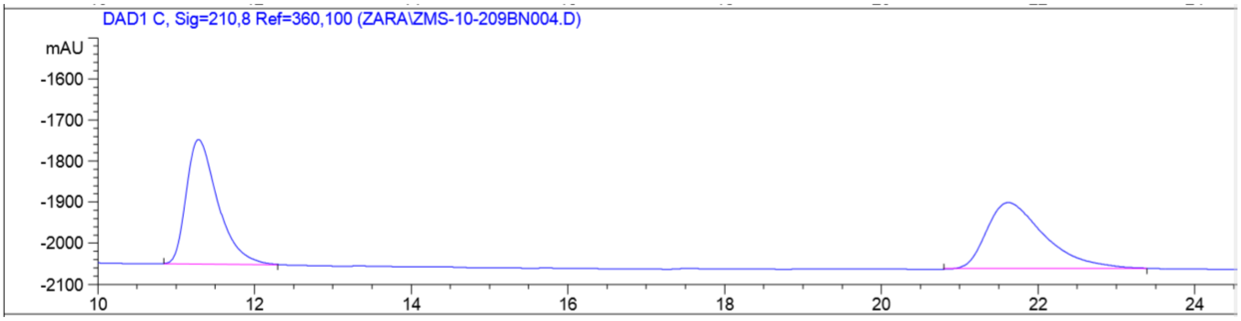

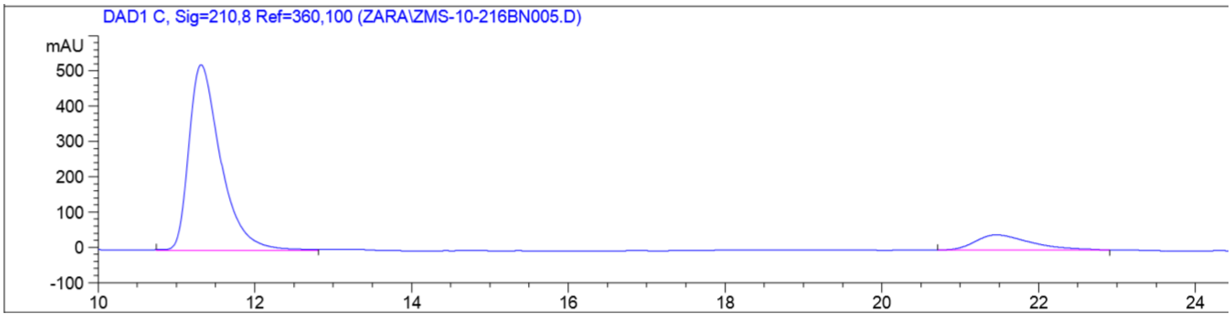

Signal 2: DAD1 C, Sig=210,8 Ref=360,100

Peak #	RetTime [min]	Type	Width [min]	Area [mAU*s]	Area %
1	11.315	MM	0.4716	1.48888e4	87.3501
2	21.460	BB	0.7410	2156.17700	12.6499
